# Laminin α5 and integrins α3 and α6 coordinately regulate collective cell migration *in vivo*

**DOI:** 10.1242/dev.205312

**Published:** 2026-06-11

**Authors:** Anna Mertens, Nicola Moratscheck, Petra A. Klemmt, Chaitanya Dingare, Melanie Heyde, Marion Basoglu, Stefan Eimer, Virginie Lecaudey

**Affiliations:** ^1^ Institute of Cell Biology and Neuroscience, Faculty of Biosciences, Goethe Universität Frankfurt, Frankfurt am Main 60438, Germany; ^2^ Developmental Biology, Institute for Biology I, Faculty of Biology, Albert-Ludwigs-Universität Freiburg, Freiburg im Breisgau 79104, Germany

**Keywords:** Basement membrane, BM, Integrin, Laminin, Collective cell migration, Lateral line primordium, pLLP, Extracellular matrix, ECM, Adhesion, Lama5, Itga6, Itga3, Zebrafish

## Abstract

Collective cell migration is essential for development and tissue homeostasis, yet how integrin-extracellular matrix adhesion coordinates migratory force generation *in vivo* remains poorly understood. Here, we have used the zebrafish posterior lateral line primordium (pLLP) as a model for epithelial collective cell migration. We show that integrins α3 and α6b are expressed in different, yet overlapping, domains of the pLLP and function redundantly to support migration. The systematic combination of *itga6b*, *itga3b* and *itga3a* mutants disrupts integrin β1 localization, increases protrusive activity and impairs migration, revealing a spatially organized, partially redundant adhesion system. We further identify laminin α5 (Lama5) as a key component of the basement membrane (BM) underlying the migrating pLLP. While loss of Lama5 alone compromises BM integrity and pLLP morphology without impairing migration, simultaneous depletion of *lama5* and *itga6b* leads to severe migration defects, with the formation of invadopodia-like structures and ultimately the stalling of migration. Together, these findings reveal a robust, redundant adhesion machinery that ensures persistent collective migration *in vivo*, and they establish fundamental principles of integrin-mediated adhesion that are relevant to development and disease.

## Teaser

**Summary:** Laminin α5 and integrins α3β1 and α6β1 form a polarized partially redundant adhesion system that ensures basement membrane coupling and enables efficient collective migration *in vivo.*

## INTRODUCTION

The migration of cells and tissues is fundamental to many stages of life, from embryogenesis, tissue regeneration and immune surveillance to cancer progression (Friedl and Gilmour, 2009; Lecaudey and Gilmour, 2006; Norden and Lecaudey, 2019). Cells push or pull on their surroundings, transmitting intracellular forces – originating from actomyosin contractions – to the extracellular matrix (ECM) via focal adhesions (FAs) (Chastney et al., 2024; De Pascalis and Etienne-Manneville, 2017). Integrins, the core FA proteins, are heterodimeric α/β transmembrane receptors that bind ECM ligands extracellularly and connect to the cytoskeleton via adaptor proteins. In mammals, 18 α- and 8 β-subunits assemble into 24 distinct integrins with distinct ligand preferences, including laminins, collagens and RGD-containing proteins (Hynes, 2002). Tissue- and context-specific expression of integrins influences the balance of actomyosin and adhesion forces (Yamada and Sixt, 2019). This allows cells to navigate complex environments with varying mechanical and chemical properties, as is the case in tumor invasion and development (Scarpa and Mayor, 2016).

Integrin-ECM adhesion is especially crucial during development (Hynes, 2002). Specialized sheet-like ECMs, termed basement membranes (BMs) provide a physical barrier along which cells orient, migrate, differentiate and signal (Alberts, 2015; Sherwood, 2021; Walma and Yamada, 2020). Heterotrimeric α/β/γ laminins are core components of BMs, initiating their assembly (Hohenester and Yurchenco, 2013) and directly interacting with integrin receptors (Belkin and Stepp, 2000). Laminins-111 and -511 are the first to be expressed and are essential for mouse embryogenesis (Klaffky et al., 2001; Li et al., 2003; Miner et al., 1998). Accordingly, loss of laminin γ1-chain (Lamc1) leads to a complete lack of BMs and inability to gastrulate (Miner et al., 2004). Mutations in *Lama5* reveal roles for laminin α5 in limb formation, neural tube closure, kidney and lung morphogenesis, and hair cell development (Miner et al., 1998; Spenlé et al., 2013). LAMA5 also contributes to tumor progression (Gordon-Weeks et al., 2019; Kikkawa et al., 2013; Qin et al., 2020). Likewise, loss of integrin-laminin adhesion underlies human diseases ranging from congenital muscular dystrophy (laminin α2-integrin α7β1 adhesion defects) (Barraza-Flores et al., 2020) to kidney dysfunction and skin blistering, due to malformations of the epidermal BM (integrin α3β1 mutations) (Has et al., 2012).

Despite extensive work in two-dimensional (2D) *in vitro* systems (Ridley et al., 2003; Vaughan and Trinkaus, 1966; Weiner et al., 1999), our understanding of how integrin-laminin adhesions function in the physical and biochemical complexity of three-dimensional (3D) *in vivo* environments remains incomplete (Mayor and Etienne-Manneville, 2016; Yamada and Sixt, 2019). As a result, fundamental questions remain about how cell–ECM adhesion is organized and regulated *in vivo*, how integrin-mediated adhesion complexes respond to tissue-scale mechanical cues (Sun et al., 2016), and how these interactions contribute to coordinated collective migration events (Ladoux and Mège, 2017).

The zebrafish posterior lateral line primordium (pLLP) provides a powerful model for addressing these questions. This cohesive cluster of cells migrates along the trunk while simultaneously undergoing proliferation, differentiation and neuromast deposition, combining features of collective migration, epithelial remodeling and morphogenesis in a 3D *in vivo* environment (Dalle Nogare and Chitnis, 2017; Ghysen and Dambly-Chaudiere, 2007). The pLLP exhibits a polarized organization with a pseudo-mesenchymal leading region (LR) and an epithelial trailing region, where neuromast precursors form (Durdu et al., 2014; Lecaudey et al., 2008). As the pLLP migrates, neuromasts are deposited sequentially and remain connected through a chain of interneuromast cells (INCs). Recently, it was shown that in a laminin γ1-containing laminin, the FA proteins integrin β1 (Itgb1a/Itgb1b) and talin are required for efficient migration of the pLLP (Yamaguchi et al., 2022). Yet, the broader molecular machinery that mediates adhesion between the pLLP and its migratory substrate remains poorly defined.

Here, we dissect the roles of integrins and laminins during pLLP migration. We identify the epithelial integrins α3a (Itga3a), α3b (Itga3b) and α6b (Itga6b) as components of a leading-trailing polarized adhesion machinery that function partially redundantly. We further show that laminin α5 is an essential component of the BM underlying migrating pLLP cells. While loss of *lama5* disrupts the integrity of the BM without significantly affecting migration, combined depletion of *lama5* and *itga6b* strongly impacts pLLP velocity and ultimately blocks its progression. Together, our findings uncover redundant integrin–laminin adhesion systems that ensure robust collective migration *in vivo*.

## RESULTS

### Integrins α3, α6 and β1b are expressed in the migrating pLLP

**Fig. 1.**
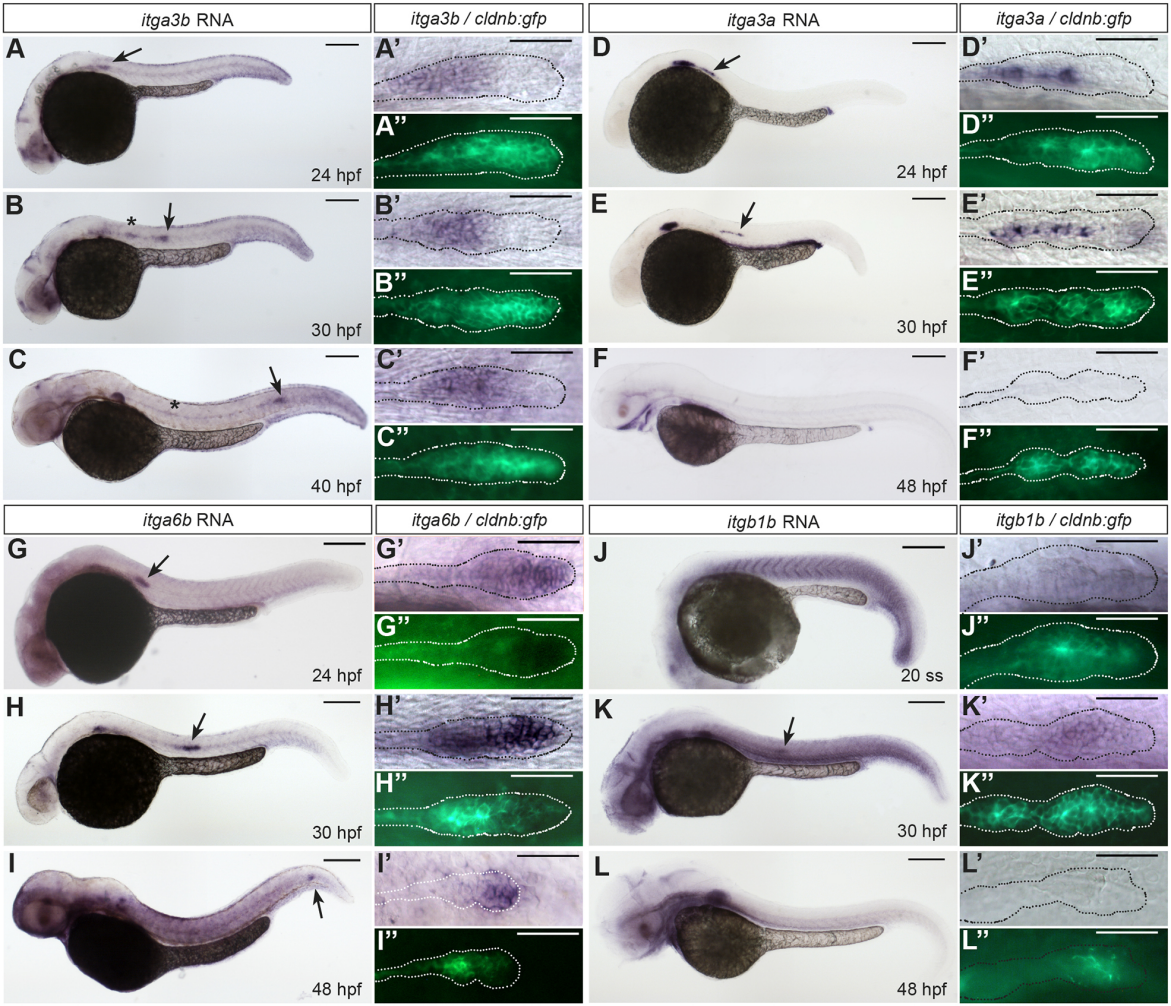
***itga3b*, *itga3a*, *itga6b* and *itgb1b* are expressed in the migrating primordium.** *cldnb:GFP* embryos were stained by *in situ* hybridization using riboprobes against *itga3b* (A-C″), *itga3a* (D-F″), *itga6b* (G-I″) and *itgb1b* (J-L″) at the indicated stages. Arrows mark the migrating pLLP. (A-L) Overview images. (A′-L′) Higher magnifications of the pLLP and (A″-L″) GFP immunostaining to delineate the pLLP. Scale bars: 200 μm in A-L; 50 μm in A′-L″.

Among the 24 integrin heterodimers described in mammals, α3β1, α6β1 and α6β4 are dominant in epithelial tissue, and α3 and α6 integrins bind laminins, the major component of BMs (Hynes, 2002). Since the pLLP cells originate from an epithelial placode (Kimmel et al., 1995; Lecaudey et al., 2008), we wanted to determine whether the corresponding zebrafish paralogs (*itga3a* and *itga3b*, and *itga6a*, *itga6b* and *itga6l*) were expressed in the pLLP during migration. *In situ* hybridization in *Tg(–0.8cldnb:lynGFP)^zf106^* (hereafter: *cldnb:GFP*) (Haas and Gilmour, 2006) showed that *itga3a* and *itga3b* were absent from the LR of the pLLP. While *itga3b* exhibited broad expression in the trailing half of the pLLP throughout migration and remained expressed in deposited neuromasts (Fig. 1A-C″), *itga3a* was observed in small clusters along the pLLP midline, with its expression restricted to early stages of migration (24 and 30 hpf) (Fig. 1D-F″). In contrast to *itga3a* and *itga3b, itga6b* expression was stronger in the LR, becoming progressively absent from the trailing region towards the end of migration (Fig. 1G-I″). While *itga6b* was expressed throughout migration (Fig. 1G-I″), its paralogues *itga6a* and *itga6l* were not expressed in the migrating pLLP (Fig. S1A-B′). *itga3a*, *itga3b* and *itga6b* were not maternally expressed (Fig. S1F-H). Integrin β1 is a major dimerization partner of integrins α3 and α6 (Hynes, 2002). We found *itgb1b*, but not *itgb1a*, expressed in the pLLP at 30 hpf but absent at 48 hpf (Fig. 1J-L″, Fig. S1C-E″), consistent with previous findings (Yamaguchi et al., 2022). Taken together, these findings indicate that three major epithelial integrins, along with their presumptive dimerization partner, are expressed in a polarized yet overlapping pattern in the migrating pLLP.

### *itga3a*, *itga3b* and *itga6b* are required during pLL development

To investigate the function of these integrins, we first analyzed the previously described *badfin* (*bdf*) mutants (*itga3b^fr21^*, Carney et al., 2010). At 40 hpf, *itga3b^−/−^* embryos exhibited no morphological defects aside from the characteristic caudal fin dysmorphogenesis (Carney et al., 2010) (Fig. S2A-C, green arrow). Neither pLLP migration (Fig. S2A-C″,J) nor morphology (Figs S3J and S4N) was affected in *itga3b^−/−^* embryos. *itga3b* mRNA levels were reduced by more than 98% in maternal zygotic (MZ) *itga3b^−/−^* mutants, suggesting that the mutant transcript is degraded (Fig. S3I) and that *badfin* is likely a null allele. Given that mutant mRNA degradation can trigger transcriptional adaptation (El-Brolosy et al., 2019), we also assessed the expression of *itga3a* and *itga6b*. *itga3a* levels were reduced and *itga6b* expression remained unchanged in MZ *itga3b^−/−^* mutants (Fig. S3I), indicating that loss of *itga3b* is not compensated for by upregulation of the expression of either its paralog *itga3a* or another laminin-binding α-integrin, *itga6b*.

Because *itga3a* is expressed in the pLLP, it may still functionally compensate for the loss of *itga3b*. To test this, we generated *itga3a* mutants using TALEN and selected an allele leading to a premature stop codon after 54 amino acids (Fig. S3A,B). *itga3a^−/−^* embryos developed normally and showed no defects in pLL development, as judged by migration distance (Fig. S2D-F″, quantification in S2K) and pLLP morphology (Figs S3K and S4N).

To assess functional redundancy between *itga3a* and *itga3b*, we generated *itga3a^fu40^;itga3b^fr21^* double mutants. Double *itga3a^−/−^;itga3b^−/−^* embryos displayed the same *bdf* caudal fin phenotype as single *itga3b^−/−^* and were otherwise indistinguishable from their siblings (Fig. S4, compare F to B-D). As in the single mutants, the pLL of double *itga3a^−/−^;itga3b^−/−^* mutants developed normally with no quantifiable change in migration distance or apparent morphological anomalies (Fig. S4F,F′, quantification in S4K). This suggests that integrin α3 activity is either not required in the pLL or its function is compensated for by yet another protein.

To investigate the function of *itga6b*, we generated TALEN mutants and selected an allele leading to a premature stop codon after 26 amino acids (*fu36*) (Fig. S3C,D). Similar to *itga3a^fu40^*, *itga6b^−/−^* embryos showed no global morphological defects and pLLP morphology was unaffected at 40 hpf (Figs S3L and S4N). Quantification of the migration distance revealed a slight delay in pLLP migration (Fig. S2G-I, quantification in L). Notably, this phenotype was subtle enough that it did not translate into a significant decrease in migration speed in 14 h time-lapse recordings (Fig. S3E-H, Movie 7).

**Fig. 2.**
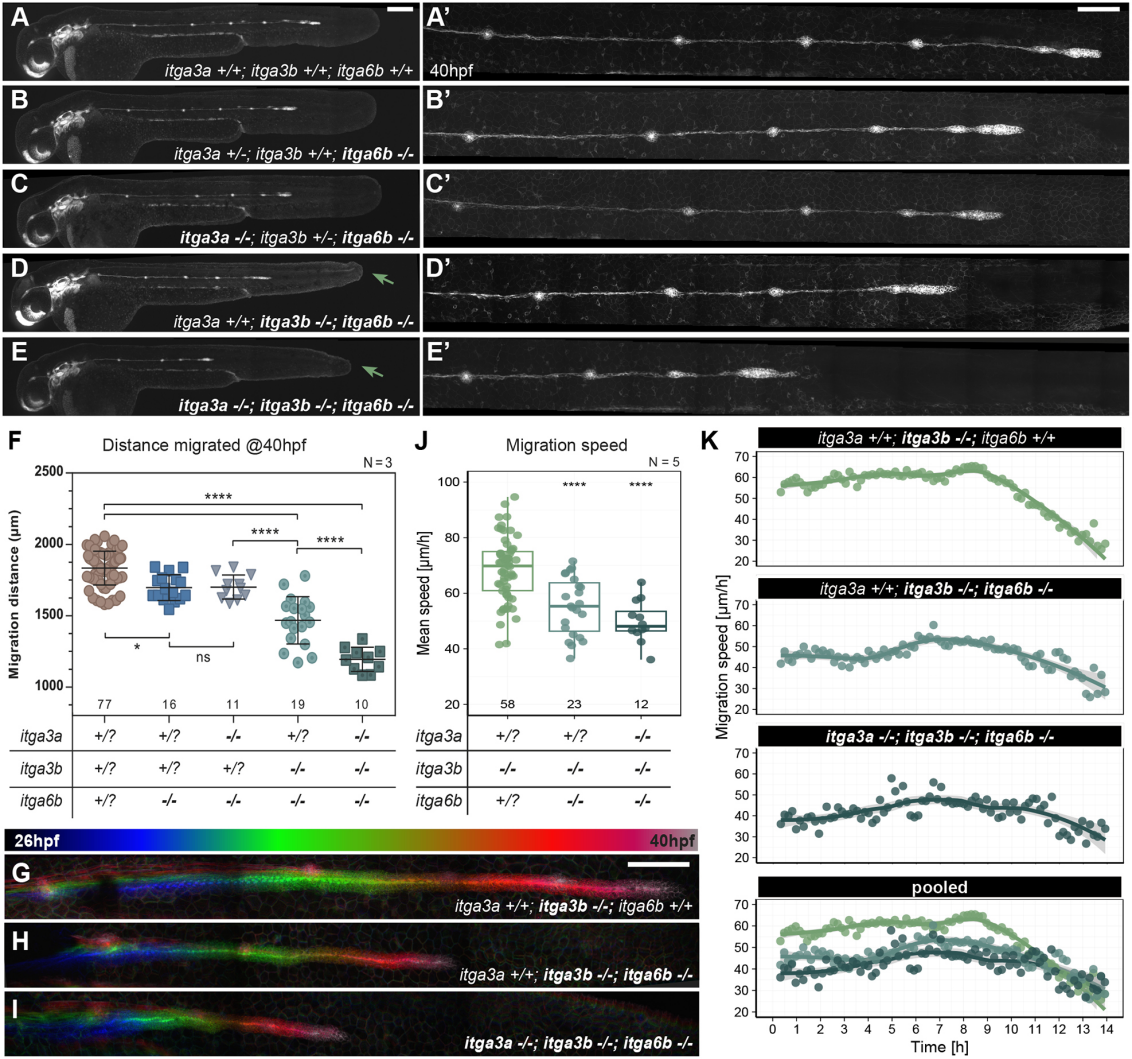
***itga3a*, *itga3b* and *itga6b* compensate for each other to mediate pLLP migration.** (A-E′) Integrin compound mutants at 40 hpf (A-E) and higher magnifications of the pLL (A′-E′). Green arrows indicate the *badfin* phenotype in *itga3b;6b* double (D) and *itga3a;3b;6b* triple (E) mutants. (F) Quantification of pLLP migration distance reveals significant delays in double and triple mutants. Full panel and quantification of triple mutant combinations are in Fig. S4. (G-K) Temporally color-coded maximum-intensity projections of 14 h time-lapse (G-I) with quantification of (J) average speed and (K) loess model of instantaneous speed with mean values per time point. (F) One-way ANOVA with multiple comparisons (each dot represents data from one embryo; data are mean±s.d.). (J) Two-tailed Wilcoxon rank-sum test [each dot represents data from one embryo; boxes indicate the median and interquartile range (IQR); whiskers indicate 1.5×IQR]. ns, non-significant; *P<0.05; ****P<0.0001. Sample sizes (*n*) are indicated above the *x*-axes. *N*, number of biological replicates. Scale bars: 200 μm in A-E,G-I; 100 μm in A′-E′.

We next wanted to test whether, in the absence of Itgα6b, the functions of Itgα3a and/or Itgα3b were required for pLLP migration and morphogenesis. For this purpose, we generated triple *itga3a;itga3b;itga6b* heterozygote mutants and analyzed their offspring. Whenever heterozygosity caused no phenotypic abnormalities (Fig. S2J-L single mutants; Fig. S4L double/triple mutants), we pooled wild-type and heterozygous individuals (indicated as ‘+/?’) to increase statistical power (Fig. 2, Fig. S4K). Single mutants from this lineage displayed phenotypes consistent with previous observations (Fig. S4C-E,K). Loss of both *itga3a* and *itga6b* did not affect overall embryo morphology (Fig. 2C) and did not enhance the mild migration delay observed in *itga6b* single mutants (compare Fig. 2B′ with 2C′, quantification in 2F).

In contrast, *itga3b^−/−^;itga6b^−/−^* double mutants (hereafter: *itga3b;6b* double mutants) exhibited a noticeably thinner caudal fin compared to the characteristic ‘*badfin’* of *itga3b* mutants (Fig. 2D, green arrow). Additionally, their pLLPs showed a significantly greater migration delay than those of *itga6b^−/−^* mutants, (compare Fig. 2D′ to 2B′, quantification in Fig. 2F). A small proportion of embryos from this cross (1/64) were *itga3a^−/−^;itga3b^−/−^;itga6b^−/−^* triple mutants (hereafter: *itga3a;3b;6b* triple mutants). Additional loss of *itga3a* further enhanced the migration phenotype of *itga3b;6b* double mutants (Fig. 2E′,F). Despite the pronounced migration delay, both double and triple mutant pLLPs ultimately completed migration (data not shown). Time-lapse recordings starting at 30 hpf confirmed the reduced migration speed throughout the entire migration process (Fig. 2G-K, Movie 1). Notably, *itga3b* expression was neither increased nor expanded in the absence of Itga3a or Itga6b, or both (Fig. S3M-P).

**Fig. 3.**
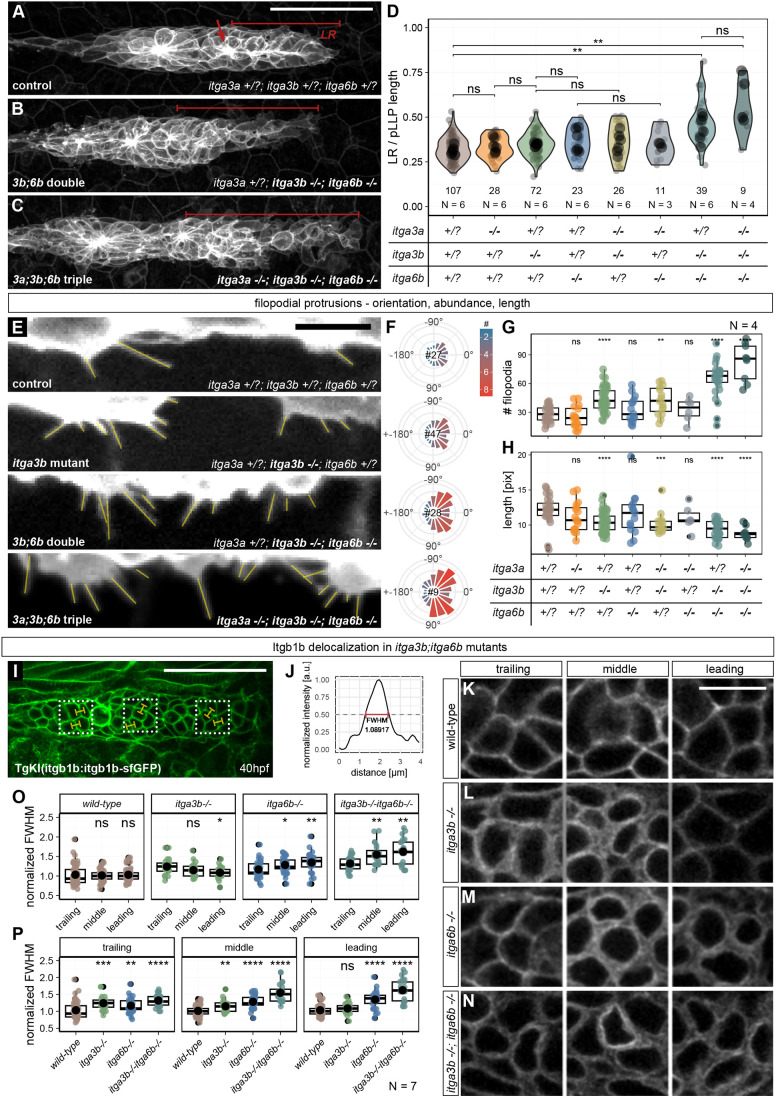
**pLLP cell behavior and Itgb1b localization are altered in α3- and α6b-integrin mutants.** (A-C) Maximum-intensity projections of pLLPs in sibling control (A), *itga3b*;*itga6b* double (B) and *itga3a;itga3b;itga6b* triple (C) mutants at 40 hpf (live). The leading region (LR) extends from the pLLP tip to the first rosette (red arrow). (D) Quantification of the ratio LR/total pLLP length. (E) Single *z*-plane higher magnification views of pLLP filopodia (translucent lines), as quantified in F-H. (F) Mean filopodia number per angular bin; 0° is the direction of migration. (G,H) Total filopodia counts (G) and mean length (H) compared across genotypes. (I,J) Quantification approach of the full-width at half-maximum (FWHM) based on signal intensity profiles (J) measured on segments (yellow in I) perpendicular to pLLP cell membranes in TgKI(*itgb1b:itgb1b-sfGFP)* pLLP (I). (K-N) *itgb1b-sfGFP* delocalization in *itga3b*;*itga6b* mutants at 40 hpf. (O,P) Quantification of *itgb1b-sfGFP* FWHM. Data are grouped by (O) genotype or (P) region for statistical comparison. (D) Pairwise Wilcoxon rank-sum tests on per-experiment means. (G,H,O,P) Two-tailed Wilcoxon test ([boxes indicate the median and interquartile range (IQR); whiskers indicate 1.5×IQR]). ns, non-significant. *P<0.05; **P<0.01; ***P<0.001; ****P<0.0001. Scale bars: 50 μm in A-C,I; 10 μm in E,K-N.

The delayed migration of integrin *3b;6b* double and triple mutants was accompanied by an extended proneuromast-free LR, both in absolute value and relative to the total length of the primordium (Fig. 3A-D, Fig. S4M). Furthermore, the protrusive activity appeared significantly increased in integrin mutants (Fig. 3B,C,E, Figs S5A, S7F, Movie 2). A quantification of all filopodia pointing outward from the pLLP (Fig. 3E, Fig. S5A,H) in *cldnb:GFP* embryos revealed that double and triple mutant pLLP cells indeed extended significantly more filopodia than control embryos (Fig. 3F,G). Unexpectedly, the number of filopodia was also increased in *itga3b* single and *itga3a;itga3b* double mutants (Fig. 3F,G, Fig. S5B,G), albeit less than in double *itga3b;itga6b* and triple mutants. Notably, this increased abundance coincided with a decrease in filopodia length (Fig. 3H, Fig. S5F) and a trend toward decreased directional persistence, as measured by the angular deviation from the direction of migration (Fig. 3F, Fig. S5B). Interestingly, leading filopodia were generally longer (Fig. S5C) and more forward-oriented (Fig. S5D) in all genotypes, except in double and triple mutants, in which leading protrusions were, on average, equivalent in length to lateral protrusions (Fig. S5C). In fact, non-biased isolation of 10% leading filopodia (Fig. S5A,C,D) demonstrated that, while differences in length, orientation and number of filopodia were largely preserved, they were significantly reduced compared to the lateral fraction (Fig. S5E-G). This suggests that the protrusiveness triggered by integrin loss is more concentrated to the pLLP midbody rather than to leading cells.

Given that integrins are core components of focal adhesions (FAs), which link actin to the ECM, we hypothesized that the increased protrusive activity in *itga3b;6b* double and *itga3a;3b;6b* triple mutants might result from impaired FA stabilization. We therefore examined phosphorylated focal-adhesion kinase (pFAK), a marker for active FAs (Jülich et al., 2015; Schober et al., 2007), and vinculin. In control pLLPs, pFAK localized in clusters at cell membranes, particularly at the periphery where signal distinction between skin and pLLP is difficult (Fig. S7A-C). Within the pLLP, pFAK signal was slightly higher in the leading than in the trailing region (Fig. S7A′-C″), consistent with the front-rear gradient of phosphorylated paxillin reported previously (Yamaguchi et al., 2022). However, we found no change in pFAK localization or intensity in *itga3b;6b* double mutants. Similarly, vinculin localized at pLLP cell membranes, was enriched at the periphery and did not reveal any obvious change in localization or intensity in the pLLPs of *itga3b;6b* double or *itga3a;3b;6b* triple mutants (Fig. S7E). Vinculin staining at the somite boundaries, however, was clearly reduced in these mutants (Fig. S7E), indicating vinculin is affected by loss of integrins there. Altogether, this analysis revealed a multilayered adhesion machinery in the pLLP with partially overlapping function for integrins α3 and α6.

### Integrin b1b localization depends on the region-specific expression of *itga3b* and *itga6b*

As all three α-integrins (3a, 3b and 6b) are expected to form functional dimers with Itgb1b, we assessed Itgb1b localization in all mutants using the *TgKI(itgb1b:itgb1b-sfGFP)^sk132^* (hereafter: *itgb1b-sfGFP*) reporter line. In wild-type controls, Itgb1b-sfGFP was uniformly localized to all pLLP cell membranes, as previously reported (Yamaguchi et al., 2022). This localization was not changed in single *itga3a* mutants (Fig. S6D,E). In *itga3b* mutants, Itgb1b-sfGFP appeared mislocalized in trailing cells, where it was less confined to the plasma membrane and instead redistributed into the cytoplasm, while remaining properly membrane restricted in leading cells (Fig. 3K-L,O-P, Fig. S6A-C,I,J). There was no significant difference between single *itga3b* and *itga3a;itga3b* double mutants (Fig. S6C,F,I). As the regionalized mislocalization of Itgb1b-sfGFP mirrored the expression of *itga3b* in the trailing region, we tested whether Itgb1b localization was also affected in *itga6b* mutants. Indeed, Itgb1b-sfGFP was strongly mislocalized upon loss of *itga6b*, more strongly in the leading and middle regions of the pLLP compared to the most trailing cells (Fig. 3M,O-P, Fig. S6G-J). *itga3b* and *itga6b* heterozygous siblings displayed similar, but less strong, mislocalization patterns (Fig. S6A,B,G,J). In double *itga3b;itga6b* mutants, Itgb1b-sfGFP was mislocalized throughout all pLLP cells; however, the defect was more severe in leading and middle regions compared with trailing cells (Fig. 3N-P, Fig. S6I,J). Overall, these results strongly imply the dimerization of Itga3b and Itga6b with Itgb1b in the pLLP.

### Lama5 is part of the basement membrane upon which pLLP cells migrate and is necessary for its integrity

**Fig. 4.**
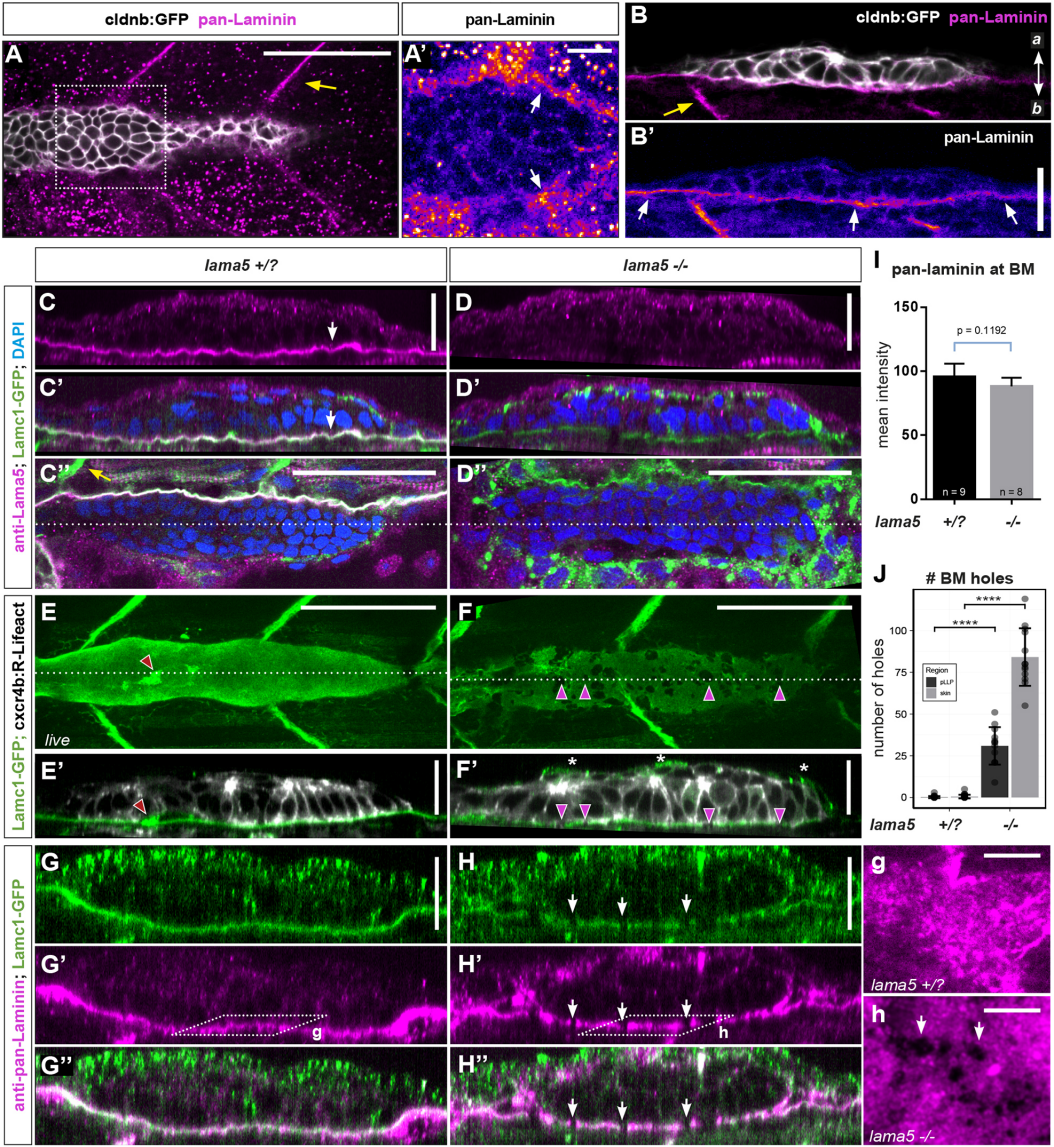
**Laminin α5 is an integral component of the pLLP migration substrate.** (A-B′) Pan-laminin immunostaining in whole-mount embryos (A,A′) and transverse cryosections (B,B′). Yellow and white arrows point to somite boundaries and the epidermal BM, respectively. Additional magnifications from A are in Fig. S8A-A″. (C-D″) Lama5 immunostaining in control (*lama5^+/?^*) (C-C″) and *lama5^−/−^* (D-D″) *lamc1-GFP* embryos. Orthogonal views (C-D′) and single *z*-planes (C″,D″) show Lama5 and Lamc1 in the epidermal BM (white arrows in C,C′) in controls. Lama5, but not Lamc1, is missing in the BM in *lama5* mutants (D-D″). (E-F′) Live imaging of *lamc1-GFP* highlighting BM holes in *lama5* mutants (purple arrowheads). (E-F′) Maximum-intensity projections of 5 μm substacks (E,F) and single orthogonal views (E′,F′). Lamc1-GFP also labels the Schwann cells of the PLLn (red arrowheads). (J) Quantification of the number of holes underneath pLLP (dark gray) and adjacent skin (light gray). (G-H″,g,h) Pan-laminin immunostaining in *lamc1-GFP* embryos. Orthogonal views (G-H″) and higher magnifications of single *z*-planes (g,h, as indicated in G′,H′), show similar discontinuities in the BM in *lama5* mutants (white arrows in H-H″, h). (I) Quantification of pan-laminin signal intensity (data are mean±s.d.). (J) Dots represent data from individual embryo (data are mean±s.d.) Unpaired, two-tailed *t*-test; ****P<0.0001. Scale bars: 50 μm for top-views in A,C″,D″,E,F; 20 μm for optical sections in B,B′,C,C′,D,D′,E′,F′,G-H″; 10 μm for higher magnifications (A′,g,h). a, apical; b, basal.

Since α3 and α6 integrins primarily bind laminins, we confirmed the presence of laminins around the pLLP by performing pan-laminin immunostaining (Fig. 4A-B′). Next to the somite boundaries (Fig. 4A,B, Fig. S8A″, yellow arrows), the antibody stained a basement membrane (BM)-like sheet beneath the pLLP (Fig. 4A′,B′, white arrows). This aligns with the reported localization of the core BM protein Lamc1 (Yamaguchi et al., 2022). To identify the specific laminin ligand involved in pLLP migration, we focused on laminin α5 (Lama5), a key α-chain component of the BM underlying epithelial tissues, and known to interact with α3- and α6-integrins (Arimori et al., 2021; Hynes, 2002; Nishiuchi et al., 2006). To determine whether Lama5 is present in the BM supporting pLLP migration, we performed immunostaining using an antibody against the mouse Lama5 protein (a generous gift from the Sorokin lab, University of Münster, Germany; Hannocks et al., 2018) in the recently established *TgBAC(lamc1:lamc1-sfGFP)^sk116Tg^* BAC reporter line (hereafter: *lamc1-GFP*) (Yamaguchi et al., 2022). Lama5 staining was detected in the BM beneath the pLLP (Fig. 4C, white arrow), where it colocalized with Lamc1-GFP (Fig. 4C′,C″). However, in contrast to Lamc1-GFP, Lama5 was absent from the somite boundaries (Fig. 4C″, yellow arrow). This result indicated that Lama5 is one of the Laminin α-subunits present in the epidermal BM over which the pLLP migrates and may function as a ligand, together with Lamc1, for integrin receptors expressed in the pLLP.

To investigate Lama5 function, we used the previously described *fransen* mutant (allele *tc17*, Carney et al., 2010). Lama5 immunostaining was absent from the epidermal BM in *lama5* mutants (Fig. 4D), while the Lamc1-GFP signal remained present (Fig. 4D′,D″). This confirmed both the antibody specificity and that the truncated protein produced by the *tc17* nonsense mutation is either degraded or not secreted. Interestingly, Lamc1-GFP formed aggregates within the basal epidermal cells of *lama5^−/−^* mutants (Fig. 4F′, white asterisks), suggesting that Lamc1 is not properly secreted in the absence of Lama5. This further implies that Lama5 is required for the proper assembly or export of Lamc1, supporting the idea that both proteins are components of the same laminin trimer. Additionally, the BM labelled by Lamc1-GFP appeared discontinuous in *lama5* mutants (Fig. 4E-H″). Live imaging confirmed the presence of holes within the otherwise continuous, sheet-like Lamc1-GFP signal (Fig. 4E-F, purple arrowheads). BM holes were not only present in the pLLP migration path but also in the surrounding epidermal BM (Fig. S8E, Fig. 4J), and were also visible with a pan-laminin immunostaining (Fig. 4H,H′, 4g,h). Yet overall pan-laminin staining intensity remained unchanged (Fig. 4g,h, quantification in Fig. 4I).

These defects were confirmed by transmission electron microscopy (TEM) analysis at 32 hpf. In control embryos, the epidermal BM was ∼40 nm thick. It contained adepidermal granules (aeg) (Fig. S8B,B′), previously described transient extracellular lipid-protein complexes appearing in the BM between 24 and 48 hpf (Weiss and Ferris, 1954; Nadol et al., 1969; Watanabe and Tachibana, 1973). In contrast, the BM of *lama5* mutants was frequently compromised by expanded, bloated segments, and, in some areas, the lamina densa was no longer discernible (Fig. S8D). Furthermore, the BM regions of wild-type thickness in *lama5* mutants lacked aegs (Fig. S8C). This is consistent with BM defects upon *col14a* knockdown that coincide with a sparser aeg distribution (Bader et al., 2013). Taken together, these results demonstrate that Lama5 is present beneath the pLLP and suggest it is required for proper Lamc1 secretion and assembly of an intact epidermal BM.

### Lama5 constitutes a substrate for the pLL but is not required for pLLP migration

**Fig. 5.**
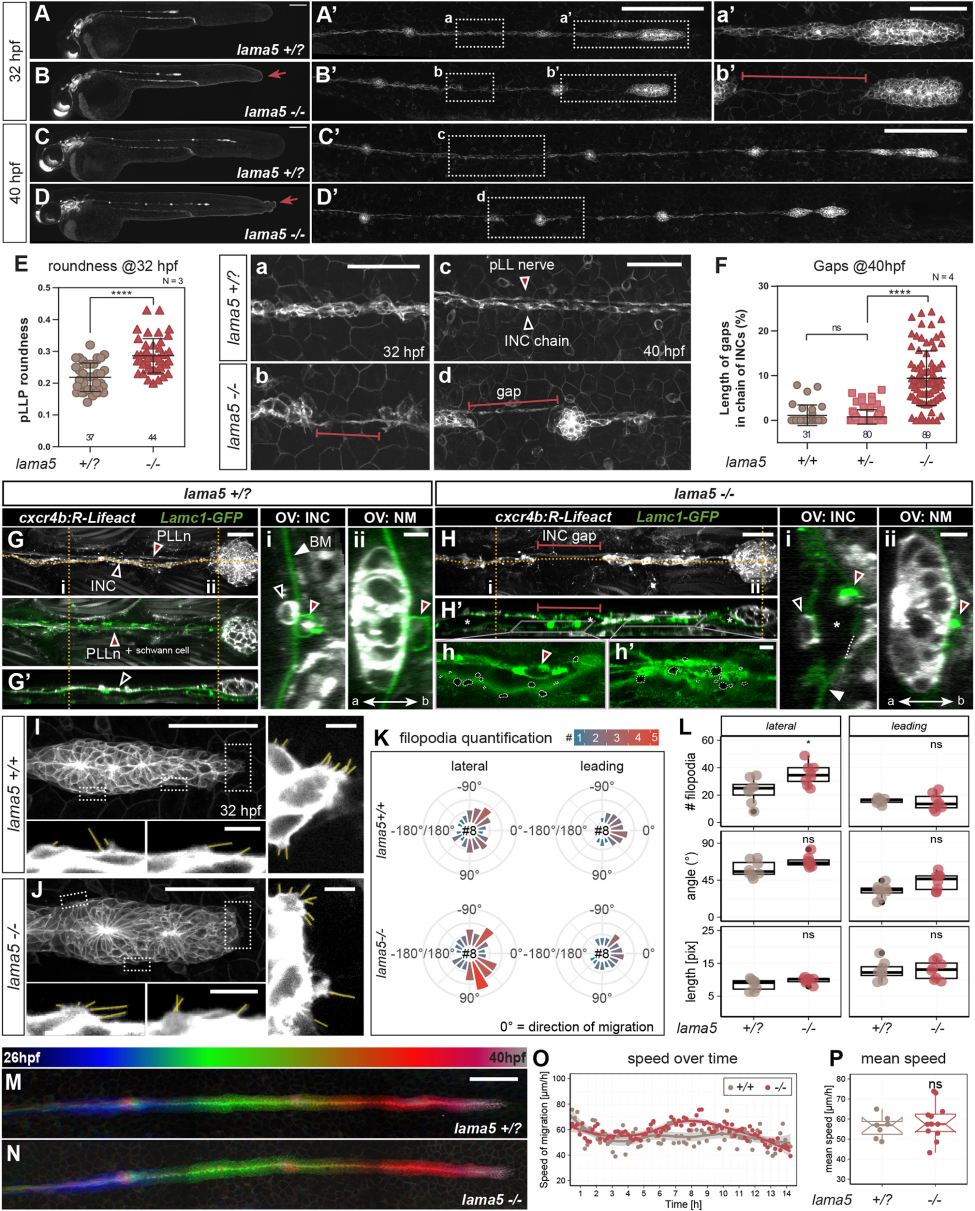
**Laminin α5 is required in different aspects of LL morphogenesis.** (A-D′) *lama5^+/?^* and *lama5*^−/−^ *cldnb:gfp* embryos fixed at 32 hpf and 40 hpf (A-D), with magnifications of the pLL (A′-D′). Red arrows highlight the *fransen* fin phenotype. (a-d) Higher magnifications of areas indicated in A′-D′ showing the disrupted INC chain in *lama5^−/−^* embryos (a-d) and pLLP (a′,b′). (E) Quantification of pLLP roundness at 32 hpf. (F) Quantification of INC gap length relative to total LL length at 40 hpf. (G,H) MIPs of INC chain (*cxcr4b:R-lifeact*, black arrowhead) and BM (*Lamc1-GFP*) in 40 hpf embryos. Orange dashed lines indicate longitudinal (G′,H′) and transverse (i,ii) digital sections. (G,i) In *lama5^+/?^* siblings, the PLLn (*cxcr4b:R-lifeact*) surrounded by Schwann cells (*Lamc1-GFP*, together PLLn^+^) has translocated below the epidermal BM, while the INC chain resides on top (red and black arrowheads). (H,H′) In *lama5^−/−^* mutants, voids are found between epidermis and BM (H′, asterisks) and translocation of the PLLn is impaired (i). BM holes (outlined) do not correlate with INC gaps (h,h′). (I,J) Filopodia in control (I) and *lama5* mutants (J). (K) Filopodia abundance binned by angle, with 0° being the direction of migration. Data are grouped in the lateral 90% and leading 10% of filopodia. Number of replicates are indicated inside the polar plots. (L) Statistical comparison of filopodia abundance, angle and length to control. (M,N) Temporally color-coded MIPs of 14-h time-lapse. (O) Loess model of instantaneous speed with mean values per time point; (P) average speed. (E,F) One-way ANOVA (E) with multiple comparisons (F) (dots indicate data from individual embryos; data are mean±s.d.). (L,P) Wilcoxon rank-sum test (dots indicate the mean of individual embryos; boxes indicate the median and IQR; whiskers indicate 1.5×IQR; in P, notches indicate 95% confidence interval). ns, non-significant. *P<0.05; ****P<0.0001. Scale bars: 200 μm in A-D′; 100 μm in M,N; 50 μm in a-d, a′,b′; 20 µm in G,H; 5 µm in Gi,Gii,Hi,Hii,h′; 5 µm in I,J (high magnification); 50 µm in I,J (low magnification). a, apical; b, basal.

During migration, the pLLP deposits neuromasts, which are connected by a chain of INCs (Fig. 5A′,a,C′,c) (Ghysen and Dambly-Chaudière, 2004). In *lama5^+/?^* embryos, INCs organized into a smooth, stable, single-layered structure adjacent to the pLL nerve (PLLn) (Fig. 5a,c and Movie 3). In contrast, INCs in *lama5^−/−^* mutants were highly destabilized, exhibiting increased protrusive activity (Fig. 5B′,b, Movie 4). This led to the formation of large gaps within the chain, leaving segments of the pLL nerve exposed (Movie 5) (Fig. 5B,b,D,d). On average, 10% of the total INC chain was interrupted by gaps in *lama5^−/−^* mutants at 40 hpf (Fig. 5F). To test if INC destabilization coincided with the discontinuity of the BM, we imaged INC gaps in Tg(*cxcr4b:R-Lifeact;Lamc1-GFP*) embryos but found no direct correlation (Fig. 5H-h′). Interestingly, however, we found large void spaces beneath the INCs in *lama5* mutant embryos (asterisks in Fig. 5H′,i), often – but not exclusively – correlating with the position of INC gaps. These voids very likely correspond to the ruptured BM observed with TEM (Fig. S8D). Notably, voids were present only in the wake path of the migrating pLLP, never in front (Fig. 4F′) or in adjacent skin tissue (Fig. S8E,a,b), suggesting defective re-adhesion of the epidermal cells with the underlying BM after separation by the migrating pLLP. On average, *lama5*^−/−^ embryos deposited half a neuromast (NM) less than controls, which is likely a consequence of shorter body axis (Fig. S8J,K). Despite severely destabilized INCs, the size and shape of NMs were not affected (Fig. S8L,M). Positioning of the PLLn, however, was aberrant in these mutants. As described previously (Raphael et al., 2010), the nerve and surrounding Schwann cells (labelled, respectively, with *R-Lifeact* and *Lamc1-GFP*) had started to gradually translocate from above the epidermal BM (lateral, Fig. S8Fii and iv) into the subepidermal space below the BM (medial) in 40 hpf control embryos (Fig. 5Gi,Gii, Fig. S8Fi, Movie 6). In contrast, the PLLn of 40 hpf *lama5* mutants resided lateral to the BM or within void regions (Fig. 5Hi), where a second BM-like structure seems to form above. Notably, the nerve often appeared medially or at least trans-BM underneath NMs in *lama5* mutants (Fig. 5Hii), suggesting that translocation might require pressure from the overlying tissue. Consistent with this, wild-type nerves that still localized lateral to the BM anterior and posterior to a NM were nevertheless found in the more-advanced trans-BM state just beneath the NM, suggesting that the latter may mechanically promote nerve translocation (Fig. S8Fiii, Movie 6).

Next, the pLLPs of *lama5^−/−^* mutants were significantly rounder throughout migration (Fig. 5a′,b′,I,J, quantification in Fig. 5E, Fig. S8I). This increased roundness coincided with a reduced LR (Fig. S8H). Furthermore, *lama5* mutant primordia extended more filopodia, specifically in the lateral domain (Fig. 5I-L), and these showed a trend of being slightly longer and less directional, while filopodia of the LR were comparable to controls in length, orientation and abundance (Fig. 5K,L). Surprisingly, despite pLLP rounding, increased number of filopodia and a discontinuous BM lacking Lama5, *lama5^−/−^* primordia migrated at speeds comparable to those of control siblings (Fig. 5M-P, Movie 7). Only a slight reduction in total migration distance at 40 hpf was detected (Fig. 5C′,D′, Fig. S8G), similar to *itga6b* mutants (Fig. S2L).

In summary, loss of Lama5 resulted, on the one hand, in destabilized and hypermotile INCs, and failure of the nerve to translocate into the subepidermal space, coinciding with defective skin (re-)adhesion along the wake path of pLLP migration. On the other hand, *lama5* mutant pLLPs displayed a rounder and shorter LR and increased filopodia extension in the rear/lateral cells. Yet, overall migration success remained largely unaffected.

### Genetic interactions between *lama5* and *itga6b* in pLL development

**Fig. 6.**
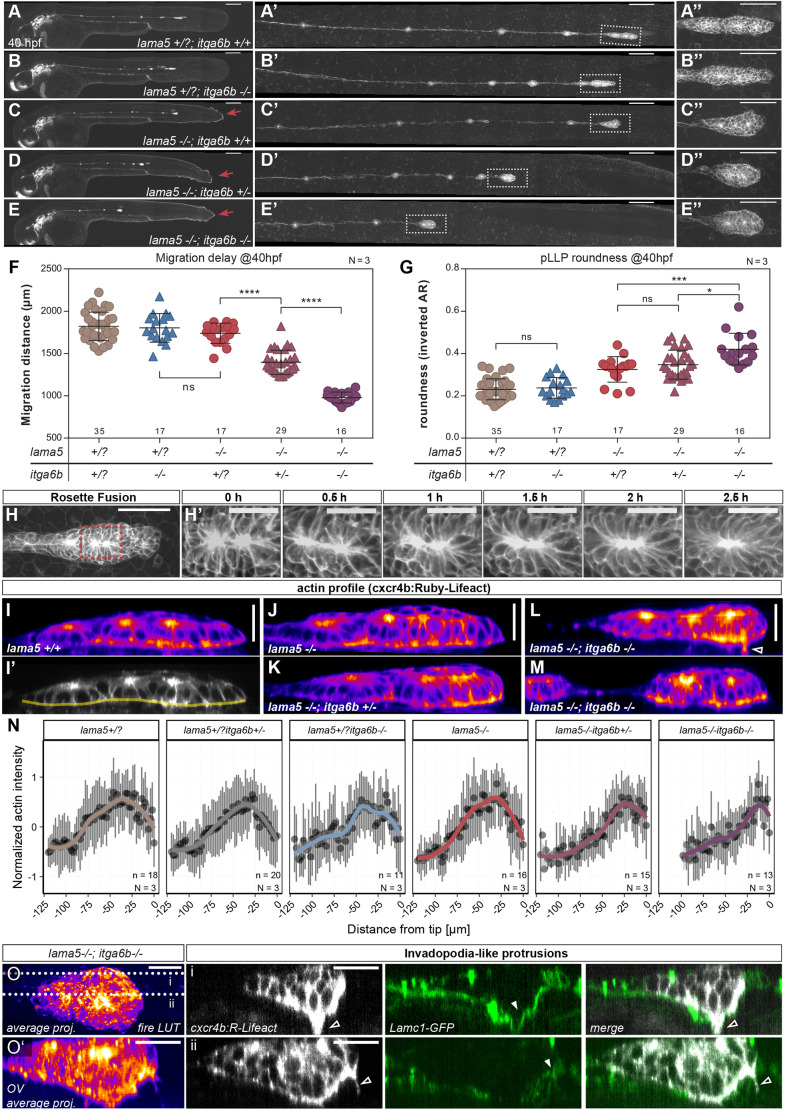
***lama5* and *itga6b* interact genetically to maintain pLLP migration.** (A-E″) *lama5;itga6b* at 40 hpf. Compared to controls (A-A″) and single mutants (B-C″), pLLP migration is impaired in *lama5^−/−^;itga6b^+/−^* mutants (D-D″) and even more severely in *lama5^−/−^;itga6b^−/−^* (E-E″). (F) Quantification of the distance migrated by the pLLP at 40 hpf. (G) Quantification of the increased roundness in compound mutants (higher magnifications of the pLLP shown in A″-E″). (H,H′) Example of a rosette fusion event in a *lama5^−/−^;itga6b^−/−^* mutant pLLP. (I-M) Supracellular actin distribution in *lama5* and *lama5;itga6b* mutants, shown are sum-projected digital sections. (N) Normalized actin signal along the basal side of the pLLP (shown in I′). Dots indicate mean values per 2 µm bin; bars indicate data range; line indicates loess model. (O,O′) Example of invadopodia-like protrusions in *lama5^−/−^;itga6b^−/−^* with digital sections indicated in O (i,ii) demonstrating penetration through BM holes (arrowheads). Sample sizes (*n*) are indicated above the *x*-axes. *N* is the number of biological replicates. One-way ANOVA with multiple comparisons (dots indicate individual embryos; bars indicate mean±s.d. ns, non-significant. *P<0.05; ***P<0.001; ****P<0.0001. Scale bars: 200 μm in A-E; 100 μm in A′-E′; 50 μm in A″-E″,H; 20 μm in I-M,O,O′; 10 μm in H′.

To test potential interactions between laminin α5 and integrin α3 and/or α6b, we generated double and triple mutants and analyzed the phenotype of their progeny. The loss of either Itga3a or Itga3b did not enhance the *lama5^−/−^* phenotype (Fig. S9E,H-I″,Q). In contrast, the combined loss of Itga6b receptor and Lama5 ligand significantly impacted pLL development. Compared to the mild migration delay of single *lama5* or *itga6b* mutants (Fig. 6A-C), loss of *lama5* together with one copy of *itga6b* resulted in a significant reduction in pLLP migration distance (Fig. 6D, quantification in F). By 40 hpf, these primordia had only migrated as far as the end of the yolk extension (Fig. 6D,D′). Despite this delay, *lama5^−/−^itga6b^+/−^* mutant primordia successfully completed migration. Strikingly, the additional loss of the second *itga6b* copy further exacerbated the migration defect, with the pLLP reaching only the middle of the yolk extension by 40 hpf (Fig. 6E,E′,F). Most notably, *lama5^−/−^;itga6b^−/−^* pLLPs stalled, failing to migrate past the end of the yolk extension even at 5 dpf (data not shown). The additional loss of *itga3a* or *itga3b* did not enhance the *lama5^−/−^;itga6b^−/−^* migration phenotype (Fig. S9O-P″), nor did depletion of both *itga3a* and *itga3b* enhance the *lama5^−/−^; itga6b^+/−^* migration phenotype (compare Fig. S9N to S9K). Quadruple mutants could not be obtained in this study.

Fourteen-hour time-lapse recordings starting at 30 hpf revealed that *lama5^−/−^; itga6b^−/−^* primordia exhibited reduced migration speed from the onset (Fig. S10I-M, Movie 7). While wild-type and single mutants migrated at about 60 μm/h, double mutants showed a markedly reduced mean speed of 35 μm/h (Fig. S10M). Notably, their speed progressively declined from 40 μm/h to below 20 μm/h, consistent with the stalling phenotype observed at later stages (Fig. S10L, Movie 7). In comparison, *lama5^−/−^;itga6b^+/−^* mutants displayed an intermediate phenotype, with moderately reduced speeds relative to controls but significantly higher than double mutants (Fig. S10J,M, Movie 7). The migration defect observed in *lama5;itga6b* mutant primordia was associated with an extremely rounded morphology (Fig. 6D-E″), more severe than that observed in *lama5* single mutants (Fig. 6C, quantification in G). This phenotype was also evident in the time-lapse recordings (Fig. S10L, Movie 7). Together, this synergistic phenotype suggests that *lama5* and *itga6b* function in parallel adhesion systems that partially compensate for each other.

Despite impaired migration and pronounced rounding, *lama5;itga6b* mutant primordia retained dynamically protrusive activity in the direction of migration (Fig. S10G-H′, Movie 8). In some instances, the leading edge appeared to split, as if attempting to bypass an obstacle (Fig. S10C, Movie 9). We also observed fusion of rosette centers, likely reflecting persistent pushing forces from the rear, combined with impaired forward extension (Fig. 6H,H′, Movie 10). In line with this interpretation, rosette fusion has been linked to the slowing down of leading cells, while cells at the rear end continue to migrate (Mukherjee et al., 2025 preprint).

To gain further insight into the migratory behavior of *lama5;itga6b* mutants, we analyzed supracellular actin distribution along the basal side of the pLLP in the *cxcr4b:R-Lifeact* reporter line (Fig. 6I,I′). Interestingly, we found actin distributed in a consistent bell-shaped pattern: lower in the rear than in the front, peaking between 25 to 50 μm from the leading tip, where the intensity drops again (Fig. 6N). This pattern, including the relative peak position, was conserved in *lama5* and *itga6b* single mutants, but strongly shifted towards the LR in *lama5;itga6b* double mutants (Fig. 6N). The actin peak position corresponds to the pLLP middle region, as defined by Yamaguchi et al. (2022), who show that myosin II levels are highest in the rear where the pLLP also exerts the most force.

Finally, we found that *lama5;itga6b* double mutant pLLPs extended large cellular protrusions directed to the BM (Fig. 6L,O,O′, Movie 11). We will refer to these protrusions as invadopodia-like as they meet neither all podosome nor all invadopodia criteria (Linder et al., 2023). These invadopodia-like protrusions often reached through the earlier described holes in *lama5* mutant BM (Fig. 4F), deep into the subepidermal tissue (Movie 12), or pushed the BM down (Fig. 6Oii). We did not observe invadopodia-like structures in single *lama5* mutants (Figs 4F′ and 6J), suggesting that loss of *itga6b* specifically promotes this straying behavior.

Overall, these results demonstrate that the combined loss of *lama5* and *itga6b* leads to extensive tissue rounding and the formation of invadopodia-like protrusions, resulting in severely impaired migration and pLLP stalling. To our knowledge, such a stalling of the pLLP has not been reported previously.

## DISCUSSION

In the present study, we provide a comprehensive analysis of integrin-laminin adhesion in a collectively migrating epithelium *in vivo*. Through systematic genetic analysis, we have uncovered that migration of the pLLP involves a polarized but partially redundant integrin adhesion machinery comprising integrins α3 and α6. We further identify laminin α5 as a key component of the extracellular substrate and a strong genetic interaction between *lama5* and *itga6b*. Thus, we uncover a dual adhesion system that ensures robust collective migration and provide general principles of epithelial tissue dynamics *in vivo*.

### Composition of the pLLP migration substrate

We show here for the first time that laminin α5 is an essential component of the epidermal BM supporting pLLP migration. In *lama5* mutants, Lamc1-GFP accumulates in epidermal cells, which is consistent with the secretion of laminin β/γ-chains requiring pre-assembly with an α-chain and formation of heterotrimers (Yurchenco et al., 1997). In mammals, the α5-chain trimerizes with β1 or β2 and γ1-3 chains (Aumailley, 2013). In zebrafish, *lamb1a*, *lamb4* and *lamc1* are expressed in the embryonic epidermis (Farnsworth et al., 2020; Parsons et al., 2002; Sur et al., 2023; Sztal et al., 2011), consistent with the presence of laminin-511, a prominent epithelial laminin, in the epidermal BM during pLLP migration.

*lama5* mutants displayed holes in the otherwise sheet-like BM labelled by Lamc1-GFP. This is consistent with discontinuous BM formation in *Lama5*-deficient murine (Miner et al., 1998; Li et al., 2003; Fukumoto et al., 2006) and zebrafish (Webb et al., 2007) epithelia, altogether demonstrating the essential role of Lama5 in establishing intact epithelial BMs.

The presence of a discontinuous BM indicates partial compensation by other γ1-containing trimers. In mice, loss of Laminin α5 is partially compensated for by upregulation of laminin α1, α2 or α4 chains (Miner et al., 2004). Additionally, *lama2* overexpression in muscle rescues myelination defects in lateral line Schwann cells in zebrafish, implying that the underlying muscle may contribute to the epidermal BM (Mikdache et al., 2022). Further investigation will be necessary to elucidate the composition and origin of the embryonic epidermal BM, in wild type and *lama5* mutants.

### Dual function of laminin α5 in the lateral line

Our findings reveal different functions for Lama5 in the context of lateral line development: (1) stabilizing the chain of INCs and (2) promoting extension of the pLLP LR. Essentially, Lama5 seems to both restrict and drive migratory behavior. One possible explanation is the polarized receptor expression, with *itga6b* enriched in the leading and/or middle region while *itga3b* is restricted to the trailing region, the INC and deposited neuromasts. Such spatially regulated integrin expression resembles ‘integrin switching’, a mechanism known to modulate cell adhesivity and migration (Madamanchi et al., 2014; Meighan and Schwarzbauer, 2008; Truong and Danen, 2009).

Our observations that both *lama5* and *itga3a*/*itga3b* mutants exhibited slightly increased protrusive activity of the lateral and/or rear pLLP cells and loss of Itga3 function did not worsen the *lama5* mutant phenotype suggest that Itga3a and Itga3b are candidate receptors for Lama5. However, complete loss of Itga3 function did not produce any INC defects, indicating that the Itga3-Lama5 interaction is not strictly required for INC stabilization.

Our data further suggest a skin re-adhesion defect following separation of the epidermis and the underlying BM by the migrating pLLP. Thus, we speculate that the effect of Lama5 on INCs may be indirect: weakened epidermis–BM adhesion due to the loss of Lama5 could account for the hypermotility of the INCs due to lack of mechanical confinement. Similarly, our data suggest that the translocation of the pLL nerve into subepidermal space may require mechanical pressure from the skin.

### Polarized integrin expression coordinates migration

Our systematic genetic analysis revealed contrasting phenotypes: pronounced rounding in *lama5;itga6b* mutants versus elongated and highly protrusive pLLPs in double and triple integrin mutants. Single *lama5* mutants exhibited a rounder pLLP with a reduced LR, suggesting that Lama5 engages with a receptor active in the LR. However, the absence of similar rounding in *itga3a*, *itga3b* or *itga6b* mutants (single, double or triple), argues against these α-integrins being the primary Lama5 receptors in the LR. Notably, loss of Itgβ1, the most ubiquitous β-integrin subunit, results in delayed migration and pLLP rounding (Yamaguchi et al., 2022), resembling the *lama5* mutant phenotype. Identifying the Lama5 receptor(s), integrin or not, operating in the LR will require further investigation.

Intriguingly, loss of *itga3* and *itga6b* significantly affects Itgb1b localization within their respective expression domains, which overlap in the middle of the pLLP. This result confirms the existence of a polarized adhesion machinery within the pLLP, and suggests that Itga3b and Itga6b dimerize with Itgb1b. Yet, loss of Itga3 and/or Itga6b results in a morphological phenotype distinct from the rounding of *itgb1b* mutants. This supports the existence of another β1-integrin heterodimer, involving other α-subunits, that contributes to Lama5-dependent LR extension. However, we cannot exclude the possibility of the presence of additional adhesion systems, integrin based or integrin independent, within the pLLP. *itgb4* and *itgb6* are expressed in the pLLP (Yamaguchi et al., 2022), but also non-integrin laminin-binding proteins, such as Dag1, Bcam and heparan-sulfate proteoglycans (Thisse et al., 2004, 2008; Venero Galanternik et al., 2015), suggesting that these molecules also contribute to the complex adhesion machinery.

On the other hand, combined loss of *lama5* and *itga6b* produced a strong synergistic phenotype – slower migration, extreme tissue rounding and invadopodia-like structures – already apparent upon loss of a single *itga6b* allele, consistent with partial Itgb1b mislocalization in *itga6b* heterozygotes. This genetic interaction indicates that Itga6b largely compensates for the absence of Lama5 by binding alternative ECM components, implying that Lama5 and Itga6b function in parallel, partially redundant adhesion systems. Importantly, invadopodia-like protrusions were not observed in single *lama5* mutants, which already display a discontinuous BM, suggesting that loss of *itga6b* promotes aberrant protrusive behavior in *lama5;itga6b* mutants. We speculate that loss of Itga6b shifts the balance of remaining adhesion receptors, which may aberrantly redirect cells toward subepidermial cues, such as the chemokine *cxcl12a* expressed by the horizontal myoseptum (Ghysen and Dambly-Chaudière, 2004).

### Compatibility with non-canonical ‘rear-pushing model’

The classic model of cell migration emphasizes traction forces arising at the leading edge of cells and cell collectives (Scarpa and Mayor, 2016). However, rear-driven mechanisms have also been observed. For example, cranial neural crest cell migration in zebrafish and *Xenopus* is driven by a supracellular actomyosin cable at the back of the cluster (Shellard et al., 2018), and elegant *in vivo* traction force microscopy experiments have recently shown that the pLLP exerts highest stresses in the rear-to-middle region, rather than at the front (Yamaguchi et al., 2022).

Our data support and extend this model. Genetic analyses reveal reciprocal functional compensation between *itga3* and *itga6b*, without evidence for transcriptional adaptation, thus suggesting true functional overlap. *itga3* and/or *itga6b* expression (*in situ* hybridization) and activity (Itgb1b mislocalization) overlap in a domain in the middle of the pLLP, suggesting that this might represent the crucial domain of integrin activity for migration force generation. In turn, we interpret extensive stretching of the LR in double and triple integrin mutants as a compensatory response to reduced pushing forces in the middle/rear of the pLLP. Similar LR elongation has been observed when trailing cell migration is impaired, such as upon disruption of Cxcr7 or Fgf signaling (Lecaudey et al., 2008; Nechiporuk and Raible, 2008; Valentin et al., 2007), and more recently when trailing cells are selectively slowed (Mukherjee et al., 2025 preprint). The increased amount of filopodial protrusions in double and/or triple integrin mutants, but also in *lama5* mutants, further raises questions about the role of filopodia during migration, which may range from chemical to mechanical sensation, e.g. guiding pLLP migration through chemo- or durotaxis, respectively. For instance, ectopic and less directional filopodia could reflect increased probing of the environment due to the lack of chemical of mechanical input in integrin and *lama5* mutants.

That the reduced LR of *lama5* single mutant, exacerbated by additional loss of *itga6b*, did not affect the ability of these primordia to initiate migration, further supports that migration force originates behind the LR. However, under a purely rear-driven mechanism, we could expect the pLLP to continue migration under these circumstances. Instead, we observed progressive rounding, rosette fusion and formation of invadopodia-like structures until pLLP migration eventually and reliably stalled. Similarly, selective slowing of leading cells by interference with *cxcl12* expression causes compacted pLLP morphology with rosette fusion events (Mukherjee et al., 2025 preprint). Together, these observations suggest that while force generation originates in the rear, efficient migration requires proper extension of the LR to translate these forces into forward movement.

Our analysis of actin distribution further supports this model. Basal actin levels peak in the middle region of the pLLP. According to Yamaguchi et al. (2022), the highest forces would be exerted immediately behind this region. In *lama5;itga6b* mutants that fail to properly extend their LR, we observe a shifted actin peak from the middle to the leading cells. Thus, we propose that sufficient extension of the LR in the direction of migration is required to direct rear-generated pushing forces into forward motion, possibly by acting mechanically and biochemically as a wedge to separate the skin from the BM.

In conclusion, we uncovered two partially redundant cell-substrate adhesion systems that together ensure robustness and coordination of collective epithelial migration. One is laminin α5 dependent, likely engages an as-yet unidentified receptor in the LR and possibly integrin α3a/b in the trailing region. The other relies on integrin α6b in the leading-to-middle region, which binds alternative ECM ligand(s), and largely compensates for the loss of laminin α5. Building on the concept of rear-driven migration, we propose a model in which rear-generated forces are transmitted through a spatially organized adhesion network to leading cells that channel and translate this force into forward movement. These results establish fundamental principles of epithelial adhesion and collective migration with implications for development, tissue homeostasis, and pathological processes such as cancer invasion

### Study limitations

Despite these advances, several limitations should be considered. First, the cellular and mechanical basis of the observed phenotypes remains incompletely resolved. Direct measurements of force generation and transmission (e.g. traction force microscopy or atomic force microscopy), as well as analysis of actin flow or focal adhesion dynamics, would further refine the proposed model integrating rear-generated forces and LR adhesion. Second, the molecular basis of Itgb1b mislocalization and its functional consequences remain unclear. Finally, the identity and role of the invadopodia-like protrusions observed in *lama5;itga6b* mutants require further characterization. These analyses are constrained by the low frequency and lack of prospective identification of homozygous mutants in complex crosses, but represent important directions for future work.

## MATERIALS AND METHODS

### Zebrafish lines and maintenance

Procedures involving animals were carried out according to the guidelines of the Goethe University of Frankfurt and were approved by the German authorities (Veterinary Department of the Regional Board of Darmstadt). Zebrafish embryos were obtained through natural spawning and staged as previously described (Kimmel et al., 1995). *bdf^fr21^* (*badfin/itga3b*) and *fra^tc17^* (*fransen/lama5*) (Carney et al., 2010) mutant lines, and *Tg(-0.8cldnb:lynEGFP)^zf106^* (*cldnb:GFP*, Haas and Gilmour, 2006), *TgBAC(lamc1:lamc1-sfGFP)^sk116^* (*lamc1-GFP*, Yamaguchi et al., 2022), *TgKI(itgb1b:itgb1b-sfGFP)^sk132^* (*itgb1b-sfGFP*) (Yamaguchi et al., 2022) and *Tg(cxcr4b(FOS):Lifeact-mRuby)^fu16^* (referred to as *cxcr4b:R-Lifeact*; Lardennois et al., 2026) transgenic lines have been described previously. The alleles *itga3a^fu40^* and *itga6b^fu36^* were generated using TALEN targeting exon 1, as previously described (Agarwala et al., 2015; Dingare et al., 2018).

### Genotyping

To genotype mutant alleles, gDNA was extracted from fins or single embryos by boiling at 95°C in 50 mM NaOH and neutralizing with 1/10 volume 1 M Tris-HCl (pH 8.0). Allele-specific PCRs were carried out using 1 μl gDNA using the primers and according annealing temperatures listed in Table S1. Following PCR, the *fra^tc17^* allele was genotyped by sequencing to identify the single nucleotide change (AAAAA>AATAA). Sequencing was performed by Eurofins GATC Supreme Run Sequencing. The *itga3b^fr21^* allele was genotyped with the dCAPS (derived cleaved amplified polymorphic sequences) technique (Neff et al., 1998) and digested with EcoRV-HF, yielding 231 bp/26 bp products. The *itga3a^fu40^* allele was genotyped by digest with HhaI yielding 240 bp/198 bp products. The *itga6b^fu36^* allele was genotyped by digest with MboI yielding 353 bp/212 bp products.

### RNA extraction, cDNA synthesis and qPCR

RNA extraction was performed using TRIzol reagent according to the manufacturer's instructions. Embryos at 32 hpf were dechorionated and homogenized in 500 μl TRIzol with a blunt needle tip syringe (20-40 embryos per tube).

cDNA was generated using 1 μg of total RNA with the Invitrogen SuperscriptIII First-Strand Synthesis System for *in situ* hybridization probe synthesis and iScript cDNA Synthesis Kit (Bio-Rad, 1708890) for qPCR, according to the manufacturer's instructions.

For qPCR, cDNA was diluted 1:1. Primers for qPCR were designed using the Primer3-based Primer-BLAST tool from the NCBI. Real-time qPCR was performed using the Bio-Rad iTaq Universal SYBR Green Supermix (1725270) according to the manufacturer's instructions in a CFX Connect Real-Time System (Bio-Rad) running 40 cycles of 15 s denaturation (95°C) and 60 s annealing+extension (60°C). Fold changes were calculated using the 2^ΔΔCt^ method (Livak and Schmittgen, 2001). Expression of the genes of interest were normalized to that of *rpl13*.

### Embryo fixation, *in situ* hybridization and immunostaining

In general, embryos were fixed with 4% PFA in 1×PBS overnight at 4°C or 2 h at room temperature. After fixation, embryos were washed in PBS and either used directly for experiments or dehydrated in methanol/PBS series and stored at −20°C for *in situ* hybridization.

Whole-mount immunostaining was performed according to standard procedures (Lecaudey et al., 2004). The following primary antibodies were used: anti-GFP (mouse monoclonal, 1:500, Takara Bio, 632380, RRID:AB_10013427), anti-pan-laminin (rabbit, 1:400, Sigma-Aldrich, L9393, RRID:AB_477163), anti-pFAK1 (rabbit, 1:200, Invitrogen, 44-624G), anti-vinculin (mouse, 1:200, Sigma-Aldrich, SAB4200729, RRID:AB_2877646) and anti-lama5 (rabbit, 1:200, #504; Hannocks et al., 2018). Anti-rabbit and anti-mouse conjugated antibodies were used as secondary antibodies (1:500, Alexa Fluor 488 (anti-GFP) and 647 (others), Molecular Probes).

To generate riboprobes for *in situ* hybridization, partial cDNA sequences for *itga3a*, *itga3b*, *itga6a*, *itga6b*, *itga6l*, *itgb1a* and *itgb1b* were amplified by PCR using the primers listed in Table S1. PCR products were subcloned in pGEM-T-easy (Promega) and digoxigenin (DIG)-labelled riboprobes were transcribed from linearized plasmid using Sp6 (Thermo Fisher) or T7 polymerases (Thermo Fisher). *In situ* hybridization was performed according to standard procedures (Lecaudey et al., 2004) with probes diluted 1:100 and followed by immunostaining using anti-GFP antibody, as explained above.

### Electron microscopy

Embryos were fixed in 2.5% glutaraldehyde in 0.1 M cacodylate buffer (pH 7.2) overnight at 4°C. Fixed samples were washed twice in 0.1 M cacodylate buffer containing 3% sucrose, post-fixed in 1% reduced osmium tetroxide, dehydrated in ethanol and embedded in Araldite resin. Ultrathin sections (50 nm) were cut using an ultramicrotome (Leica), and ribbons of sections were mounted on Pioloform-coated copper slot grids. Contrast was enhanced with 5% uranyl acetate in methanol/water followed by lead citrate. Micrographs were acquired using a Zeiss TEM 900 transmission electron microscope operated at 80 keV in bright-field mode and equipped with a Tröndle 2K camera.

### Imaging

#### Mounting

For live imaging, embryos were dechorionated using pronase and mounted in 0.3% low-melting agarose (LMPA) in an agarose cast (Kleinhans and Lecaudey, 2019), with the following modifications: no second layer of 0.5% LMPA was added after polymerization.

#### *In situ* hybridization images

Imaging of *in situ* hybridization embryos was performed on an inverted microscope (Ti-S, Nikon) using a 4× air objective to acquire whole embryos and a 20× air objective for close-ups of the pLLP.

#### Spinning disc microscopy

Imaging of live and fixed embryos was performed on a Nikon W1 Spinning Disc Microscope with the following objectives: 10× air objective (NA 0.45, WD 4 mm), 20× (NA 0.95,WD 0.95 mm) and 40× (NA 1.15,WD 0.60 mm) water objectives.

For 14 h timelapse recordings, embryos were selected at 26-30 hpf, mounted as described above and imaged using the 20× objective with additional 1.5× digital zoom. To ensure water-immersion of the objective over the course of the timelapse, a water dispenser was manually installed. An incubation chamber surrounding the microscope kept embryos at 28°C during the experiment. The 488 nm laser was set to 20% power, 30 ms exposure time, 2×2 binning and gain 4. Images were acquired as *z*-stacks with 5 μm *z*-spacing at 10 min frame interval.

### Image processing

Images were processed using the open-source processing software Fiji (Fiji Is Just ImageJ) (2.1.0/1.53c). For presentation purposes, three-dimensional *z*-stacks were generally flattened by maximum intensity projection (MIP). For immunostainings, single *z*-slices were selected, and signal intensities were adjusted for better visualization of the antibody signal. In experiments with control and mutant groups, signal intensities were adjusted in the same way for all images. Quantifications of intensity, length or roundness were performed using segmented line or freehand selection tools in Fiji. Quantifications performed in Fiji were further processed and plotted in R (version 4.3.1) using the ggplot2 package (version 3.4.3) or GraphPad Prism (version 6.07).

#### Segmentation and analysis of 14 h time-lapses

Analysis of 14 h timelapses was performed using a two-part custom-written macro in Fiji. First, the migrating pLLP and deposited neuromasts were automatically segmented from each frame using “MaxEntropy” thresholding, generating a binary video. Mis-segmentations due to the skin and kidney also being labelled by *cldnb:GFP*, were manually corrected by the user. Second, this binary movie was subjected to the “Analyze particles” plug-in and the pLLP was registered as the particle with highest X coordinate. Finally, the position of the pLLP leading edge was determined using bounding rectangle coordinates and roundness was measured. The instantaneous speed between frames and mean speed was then calculated in R. Plots of speed and roundness over-time were generated using the ggplot2 package and trends visualized by locally estimated scatterplot smoothing (loess) curves. Mean speeds were plotted as boxplots overlayed by individual datapoints.

#### Quantification of filopodial protrusions

Filopodia were quantified in whole *z*-stacks of primordia labelled with cldnb:GFP. Settings were adjusted to ensure filopodia were visible, which results in oversaturating most of the image, and primordia were manually oriented horizontally so that filopodial orientation could be expressed relative to the direction of migration. Any linear protrusion projecting outward from the pLLP was measured, regardless of whether it originated from a lamellipodium or directly from a cell body. This inclusive approach was chosen to avoid bias introduced by the stretched and protrusive morphology observed in double *itga3b;6b* and triple *itga3a;3b;6b* integrin mutants. Filopodia were manually traced using a custom Fiji tool: the user defined each filopodium by clicking its start and end points using the line ROI tool, recording XY coordinates for all filopodia across the entire *z*-stack and exporting the results as CSV files. Filopodial length was calculated as the Euclidean distance between start and end coordinates: √((x_2_−x_1_)^2^+(y_2_−y_1_)^2^) and is reported in pixels. Orientation was calculated using the two-argument arctangent function (atan2(dy, dx)), yielding angles in radians converted to degrees, where 0° corresponds to the direction of migration, ±90° perpendicular orientations and ±180° to the opposite direction. Absolute orientation values were used for boxplot comparisons between genotypes and ±180° values for polar plot visualization.

#### FWHM analysis of Itgb1b-sfGFP mislocalization

To quantify Itgb1b-sfGFP localization at the cell membrane, line ROIs of ∼5 μm were drawn perpendicular to the cell membrane in three defined regions of the pLLP: leading, middle and trailing. Five to ten ROIs were drawn per region per embryo using a semi-automated Fiji macro to reduce operator error. Fluorescence intensity profiles along each ROI were exported as CSV files and processed in R. To enable comparison between embryos heterozygous and homozygous for the itgb1b-sfGFP reporter, intensity values were normalized to a 0-1 scale prior to analysis. Further, 0.2 μm were trimmed from each end of the intensity profile to exclude edge artifacts. Then, the full width at half maximum (FWHM) was calculated for each normalized profile. Mean FWHM was then calculated per region per embryo and used for statistical comparisons. A broader FWHM indicates a more diffuse membrane signal, interpreted as mislocalization of Itgb1b-sfGFP away from the cell membrane.

#### Quantification of supracellular actin distribution using cxcr4b:R-Lifeact

Actin distribution along the basal side of the pLLP was quantified from XZ re-slices generated in Fiji using the Reslice function (top projection, slice interval 0.4 μm). Three segmented line regions of interest (ROIs) were drawn manually per embryo from the leading tip to the rear of the pLLP, with a ROI thickness of 10 pixels. Fluorescence intensity profiles were extracted and exported as CSV files. In R, intensity values were *z*-score normalized per embryo prior to analysis to account for variability in reporter expression between individuals, focusing the analysis on distribution pattern rather than absolute intensity levels. Position was expressed as distance from the leading tip in μm (negative values indicating distance toward the rear). Intensity profiles were interpolated onto a common grid spanning −150 to 0 μm in 2 μm steps using linear interpolation. Per-ROI profiles were then averaged per embryo, and mean normalized intensity per position was calculated across embryos per genotype. The resulting distribution was visualized as a scatter plot with a loess smoothing curve.

### Statistics and reproducibility

Sample sizes were determined based on standard deviation consistency and prior experience with the system. Experimental groups were determined by PCR-based genotyping performed after data acquisition. For the majority of genotypes, embryos were imaged prior to genotyping and group allocation was therefore blind to the investigator. Exceptions include *lama5* and *itga3b* single mutants, which display visible fin phenotypes from 32 hpf and 40 hpf, respectively, and *lama5;itga6b, itga3b;6b* double and *itga3a;3b;6b* triple mutants, which display visible migration delays from ∼32 hpf. For these genotypes, embryos were enriched by phenotypic pre-selection prior to imaging at the relevant stages. Embryos imaged prior to these stages were processed blindly regardless of genotype. Outliers were identified and excluded using the Tukey method (1.5× interquartile range rule) per genotype group. The procedure was applied up to a maximum of three rounds.

Statistical analysis in R was performed using the ggpubr package (version 0.6.0) with ‘stat_compare_means’ function for the non-parametric two-sided Wilcoxon rank-sum test used in most plots (also known as Mann–Whitney *U*-test) and ‘stat_cor’ to add Pearson correlation coefficients (Figs S5C,D and S7D). For pairwise comparisons (Fig. 3D and Fig. S4M,N), ‘pairwise_wilcox_test’ from the rstatix package (version 0.7.2) was used. For the mixed effects models in Fig. S4K,L, linear mixed-effects models were fitted using the lme4 package (version 1.1.35.1) with lmerTest (version 3.1.3) for *P*-value estimation. Models included genotype as fixed effect and experimental id (=biological replicate N) as a random effect to account for day-to-day variability (formula: y∼genotype+(1|experiment_id)). Pairwise comparisons of estimated marginal means were performed using the emmeans package (version 1.10.6), with Tukey correction for multiple comparisons.

For plots generated in GraphPad prism (Figs 2F, 4I,J, 5E,F, 6F,G, Figs S2J-L, S8G,H,J,L and S9Q), normality was assessed using the Shapiro-Wilk test and, if confirmed, comparisons were made using one-way ANOVA with or without multiple comparisons. If normality was not confirmed, the Mann–Whitney *U*-test was used.

### AI assistance

AI-based language tools (Claude, Anthropic; ChatGPT, OpenAI) were used to assist in R code development and manuscript revision. All scientific content, interpretation and conclusions are the authors' own.

## Supplementary Material



10.1242/develop.205312_sup1 Supplementary information

## References

[DEV205312C1] Agarwala, S., Duquesne, S., Liu, K., Boehm, A., Grimm, L., Link, S., König, S., Eimer, S., Ronneberger, O. and Lecaudey, V. (2015). Amotl2a interacts with the Hippo effector Yap1 and the Wnt/β-catenin effector Lef1 to control tissue size in zebrafish. *eLife* 4, e08201. 10.7554/eLife.08201 26335201 PMC4596637

[DEV205312C2] Alberts, B. (2015). *Molecular Biology of the Cell*, 6th edn. New York, NY: Garland Science, Taylor and Francis Group.

[DEV205312C3] Arimori, T., Miyazaki, N., Mihara, E., Takizawa, M., Taniguchi, Y., Cabañas, C., Sekiguchi, K. and Takagi, J. (2021). Structural mechanism of laminin recognition by integrin. *Nat. Commun.* 12, 4012. 10.1038/s41467-021-24184-8 34188035 PMC8241838

[DEV205312C4] Aumailley, M. (2013). The laminin family. *Cell Adhes. Migr.* 7, 48-55. 10.4161/cam.22826 PMC3544786 23263632

[DEV205312C5] Bader, H. L., Lambert, E., Guiraud, A., Malbouyres, M., Driever, W., Koch, M. and Ruggiero, F. (2013). Zebrafish collagen XIV is transiently expressed in epithelia and is required for proper function of certain basement membranes. *J. Biol. Chem.* 288, 6777-6787. 10.1074/jbc.M112.430637 23325806 PMC3591589

[DEV205312C6] Barraza-Flores, P., Bates, C. R., Oliveira-Santos, A. and Burkin, D. J. (2020). Laminin and integrin in LAMA2-related congenital muscular dystrophy: from disease to therapeutics. *Front. Mol. Neurosci.* 13, 1. 10.3389/fnmol.2020.00001 32116540 PMC7026472

[DEV205312C7] Belkin, A. M. and Stepp, M. A. (2000). Integrins as receptors for laminins. *Microsc. Res. Tech.* 51, 280-301. 10.1002/1097-0029(20001101)51:3<280::AID-JEMT7>3.0.CO;2-O 11054877

[DEV205312C8] Carney, T. J., Feitosa, N. M., Sonntag, C., Slanchev, K., Kluger, J., Kiyozumi, D., Gebauer, J. M., Coffin Talbot, J., Kimmel, C. B., Sekiguchi, K. et al. (2010). Genetic analysis of fin development in zebrafish identifies furin and hemicentin1 as potential novel fraser syndrome disease genes. *PLoS Genet.* 6, 22. 10.1371/journal.pgen.1000907 PMC2855323 20419147

[DEV205312C9] Chastney, M. R., Kaivola, J., Leppänen, V.-M. and Ivaska, J. (2024). The role and regulation of integrins in cell migration and invasion. *Nat. Rev. Mol. Cell Biol.* 26, 147-167. 10.1038/s41580-024-00777-1 39349749

[DEV205312C10] Dalle Nogare, D. and Chitnis, A. B. (2017). A framework for understanding morphogenesis and migration of the zebrafish posterior Lateral Line primordium. *Mech. Dev.* 148, 69-78. 10.1016/j.mod.2017.04.005 28460893 PMC10993927

[DEV205312C11] De Pascalis, C. and Etienne-Manneville, S. (2017). Single and collective cell migration: the mechanics of adhesions. *MBoC* 28, 1833-1846. 10.1091/mbc.e17-03-0134 28684609 PMC5541834

[DEV205312C12] Dingare, C., Niedzwetzki, A., Klemmt, P. A., Godbersen, S., Fuentes, R., Mullins, M. C. and Lecaudey, V. (2018). The Hippo pathway effector Taz is required for cell morphogenesis and fertilization in zebrafish. *Development* 145, dev167023. 10.1242/dev.167023 30327325 PMC6262795

[DEV205312C13] Durdu, S., Iskar, M., Revenu, C., Schieber, N., Kunze, A., Bork, P., Schwab, Y. and Gilmour, D. (2014). Luminal signalling links cell communication to tissue architecture during organogenesis. *Nature* 515, 120-124. 10.1038/nature13852 25337877

[DEV205312C14] El-Brolosy, M. A., Kontarakis, Z., Rossi, A., Kuenne, C., Günther, S., Fukuda, N., Kikhi, K., Boezio, G. L. M., Takacs, C. M., Lai, S.-L. et al. (2019). Genetic compensation triggered by mutant mRNA degradation. *Nature* 568, 193-197. 10.1038/s41586-019-1064-z 30944477 PMC6707827

[DEV205312C15] Farnsworth, D. R., Saunders, L. M. and Miller, A. C. (2020). A single-cell transcriptome atlas for zebrafish development. *Dev. Biol.* 459, 100-108. 10.1016/j.ydbio.2019.11.008 31782996 PMC7080588

[DEV205312C16] Friedl, P. and Gilmour, D. (2009). Collective cell migration in morphogenesis, regeneration and cancer. *Nat. Rev. Mol. Cell Biol.* 10, 445-457. 10.1038/nrm2720 19546857

[DEV205312C17] Fukumoto, S., Miner, J. H., Ida, H., Fukumoto, E., Yuasa, K., Miyazaki, H., Hoffman, M. P. and Yamada, Y. (2006). Laminin α5 is required for dental epithelium growth and polarity and the development of tooth bud and shape. *J. Biol. Chem.* 281, 5008-5016. 10.1074/jbc.M509295200 16365040

[DEV205312C18] Ghysen, A. and Dambly-Chaudière, C. (2004). Development of the zebrafish lateral line. *Curr. Opin. Neurobiol.* 14, 67-73. 10.1016/j.conb.2004.01.012 15018940

[DEV205312C19] Ghysen, A. and Dambly-Chaudiere, C. (2007). The lateral line microcosmos. *Genes Dev.* 21, 2118-2130. 10.1101/gad.1568407 17785522

[DEV205312C20] Gordon-Weeks, A., Lim, S. Y., Yuzhalin, A., Lucotti, S., Vermeer, J. A. F., Jones, K., Chen, J. and Muschel, R. J. (2019). Tumour-derived laminin α5 (LAMA5) promotes colorectal liver metastasis growth, branching angiogenesis and notch pathway inhibition. *Cancers* 11, 630. 10.3390/cancers11050630 31064120 PMC6562694

[DEV205312C21] Haas, P. and Gilmour, D. (2006). Chemokine signaling mediates self-organizing tissue migration in the zebrafish lateral line. *Dev. Cell* 10, 673-680. 10.1016/j.devcel.2006.02.019 16678780

[DEV205312C22] Hannocks, M.-J., Pizzo, M. E., Huppert, J., Deshpande, T., Abbott, N. J., Thorne, R. G. and Sorokin, L. (2018). Molecular characterization of perivascular drainage pathways in the murine brain. *J. Cereb. Blood Flow Metab.* 38, 669-686. 10.1177/0271678X17749689 29283289 PMC5888861

[DEV205312C23] Has, C., Spartà, G., Kiritsi, D., Weibel, L., Moeller, A., Vega-Warner, V., Waters, A., He, Y., Anikster, Y., Esser, P. et al. (2012). Integrin α_3_ mutations with kidney, lung, and skin disease. *N. Engl. J. Med.* 366, 1508-1514. 10.1056/NEJMoa1110813 22512483 PMC3341404

[DEV205312C24] Hohenester, E. and Yurchenco, P. D. (2013). Laminins in basement membrane assembly. *Cell Adh. Migr.* 7, 56-63. 10.4161/cam.21831 23076216 PMC3544787

[DEV205312C25] Hynes, R. O. (2002). Integrins. *Cell* 110, 673-687. 10.1016/S0092-8674(02)00971-6 12297042

[DEV205312C26] Jülich, D., Cobb, G., Melo, A. M., McMillen, P., Lawton, A. K., Mochrie, S. G. J., Rhoades, E. and Holley, S. A. (2015). Cross-scale integrin regulation organizes ECM and tissue topology. *Dev. Cell* 34, 33-44. 10.1016/j.devcel.2015.05.005 26096733 PMC4496283

[DEV205312C27] Kikkawa, Y., Ogawa, T., Sudo, R., Yamada, Y., Katagiri, F., Hozumi, K., Nomizu, M. and Miner, J. H. (2013). The lutheran/basal cell adhesion molecule promotes tumor cell migration by modulating integrin-mediated cell attachment to laminin-511 protein. *J. Biol. Chem.* 288, 30990-31001. 10.1074/jbc.M113.486456 24036115 PMC3829412

[DEV205312C28] Kimmel, C. B., Ballard, W. W., Kimmel, S. R., Ullmann, B. and Schilling, T. F. (1995). Stages of embryonic development of the zebrafish. *Dev. Dyn.* 203, 253-310. 10.1002/aja.1002030302 8589427

[DEV205312C29] Klaffky, E., Williams, R., Yao, C.-C., Ziober, B., Kramer, R. and Sutherland, A. (2001). Trophoblast-specific expression and function of the integrin α7 subunit in the peri-implantation mouse embryo. *Dev. Biol.* 239, 161-175. 10.1006/dbio.2001.0404 11784026

[DEV205312C30] Kleinhans, D. S. and Lecaudey, V. (2019). Standardized mounting method of (zebrafish) embryos using a 3D-printed stamp for high-content, semi-automated confocal imaging. *BMC Biotechnol.* 19, 68. 10.1186/s12896-019-0558-y 31640669 PMC6805687

[DEV205312C31] Ladoux, B. and Mège, R.-M. (2017). Mechanobiology of collective cell behaviours. *Nat. Rev. Mol. Cell Biol.* 18, 743-757. 10.1038/nrm.2017.98 29115298

[DEV205312C32] Lardennois, A., Duda, V., Dingare, C., Klemmt, P. A., Heinzen, C., Desruelles, L., Heyde, M. ,Kleinhans, D., Falk, T., Schelmbauer, C. et al. (2026). Vgll4 proteins limit organ size in zebrafish through Yap1-dependent and -independent mechanisms. *Commun. Biol.*. 9, 574. 10.1101/2025.05.08.652796 42032219 PMC13110368

[DEV205312C33] Lecaudey, V. and Gilmour, D. (2006). Organizing moving groups during morphogenesis. *Curr. Opin. Cell Biol.* 18, 102-107. 10.1016/j.ceb.2005.12.001 16352429

[DEV205312C34] Lecaudey, V., Anselme, I., Rosa, F. and Schneider-Maunoury, S. (2004). The zebrafish Iroquois gene *iro7* positions the r4/r5 boundary and controls neurogenesis in the rostral hindbrain. *Development* 131, 3121-3131. 10.1242/dev.01190 15175248

[DEV205312C35] Lecaudey, V., Cakan-Akdogan, G., Norton, W. H. J. and Gilmour, D. (2008). Dynamic Fgf signaling couples morphogenesis and migration in the zebrafish lateral line primordium. *Development* 135, 2695-2705. 10.1242/dev.025981 18599504

[DEV205312C36] Li, S., Edgar, D., Fässler, R., Wadsworth, W. and Yurchenco, P. D. (2003). The role of laminin in embryonic cell polarization and tissue organization. *Dev. Cell* 4, 613-624. 10.1016/S1534-5807(03)00128-X 12737798

[DEV205312C37] Linder, S., Cervero, P., Eddy, R. and Condeelis, J. (2023). Mechanisms and roles of podosomes and invadopodia. *Nat. Rev. Mol. Cell Biol.* 24, 86-106. 10.1038/s41580-022-00530-6 36104625

[DEV205312C38] Livak, K. J. and Schmittgen, T. D. (2001). Analysis of relative gene expression data using real-time quantitative PCR and the 2−ΔΔCT method. *Methods* 25, 402-408. 10.1006/meth.2001.1262 11846609

[DEV205312C39] Madamanchi, A., Zijlstra, A. and Zutter, M. M. (2014). Flipping the switch: integrin switching provides metastatic competence. *Sci. Signal.* 7, pe9. 10.1126/scisignal.2005236 24667375 PMC4209128

[DEV205312C40] Mayor, R. and Etienne-Manneville, S. (2016). The front and rear of collective cell migration. *Nat. Rev. Mol. Cell Biol.* 17, 97-109. 10.1038/nrm.2015.14 26726037

[DEV205312C41] Meighan, C. M. and Schwarzbauer, J. E. (2008). Temporal and spatial regulation of integrins during development. *Curr. Opin. Cell Biol.* 20, 520-524. 10.1016/j.ceb.2008.05.010 18603422 PMC2572561

[DEV205312C42] Mikdache, A., Boueid, M.-J., Lesport, E., Delespierre, B., Loisel-Duwattez, J., Degerny, C. and Tawk, M. (2022). Timely Schwann cell division drives peripheral myelination *in vivo* via the laminin/cAMP pathway. *Development* 149, dev200640. 10.1242/dev.200640 35938454

[DEV205312C43] Miner, J. H., Cunningham, J. and Sanes, J. R. (1998). Roles for laminin in embryogenesis: exencephaly, syndactyly, and placentopathy in mice lacking the laminin alpha5 chain. *J. Cell Biol.* 143, 1713-1723. 10.1083/jcb.143.6.1713 9852162 PMC2132973

[DEV205312C44] Miner, J. H., Li, C., Mudd, J. L., Go, G. and Sutherland, A. E. (2004). Compositional and structural requirements for laminin and basement membranes during mouse embryo implantation and gastrulation. *Development* 131, 2247-2256. 10.1242/dev.01112 15102706

[DEV205312C45] Mukherjee, A., Hilzendeger, M., Rinvelt, A., Fatma, S., Schupp, M., Dalle Nogare, D. and Chitnis, A. B. (2025). Signaling and mechanics influence the number and size of epithelial rosettes in the migrating zebrafish posterior lateral line primordium. *bioRxiv*. 10.1101/2025.05.17.650326 PMC13405218 42367001

[DEV205312C46] Nadol, J. B., Gibbins, J. R. and Porter, K. R. (1969). A reinterpretation of the structure and development of the basement lamella: An ordered array of collagen in fish skin. *Dev. Biol.* 20, 304-331. 10.1016/0012-1606(69)90017-7 5384082

[DEV205312C47] Nechiporuk, A. and Raible, D. W. (2008). FGF-dependent mechanosensory organ patterning in zebrafish. *Science* 320, 1774-1777. 10.1126/science.1156547 18583612

[DEV205312C48] Neff, M. M., Neff, J. D., Chory, J. and Pepper, A. E. (1998). dCAPS, a simple technique for the genetic analysis of single nucleotide polymorphisms: experimental applications in *Arabidopsis thaliana* genetics. *Plant J.* 14, 387-392. 10.1046/j.1365-313X.1998.00124.x 9628033

[DEV205312C49] Nishiuchi, R., Takagi, J., Hayashi, M., Ido, H., Yagi, Y., Sanzen, N., Tsuji, T., Yamada, M. and Sekiguchi, K. (2006). Ligand-binding specificities of laminin-binding integrins: a comprehensive survey of laminin–integrin interactions using recombinant α3β1, α6β1, α7β1 and α6β4 integrins. *Matrix Biol.* 25, 189-197. 10.1016/j.matbio.2005.12.001 16413178

[DEV205312C50] Norden, C. and Lecaudey, V. (2019). Collective cell migration: general themes and new paradigms. *Curr. Opin. Genet. Dev.* 57, 54-60. 10.1016/j.gde.2019.06.013 31430686

[DEV205312C51] Parsons, M. J., Pollard, S. M., Saúde, L., Feldman, B., Coutinho, P., Hirst, E. M. A. and Stemple, D. L. (2002). Zebrafish mutants identify an essential role for laminins in notochord formation. *Development* 129, 3137-3146. 10.1242/dev.129.13.3137 12070089

[DEV205312C52] Qin, Y., Shembrey, C., Smith, J., Paquet-Fifield, S., Behrenbruch, C., Beyit, L. M., Thomson, B. N. J., Heriot, A. G., Cao, Y. and Hollande, F. (2020). Laminin 521 enhances self-renewal via STAT3 activation and promotes tumor progression in colorectal cancer. *Cancer Lett.* 476, 161-169. 10.1016/j.canlet.2020.02.026 32105676

[DEV205312C53] Raphael, A. R., Perlin, J. R. and Talbot, W. S. (2010). Schwann cells reposition a peripheral nerve to isolate it from postembryonic remodeling of its targets. *Development* 137, 3643-3649. 10.1242/dev.057521 20876648 PMC2964096

[DEV205312C54] Ridley, A. J., Schwartz, M. A., Burridge, K., Firtel, R. A., Ginsberg, M. H., Borisy, G., Parsons, J. T. and Horwitz, A. R. (2003). Cell migration: integrating signals from front to back. *Science* 302, 1704-1709. 10.1126/science.1092053 14657486

[DEV205312C55] Scarpa, E. and Mayor, R. (2016). Collective cell migration in development. *J. Cell Biol.* 212, 143-155. 10.1083/jcb.201508047 26783298 PMC4738384

[DEV205312C56] Schober, M., Raghavan, S., Nikolova, M., Polak, L., Pasolli, H. A., Beggs, H. E., Reichardt, L. F. and Fuchs, E. (2007). Focal adhesion kinase modulates tension signaling to control actin and focal adhesion dynamics. *J. Cell Biol.* 176, 667-680. 10.1083/jcb.200608010 17325207 PMC2064024

[DEV205312C57] Shellard, A., Szabó, A., Trepat, X. and Mayor, R. (2018). Supracellular contraction at the rear of neural crest cell groups drives collective chemotaxis. *Science* 362, 339-343. 10.1126/science.aau3301 30337409 PMC6218007

[DEV205312C58] Sherwood, D. R. (2021). Basement membrane remodeling guides cell migration and cell morphogenesis during development. *Curr. Opin. Cell Biol.* 72, 19-27. 10.1016/j.ceb.2021.04.003 34015751 PMC8530833

[DEV205312C59] Spenlé, C., Simon-Assmann, P., Orend, G. and Miner, J. H. (2013). Laminin α5 guides tissue patterning and organogenesis. *Cell Adhes. Migr.* 7, 90-100. 10.4161/cam.22236 PMC3544791 23076210

[DEV205312C60] Sun, Z., Guo, S. S. and Fässler, R. (2016). Integrin-mediated mechanotransduction. *J. Cell Biol.* 215, 445-456. 10.1083/jcb.201609037 27872252 PMC5119943

[DEV205312C61] Sur, A., Wang, Y., Capar, P., Margolin, G., Prochaska, M. K. and Farrell, J. A. (2023). Single-cell analysis of shared signatures and transcriptional diversity during zebrafish development. *Dev. Cell* 58, 3028-3047.e12. 10.1016/j.devcel.2023.11.001 37995681 PMC11181902

[DEV205312C62] Sztal, T., Berger, S., Currie, P. D. and Hall, T. E. (2011). Characterization of the laminin gene family and evolution in zebrafish. *Dev. Dyn.* 240, 422-431. 10.1002/dvdy.22537 21246659

[DEV205312C63] Thisse, B., Heyer, V., Lux, A., Alunni, V., Degrave, A., Seiliez, I., Kirchner, J., Parkhill, J.-P. and Thisse, C. (2004). Spatial and temporal expression of the zebrafish genome by large-scale in situ hybridization screening. *Methods Cell Biol.* 77, 505-519. 10.1016/s0091-679x(04)77027-2 15602929

[DEV205312C64] Thisse, B., Wright, G. J. and Thisse, C. (2008). Embryonic and larval expression patterns from a large scale screening for novel low affinity extracellular protein interactions. ZFIN Direct Data Submission. (http://zfin.org)

[DEV205312C65] Truong, H. and Danen, E. H. J. (2009). Integrin switching modulates adhesion dynamics and cell migration. *Cell Adhes. Migr.* 3, 179-181. 10.4161/cam.3.2.8036 PMC2679881 19287215

[DEV205312C66] Valentin, G., Haas, P. and Gilmour, D. (2007). The chemokine SDF1a coordinates tissue migration through the spatially restricted activation of Cxcr7 and Cxcr4b. *Curr. Biol.* 17, 1026-1031. 10.1016/j.cub.2007.05.020 17570670

[DEV205312C67] Vaughan, R. B. and Trinkaus, J. P. (1966). Movements of epithelial cell sheets *in vitro*. *J. Cell Sci.* 1, 407-413. 10.1242/jcs.1.4.407 5956715

[DEV205312C68] Venero Galanternik, M., Kramer, K. L. and Piotrowski, T. (2015). Heparan sulfate proteoglycans regulate Fgf signaling and cell polarity during collective cell migration. *Cell Rep.* 10, 414-428. 10.1016/j.celrep.2014.12.043 25600875 PMC4531098

[DEV205312C69] Walma, D. A. C. and Yamada, K. M. (2020). The extracellular matrix in development. *Development* 147, dev175596. 10.1242/dev.175596 32467294 PMC7272360

[DEV205312C70] Watanabe, K. and Tachibana, T. (1973). Transmission and scanning electron microscopic study of adepidermal granules of teleosts and amphibia. *Z. Zellforsch Mikrosk. Anat.* 142, 163-170. 10.1007/BF00307030 4746513

[DEV205312C71] Webb, A. E., Sanderford, J., Frank, D., Talbot, W. S., Driever, W. and Kimelman, D. (2007). Laminin α5 is essential for the formation of the zebrafish fins. *Dev. Biol.* 311, 369-382. 10.1016/j.ydbio.2007.08.034 17919534

[DEV205312C72] Weiner, O. D., Servant, G., Welch, M. D., Mitchison, T. J., Sedat, J. W. and Bourne, H. R. (1999). Spatial control of actin polymerization during neutrophil chemotaxis. *Nat. Cell Biol.* 1, 75-81. 10.1038/10042 10559877 PMC2828058

[DEV205312C73] Weiss, P. and Ferris, W. (1954). Electron-microscopic study of the texture of the basement membrane of larval amphibian skin. *Proc. Natl. Acad. Sci. USA* 40, 528-540. 10.1073/pnas.40.6.528 16589519 PMC534083

[DEV205312C74] Yamada, K. M. and Sixt, M. (2019). Mechanisms of 3D cell migration. *Nat. Rev. Mol. Cell Biol.* 20, 738-752. 10.1038/s41580-019-0172-9 31582855

[DEV205312C75] Yamaguchi, N., Zhang, Z., Schneider, T., Wang, B., Panozzo, D. and Knaut, H. (2022). Rear traction forces drive adherent tissue migration in vivo. *Nat. Cell Biol.* 24, 194-204. 10.1038/s41556-022-00844-9 35165417 PMC8868490

[DEV205312C76] Yurchenco, P. D., Quan, Y., Colognato, H., Mathus, T., Harrison, D., Yamada, Y. and O'Rear, J. J. (1997). The α chain of laminin-1 is independently secreted and drives secretion of its β- and γ-chain partners. *Proc. Natl. Acad. Sci. USA* 94, 10189-10194. 10.1073/pnas.94.19.10189 9294185 PMC23337

