# Supplementary material for: Laminin α5 and integrins α3 and α6 coordinately regulate collective cell migration in vivo
Source: Development. 2026 Jun 11;153(16):dev205312. doi: 10.1242/dev.205312 (PMC13286361; doi:10.1242/dev.205312)
Supplement: Supplementary information [file develop-153-205312-s1.pdf]

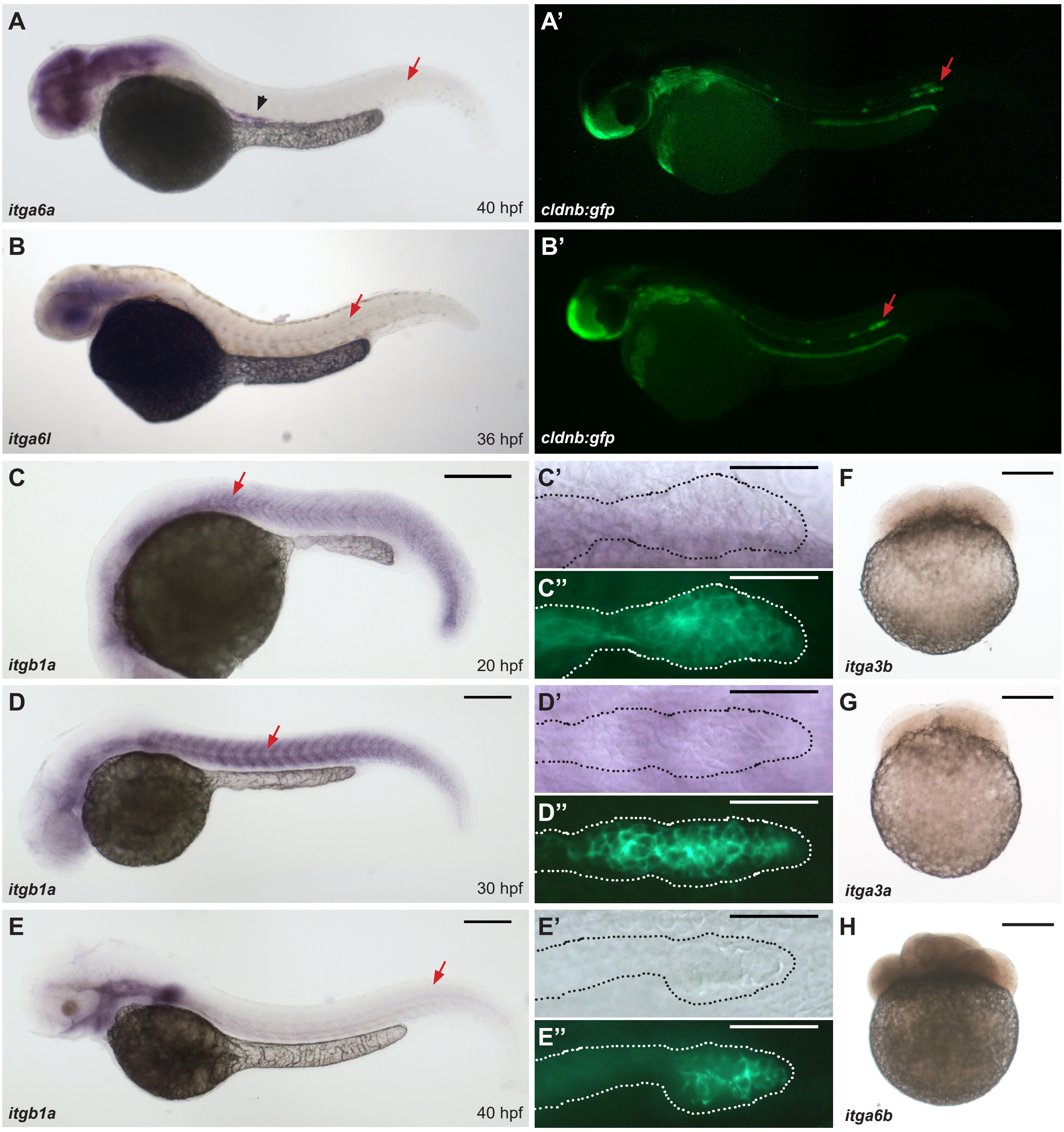

Fig. S1. Expression of integrin paralogues by ISH

(A-E'') *itga6a*, *itga6l* and *itgb1a* are not expressed in the migrating pLLP at the indicated stages. (F-H) *itga3a*, *itga3b* and *itga6b* mRNA are not provided maternally.

Scale bars: 200µm (A-E), 50µm (C'-E'', F-H).

Development • Supplementary information

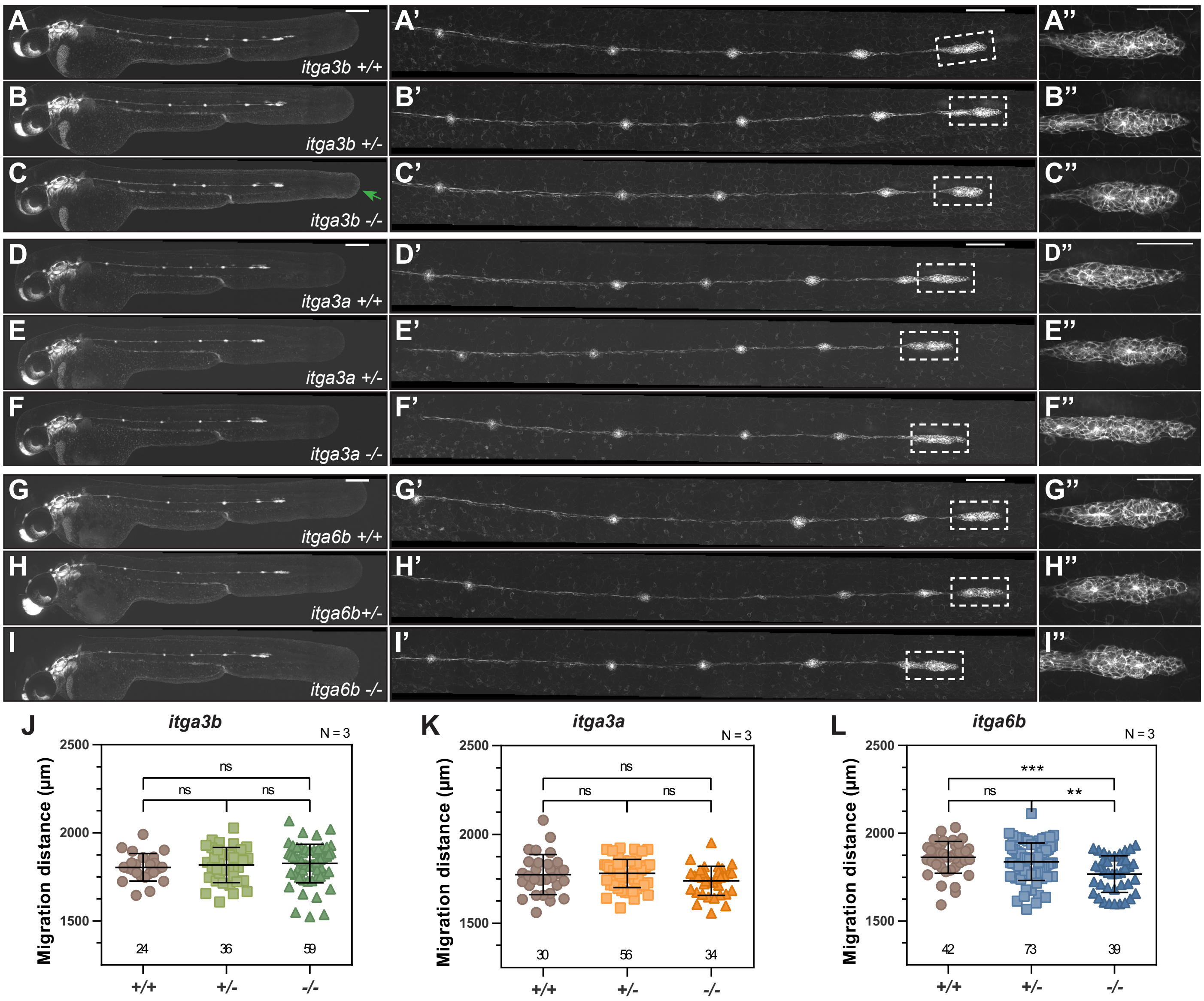

Fig. S2. *itga6b*, but not *itga3a* or *itga3b*, is required for proper pLLP migration. (A-I″) *cldnb:GFP* embryos at 40 hpf of wild-type, heterozygous and homozygous *itga3b* (A-C″), *itga3a* (D-F″) and *itga6b* (G-I″) mutants, including embryo overviews (A-I), higher magnifications of the pLL (A′-I′), and a close-ups on the pLLP (A″-I″). A green arrow in (C) indicates the "*badfin*" phenotype of *itga3b* mutants. (J-L) Quantification of the distance migrated by the pLLP at 40 hpf. Statistics: One-way ANOVA with multiple comparisons; ns=non-significant; **p<0.01; ***p<0.001. Sample size (n) are indicated above x-axes; N=number of biological replicates. Scale bars: 200 µm (A-I), 100 µm (A′-I′) and 50 µm (A″-I″).

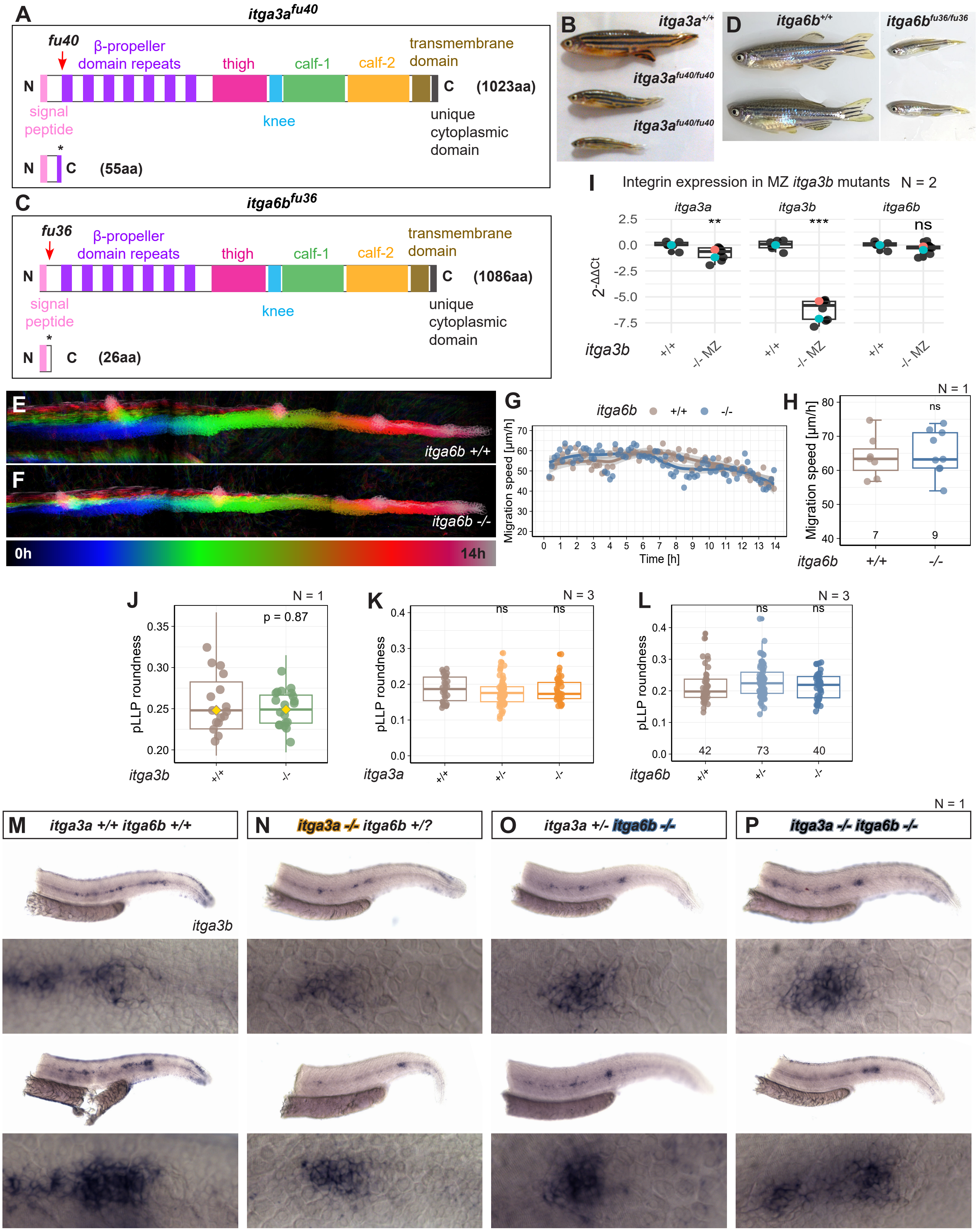

Fig. S3. Integrin mutant generation and qPCR analysis

(A, C) Schematic of wild-type and mutant *itga3a*^{fu40} and *itga6b*^{fu36} proteins, respectively

(B, D) Rare escapers of *itga3a* and *itga6b* homozygous mutants with severe dwarfism.

(E-F) 14-hour TLs of wild-type and *itga6b* mutant pLLPs displayed as temporally color-coded MIPs.

(G-H) Quantification of instantaneous and mean speeds shows no difference in pLLP migration speed in *itga6b* mutants. (I) qRT-PCR expression analysis of *itga3a*, *itga3b* and *itga6b* in maternal zygotic (MZ) *itga3b* mutants (J-L) Quantified pLLP roundness of *itga3b* (J), *itga3a* (K) and *itga6b* mutants (L).

(M-P) Unchanged *itga3b* expression pattern in *itga3a;6b* mutants and siblings; ISH performed on fins pre-genotyped using the embryo heads.

Statistics: two-tailed Wilcoxon rank-sum test (H-L); ns = non-significant; **p < 0.01; ***p < 0.001. Sample sizes (n) and number of biological replicates (N) are indicated.

Development • Supplementary information

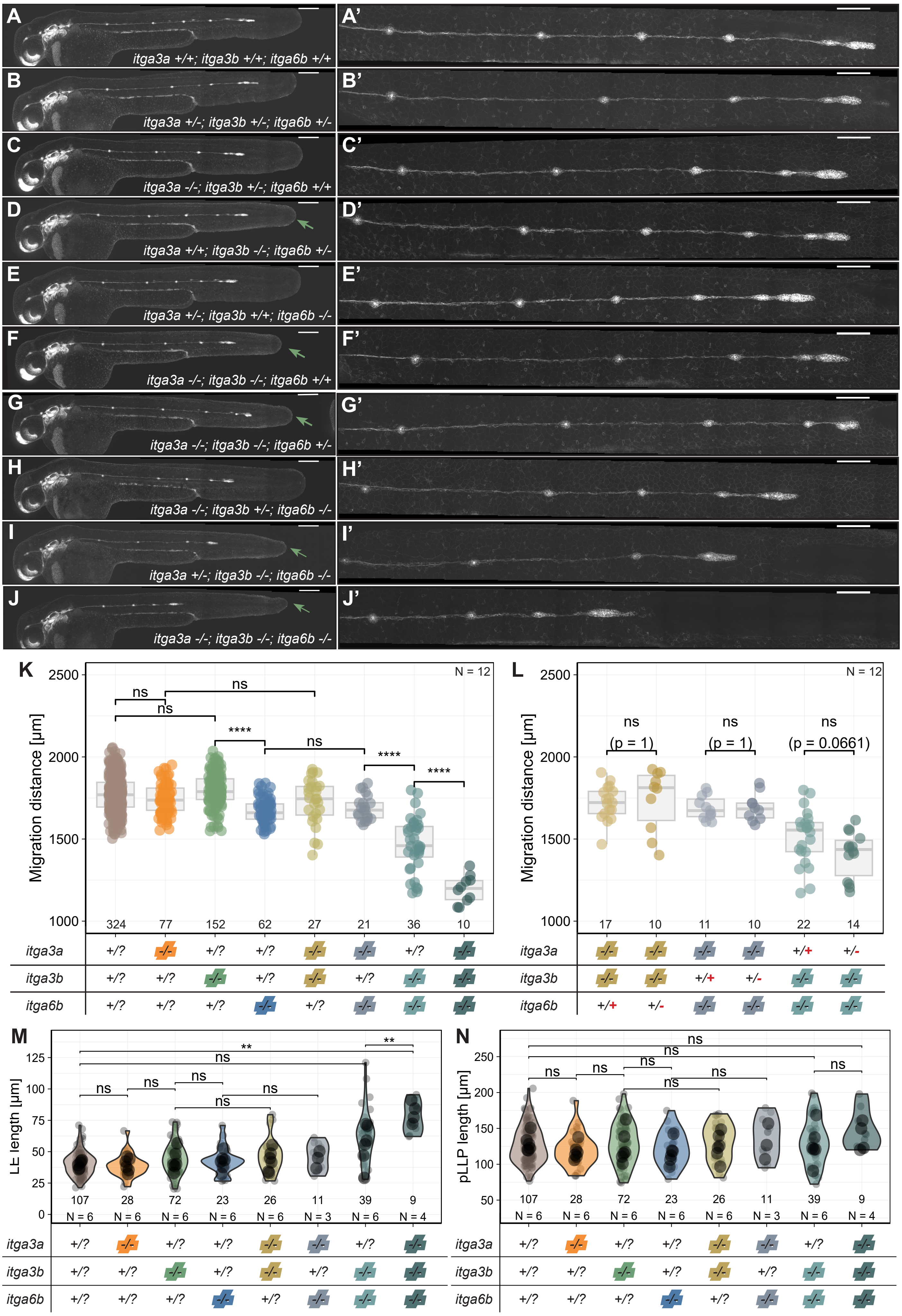

Development • Supplementary information

Fig. S4. Integrin mutant analysis

(A-J‘) Full panel of triple integrin mutant combinations from Figure 2, including embryo overviews (A-J) and higher ma-gnifications of the pLL (A’-J’).

(K-L) Quantification of pLLP migration distance at 40 hpf with +/? genotypes (K) and separated to justify +/? pooling of +/+ and +/- in +/? (L).

(M-N) Leading region and total pLLP length of quantified ratio in main Figure 3. Number of individuals with correspon-ding number of biological replicates indicated above x-axes.

Statistics: Selected pairwise comparisons of estimated marginal means from a linear mixed-effects model (N as random effect), with Tukey correction for multiple comparisons (K, L) and pairwise Wilcoxon rank-sum tests on per-experiment means (M-N); ns = non-significant; ****p < 0.0001.

Sample sizes (n) are indicated above x-axes. N = number of biological replicates .

Scale bars: 200µm (A-J), 100µm (A’-J’).

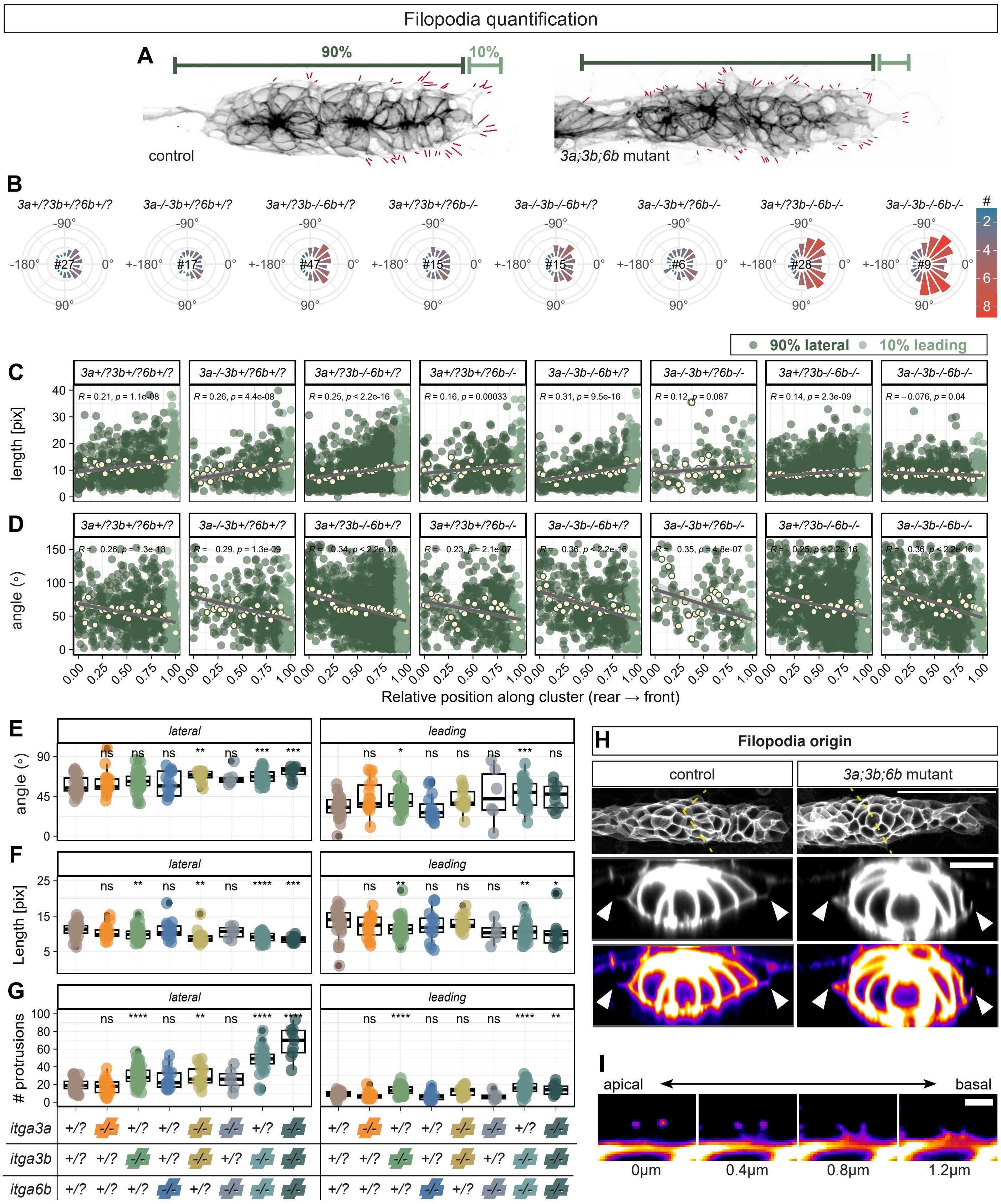

Development • Supplementary information

Fig. S5. Filopodia quantification of double *itga3b;6b* and triple *itga3a;3b;6b* integrin mutants.
(A) Schematic displays non-biased classification of filopodia into leading 10% (light green) and lateral 90% (dark green) (B) Full panel related to Figure 3, polar plots displaying mean filopodia number per angular bin with 0° being direction of migration. Number of individuals indicated in the middle.
(C-D) Filopodia length (C) and absolute angle (D) plotted by relative x-position from rear to front. Color code represents leading/lateral classification as shown in (A).
(E-G) Boxplots display mean filopodia angle (E), length (F) and number (G) grouped by leading/lateral.
(H) Digital X-Z sections as indicated with the yellow dashed lines on the upper panels showing the apico-basal origin of filopodia (white arrowheads).
(I) Consecutives z-planes through two filopodia from apical to basal, as indicated, showing that filopodia are often pointing apically towards the overlying skin.
Statistics: two-tailed Wilcoxon rank-sum tests. ns = non-significant; *$p < 0.05$; **$p < 0.01$; ***$p < 0.001$; ****$p < 0.0001$.
Scale bars: 50µm (H overview), 10µm (H digital section), 2µm (I).

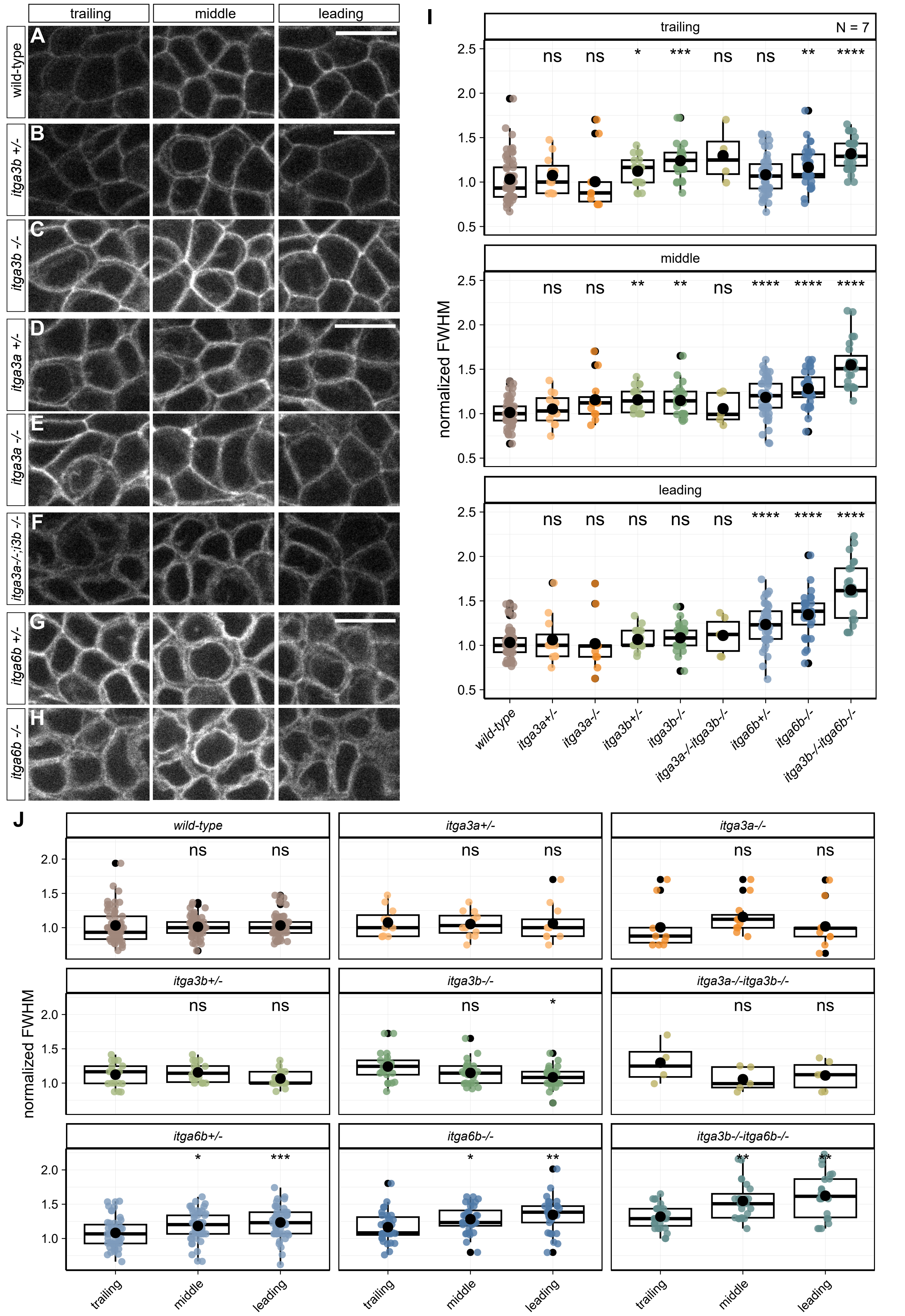

Fig. S6. Itgb1b-sfGFP regional delocalization upon loss of *itga3b* and *itga6b*

(A-H) Itgb1b-sfGFP signal in wild-type (A), *itga3b* (B-C), *itga3a* (D-E), *itga3a;3b* (F) and *itga6b* (G-H) mutants. (I-J) Full FWHM quantification grouped by region (I) and genotype (J) including the results shown in Figure 3. Statistics: two-tailed Wilcoxon rank-sum tests. ns = non-significant; *p < 0.05; **p < 0.01; ***p < 0.001; ****p < 0.0001. Scale bars: 10µm.

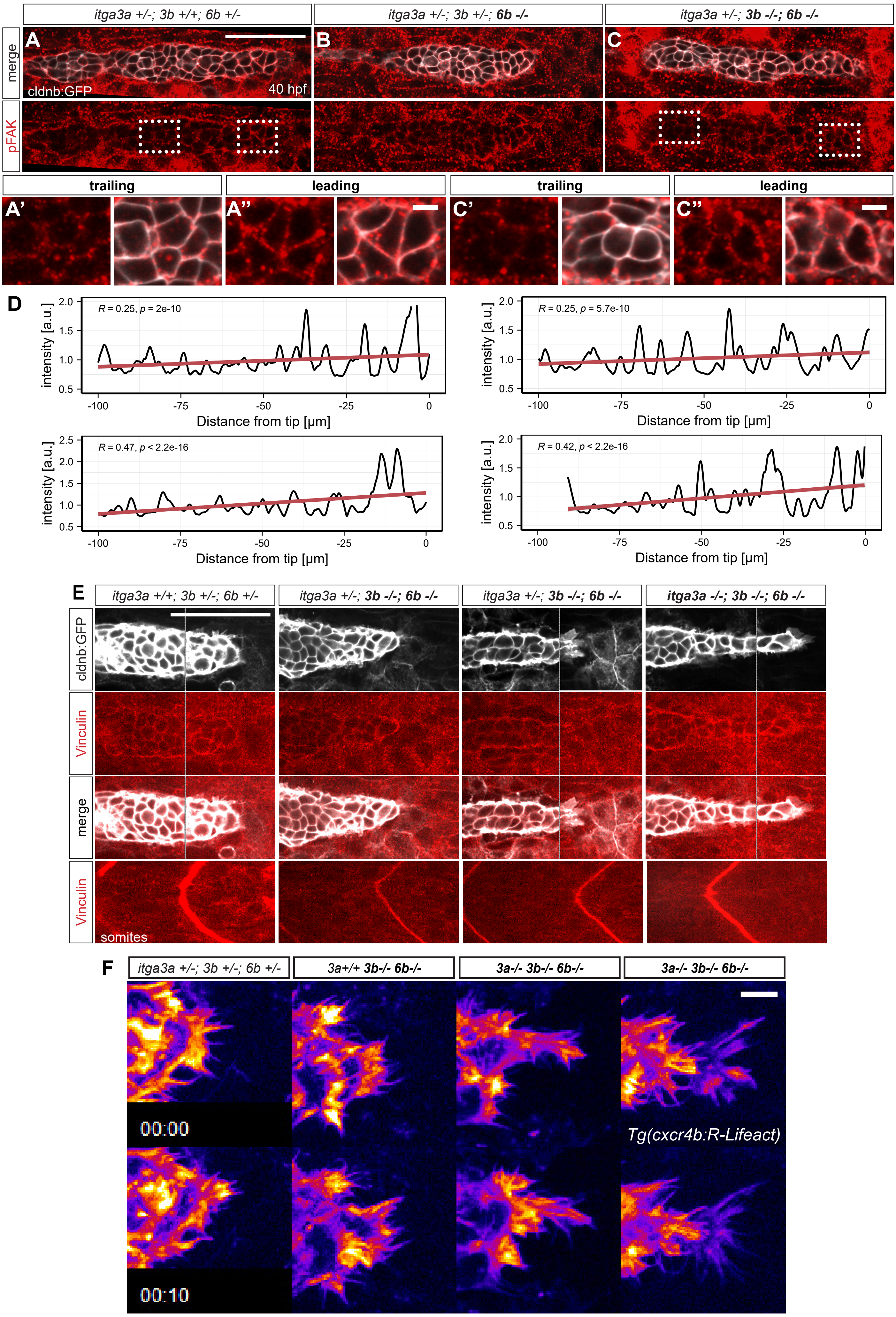

Development • Supplementary information

Fig. S7. Focal adhesion markers and dynamic protrusions in double and triple integrin mutants

(A-C) pFAK immunostaining in control (A), *itga6b* (B) and double *itga3b;6b* mutants (C) displays a leading-trailing gradient which is preserved in the mutants (A'-C''). (D) Representative intensity profiles depict the increase in pFAK levels towards the tip of the primordium.

(E) Confocal Z-planes of Vinculin immunostaining in control, *itga3b;6b* double and *itga3a;3b;6b* triple mutants displays no significant change in localization. Vinculin levels are decreased in the somites and somite boundaries in the mutants. When two different Z-planes are shown for the leading/middle regions, this is indicated by a grey line.

(F) Timelapse of *cxcr4b:R-Lifeact* depicts dynamic actin-based protrusions in *itga3b;6b* double and *itga3a;3b;6b* triple mutants. Shown are two snapshots from Video S2.

Scale bars: 50µm for overview (A, E), 5µm for close-ups (A'-C'', F).

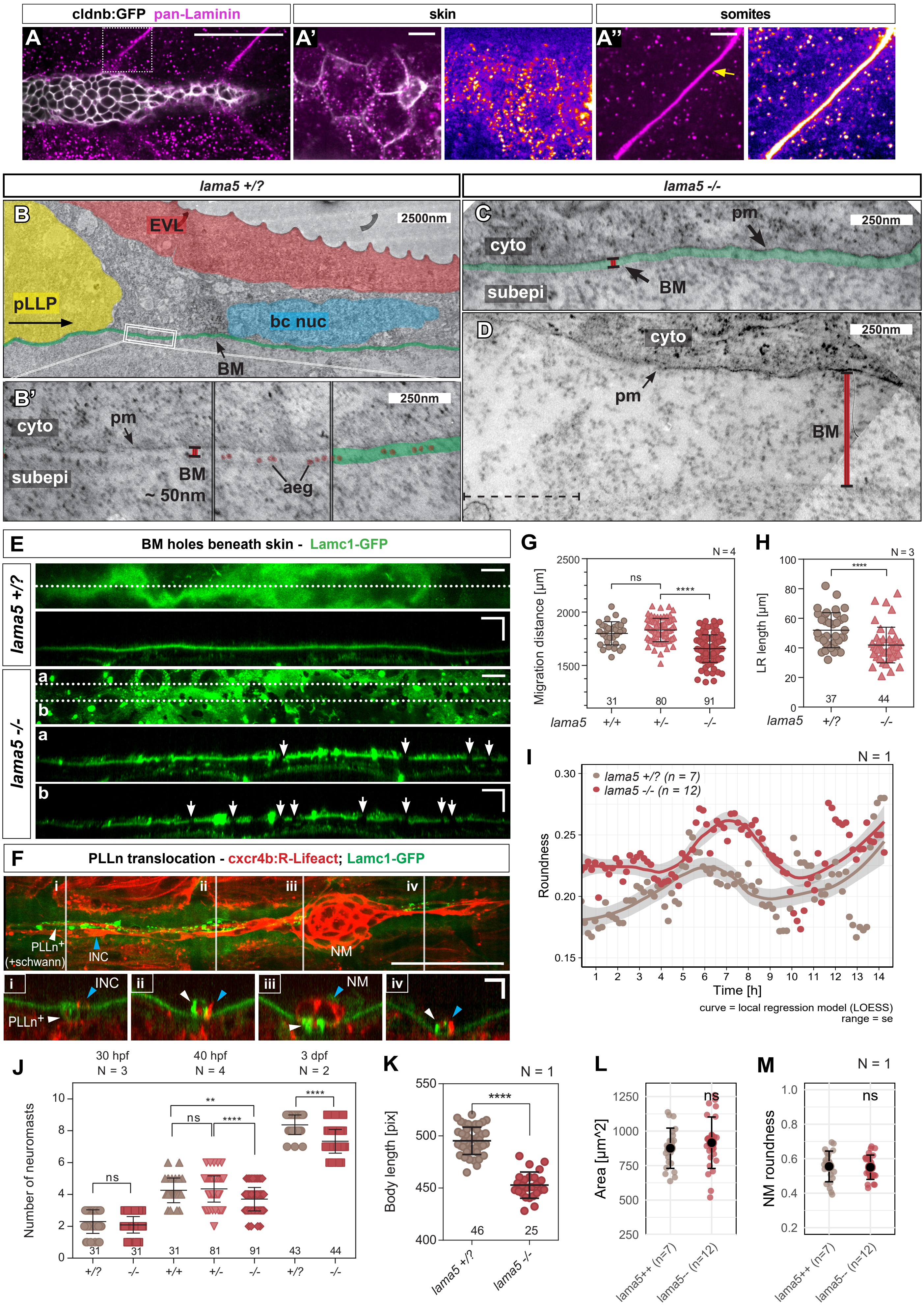

Development • Supplementary information

Fig. S8. *lama5* mutant analysis

(A-A'') Single z-planes of pan-laminin staining related to Figure 4 showing unspecific signal in skin cells (A') and strong signal at somite boundaries (A'').

(B-D) TEM in 32hpf sibling (B) and *lama5*$^{-/-}$ (C,D) embryos. (B) Shown is the pLLP leading region (yellow) with the underlying epidermal BM (green). (B') Close-up as indicated in (A) showing the BM spanning ~50nm and containing first adepidermal granules (aeg, pseudo-colored orange in B' middle and right panels). (C) In the BM of *lama5* mutants, adepidermal granules are missing from intact BM regions, where the width is comparable to control siblings (B'). (D) In the proximity of presumptive BM holes (dashed line), the BM is bloated.

(E) Holes in the BM of *lama5* mutants labelled by Lamc1-GFP found in skin regions unrelated to the pLLP migration path, related to Figure 4. Digital sections as indicated by white dashed lines.

(F) pLL nerve (PLLn) translocation in wild-type embryo at 40hpf. Digital sections as indicated by white dashed lines (i-iv). PLLn labelled by cxcr4b:R-Lifeact, surrounding schwann cells express Lamc1-GFP. Most anterior (i), the PLLn+ is found below/medially to the BM while more posterior it resides on top/laterally (ii, iv). (iii) Underneath the neuromast, the PLLn+ is found in a trans-state, suggesting mechanical pressure influences PLLn translocation.

(G) Quantification of the distance migrated by the pLLP in *lama5* mutants including heterozygous individuals at 40hpf.

(H) Quantification of the pLLP leading region (LR) in *lama5* mutants.

(I) Quantification of pLLP roundness over time in control and *lama5* mutants, related to Figure 5.

(J) Number of neuromasts in *lama5* mutants vs. siblings at indicated stages.

(K) Body length of *lama5* mutants at 40hpf.

(L-M) Neuromast (NM) size and roundness in control vs. *lama5* mutants from TL data (Figure 5).

Statistics: One-way ANOVA (H, K) with multiple comparisons (G,J) and two-tailed Wilcoxon rank-sum test (L-M); ns = non-significant; ****p < 0.0001.

Scale bars: 50µm (A, F overview), 10µm (E), 5µm (A'-A'', Fi-iv).

cyto = cytoplasm, pm = plasma membrane, BM = basement membrane, aeg = adepidermal granule, EVL = enveloping layer, bc nuc = epidermal basal cell nucleus, subepi = subepidermal space.

Development • Supplementary information

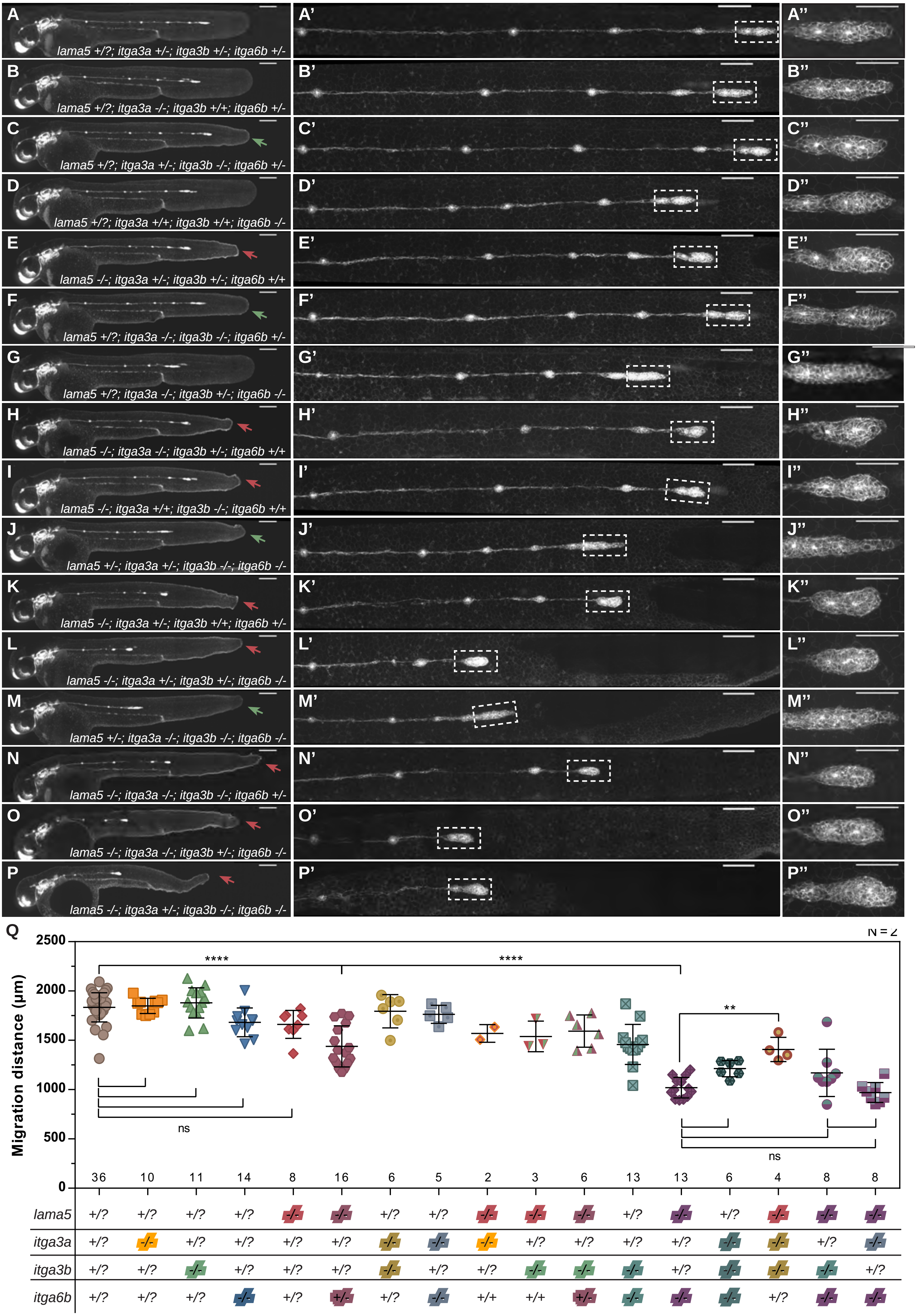

A
lama5 +/?; itga3a +/-; itga3b +/-; itga6b +/-
A'
A''
B
lama5 +/?; itga3a -/-; itga3b +/+; itga6b +/-
B'
B''
C
lama5 +/?; itga3a +/-; itga3b -/-; itga6b +/-
C'
C''
D
lama5 +/?; itga3a +/+; itga3b +/+; itga6b -/-
D'
D''
E
lama5 -/-; itga3a +/-; itga3b +/-; itga6b +/+
E'
E''
F
lama5 +/?; itga3a -/-; itga3b -/-; itga6b +/-
F'
F''
G
lama5 +/?; itga3a -/-; itga3b +/-; itga6b -/-
G'
G''
H
lama5 -/-; itga3a -/-; itga3b +/-; itga6b +/+
H'
H''
I
lama5 -/-; itga3a +/+; itga3b -/-; itga6b +/+
I'
I''
J
lama5 +/-; itga3a +/-; itga3b -/-; itga6b -/-
J'
J''
K
lama5 -/-; itga3a +/-; itga3b +/+; itga6b +/-
K'
K''
L
lama5 -/-; itga3a +/-; itga3b +/-; itga6b -/-
L'
L''
M
lama5 +/-; itga3a -/-; itga3b -/-; itga6b -/-
M'
M''
N
lama5 -/-; itga3a -/-; itga3b -/-; itga6b +/-
N'
N''
O
lama5 -/-; itga3a -/-; itga3b +/-; itga6b -/-
O'
O''
P
lama5 -/-; itga3a +/-; itga3b -/-; itga6b -/-
P'
P''
Q
N = 2
Migration distance (µm)
2500
2000
1500
1000
500
0
****
****
**
ns
ns
36 10 11 14 8 16 6 5 2 3 6 13 13 6 4 8 8
lama5 +/? +/? +/? +/? -/- -/- +/? +/? -/- -/- -/- +/? -/- +/? -/- -/- -/-
itga3a +/? -/- +/? +/? +/? +/? -/- -/- -/- +/? +/? +/? +/? -/- -/- +/? -/-
itga3b +/? +/? -/- +/? +/? +/? -/- +/? +/? -/- -/- -/- +/? -/- -/- -/- +/?
itga6b +/? +/? +/? -/- +/? +/- +/? -/- +/+ +/+ +/- -/- -/- -/- +/? -/- -/-

Fig. S9. Analysis of laminin-integrin mutant combinations.

(A-P‘‘) Full panel of lama5-integrin mutant combinations from Figure 6, including embryo overviews (A-P), higher mag-nifications of the pLL (A'-P') and close-ups on the pLLP (A''-P'').

Green and red arrows indicate “badfin” and “fransen” phenotypes, respectively.

(Q) Quantification of the distance migrated by the pLLP at 40hpf.

Statistics: One-way ANOVA with multiple comparisons; ns = non-significant; $*p < 0.05$; $****p < 0.0001$. Sample sizes (n) are indicated above x-axes. N = number of biological replicates.

Scale bars: 200µm (A-P), 100µm (A'-P') and 50µm (A''-P'').

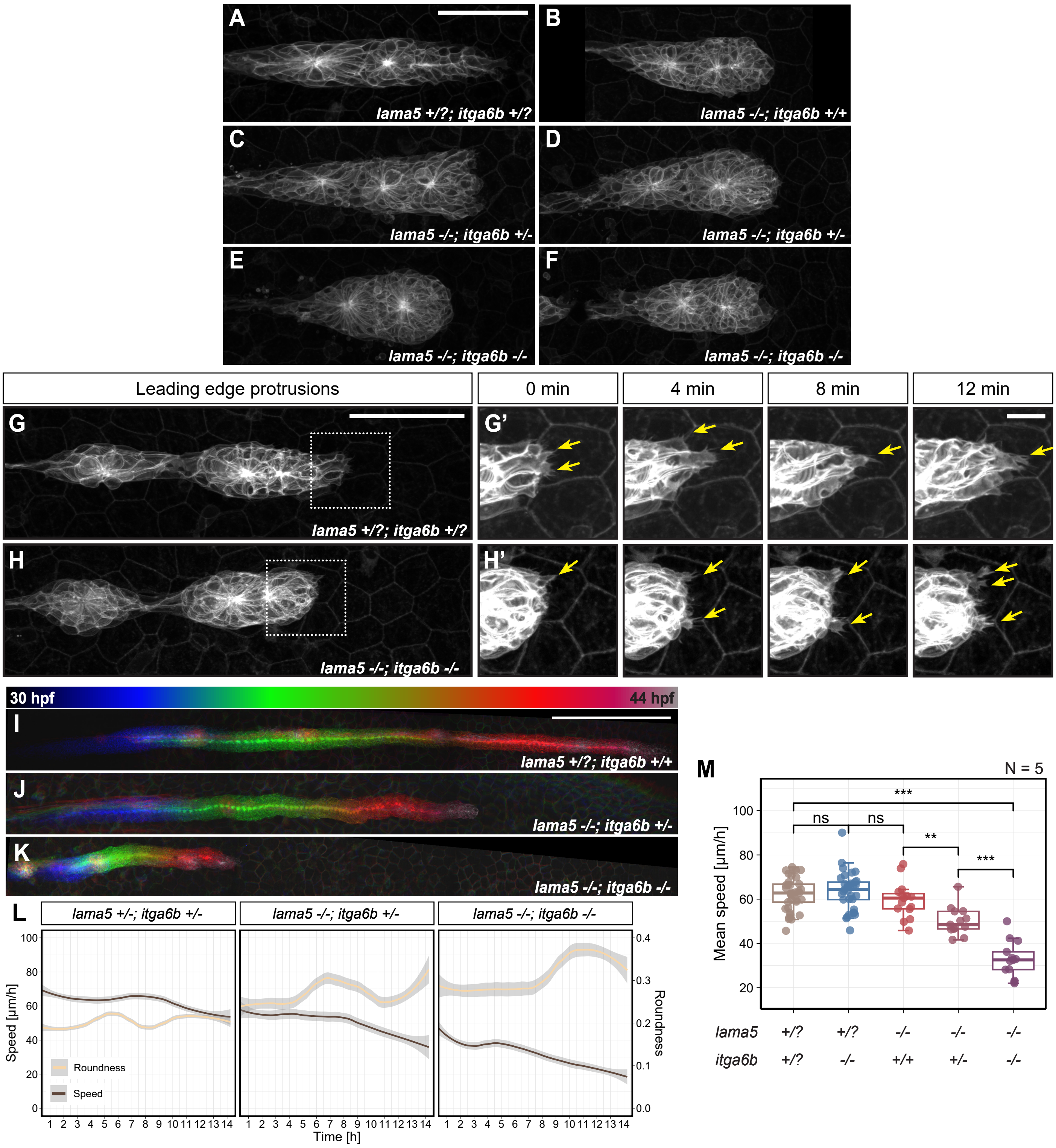

Fig. S10. *lama5;itga6b* mutants

(A-F) Live imaging of pLLPs of representative sibling control (A), *lama5* single mutant (B), *lama5-/-;itga6b+/-* (C-D) and *lama5-/-;itga6b-/-* double mutants (E-F).

(G-H') Time-lapse imaging of dynamic leading edge protrusions in control (G-G') and *lama5;itga6b* double mutant pLLPs (H-H').

(I-K) Temporally color-coded maximum-intensity projections of 14-hour time-lapse imaging of sibling control vs. *lama5;itga6b* compound mutants with the indicated genotype. (L) Loess model of instantaneous speed and pLLP roundness over time. (M) Quantification of the mean migration speed from I-L.

Statistics: One-way ANOVA with multiple comparisons; ns = non-significant; $*p < 0.05$; $****p < 0.0001$. Sample sizes (n) are indicated above x-axes. N = number of biological replicates.

Scale bars: 200µm (I-K), 50µm (A,G), 10µm (G').

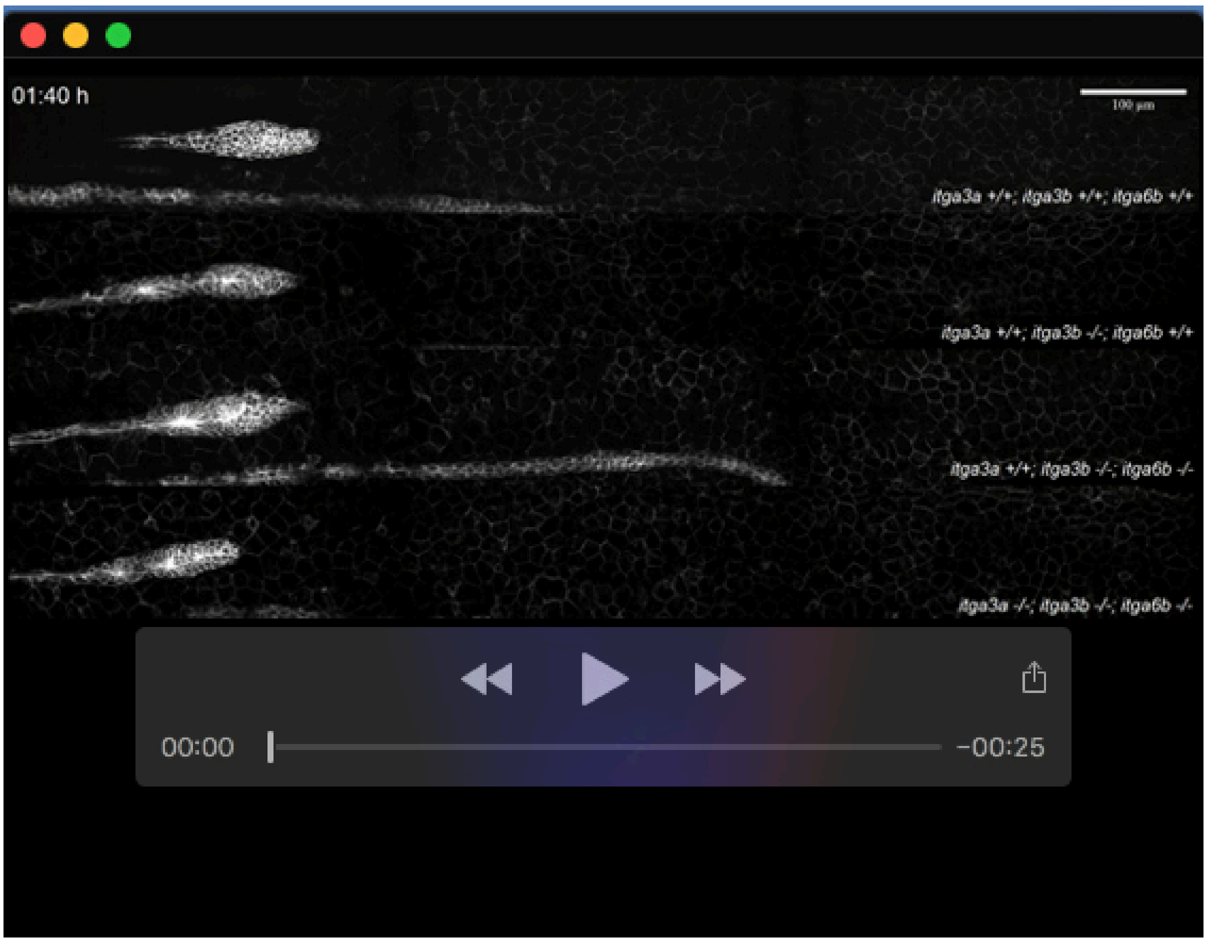

Movie 1. 14h-timelapse of pLLP migration in integrin mutants, related to Figures 2 and S4.
14h-timelapse showing pLLP migration in *cldnb:GFP* sibling (top), *itga3b*$^{-/-}$ (second from top), *itga3b*$^{-/-}$*;itga6b*$^{-/-}$ (second from bottom) double and *itga3a*$^{-/-}$*;itga3b*$^{-/-}$*;itga6b*$^{-/-}$ (bottom) triple integrin mutants. Imaging started around 26hpf.

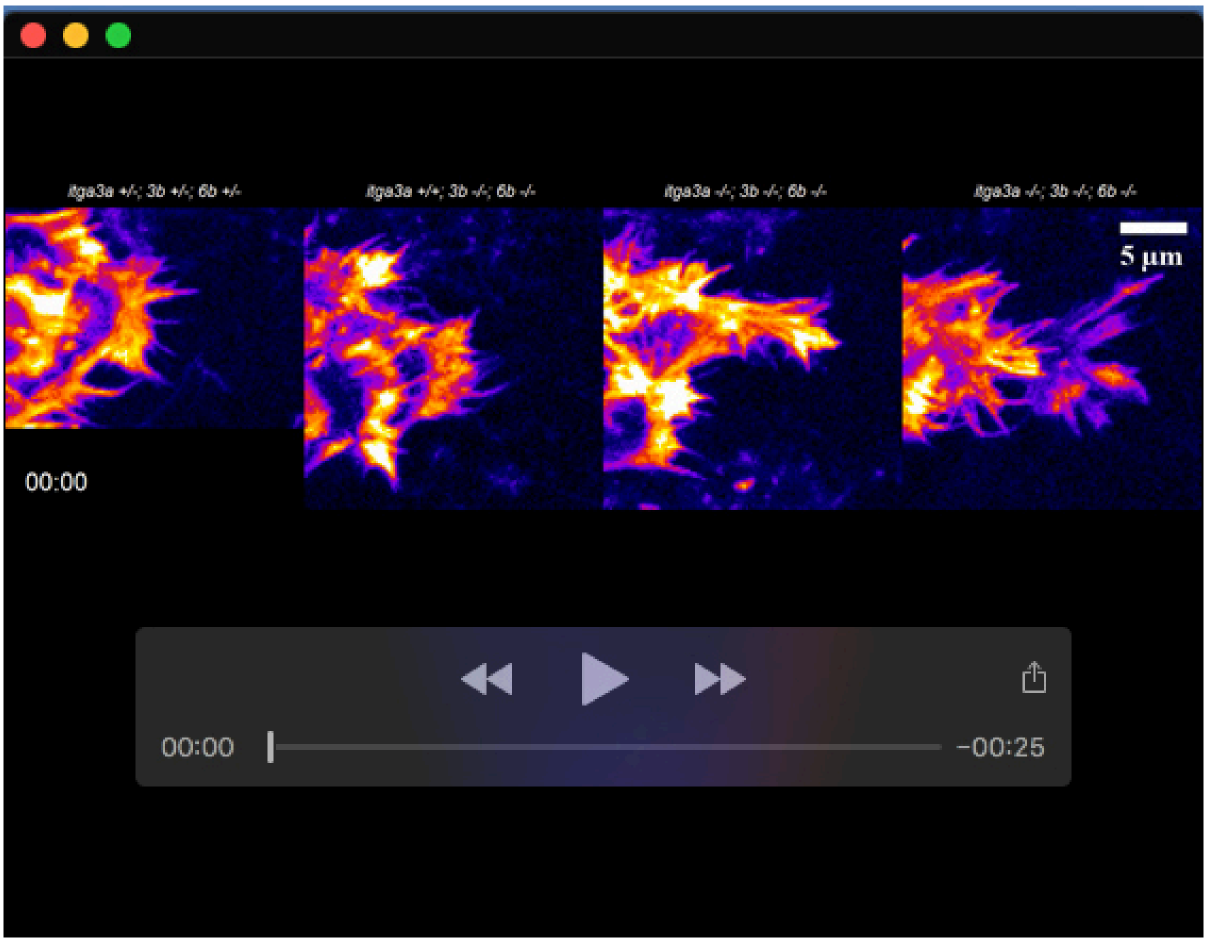

Movie 2. Actin-based protrusions in the leading region of integrin mutant pLLPs, related to Figure S7.
Short timelapse recording of the leading region of control (left), *itga3b;6b* double (middle left) and *itga3a;3b;6b* triple mutant (middle right + right) pLLPs demonstrates active actin transport in filopodial protrusions and the leading edge of trailing cells. Scale bar: 5 µm.

Development • Supplementary information

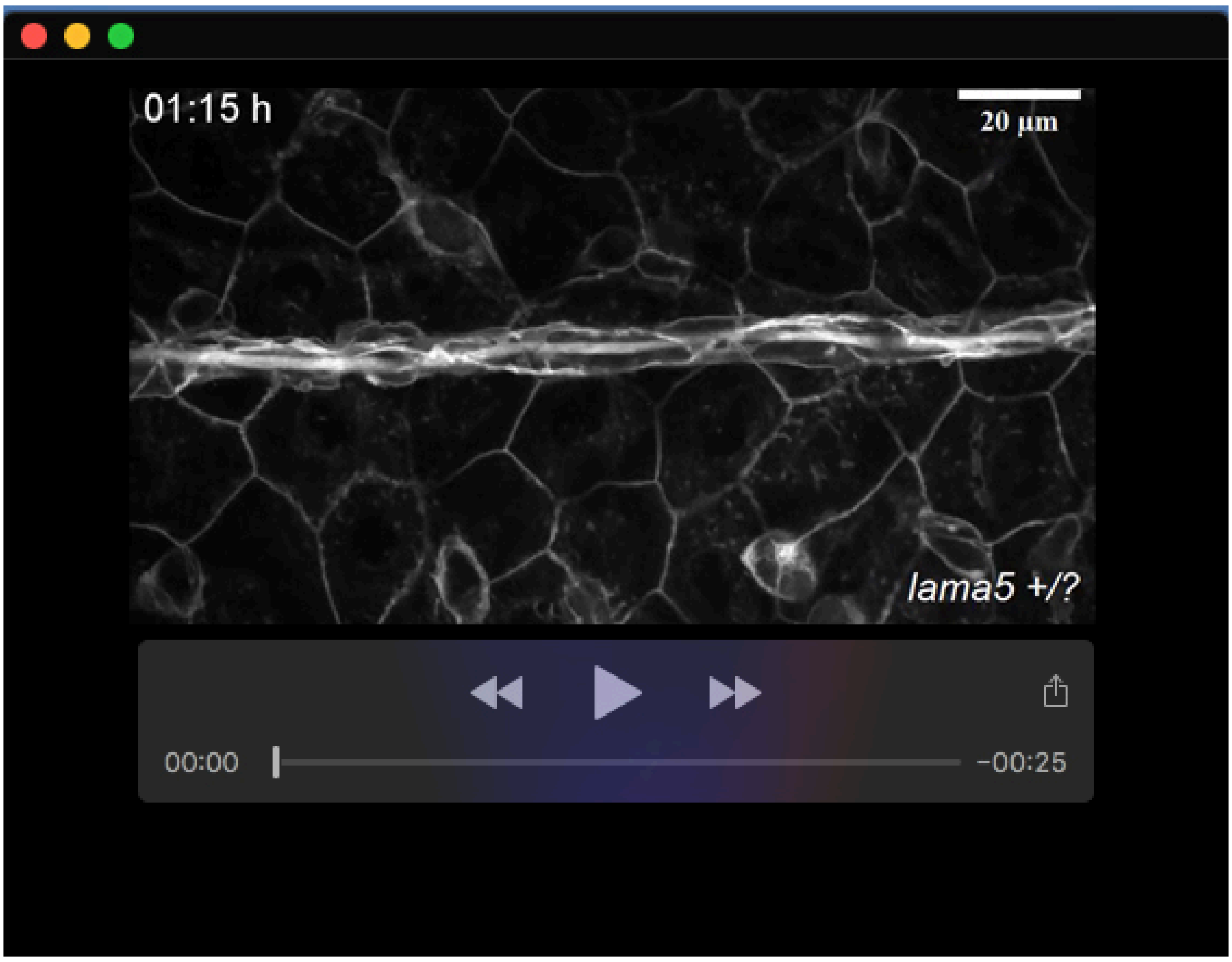

Movie 3. Wild-type interneuromast chain (INC) cell dynamics, related to Figure 5.
Timelapse of the INC in cldnb:GFP *lama5+/?* embryo showing that cells are dynamic but form a coherent chain. Imaging started at 30hpf.

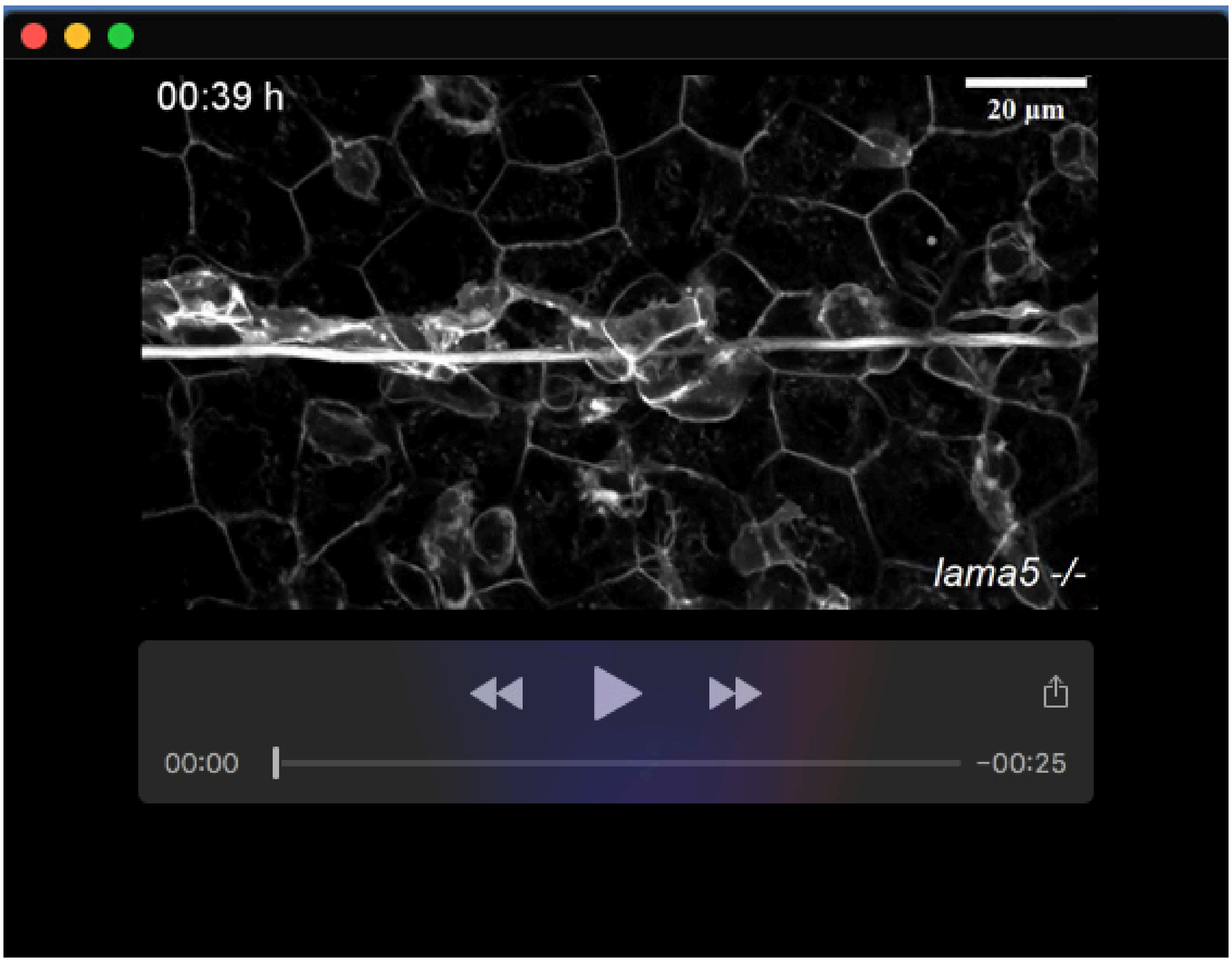

Movie 4. *lama5* mutant INC cell dynamics, related to Figure 5.
Timelapse of the INC in a *cldnb:GFP lama5-/-* embryo showing hyperprotrusive activity of the cells and disruption of the INC. Imaging started at 30hpf.

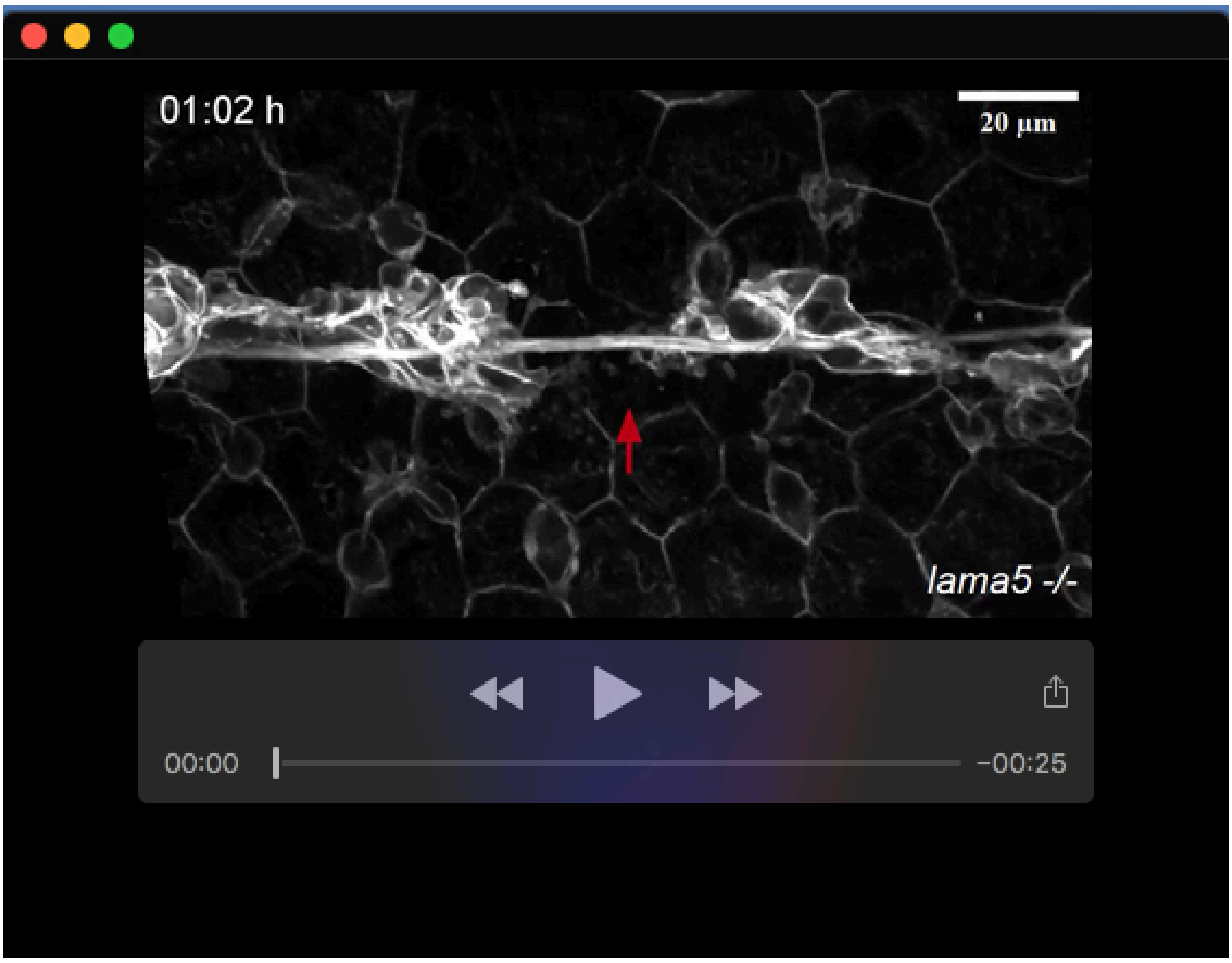

Movie 5. *lama5* mutant INC gap formation, related to Figure 5.
Timelapse of the INC in a *cldnb:GFP lama5-/-* embryo showing the formation of a gap in INC. Imaging started at 30hpf.

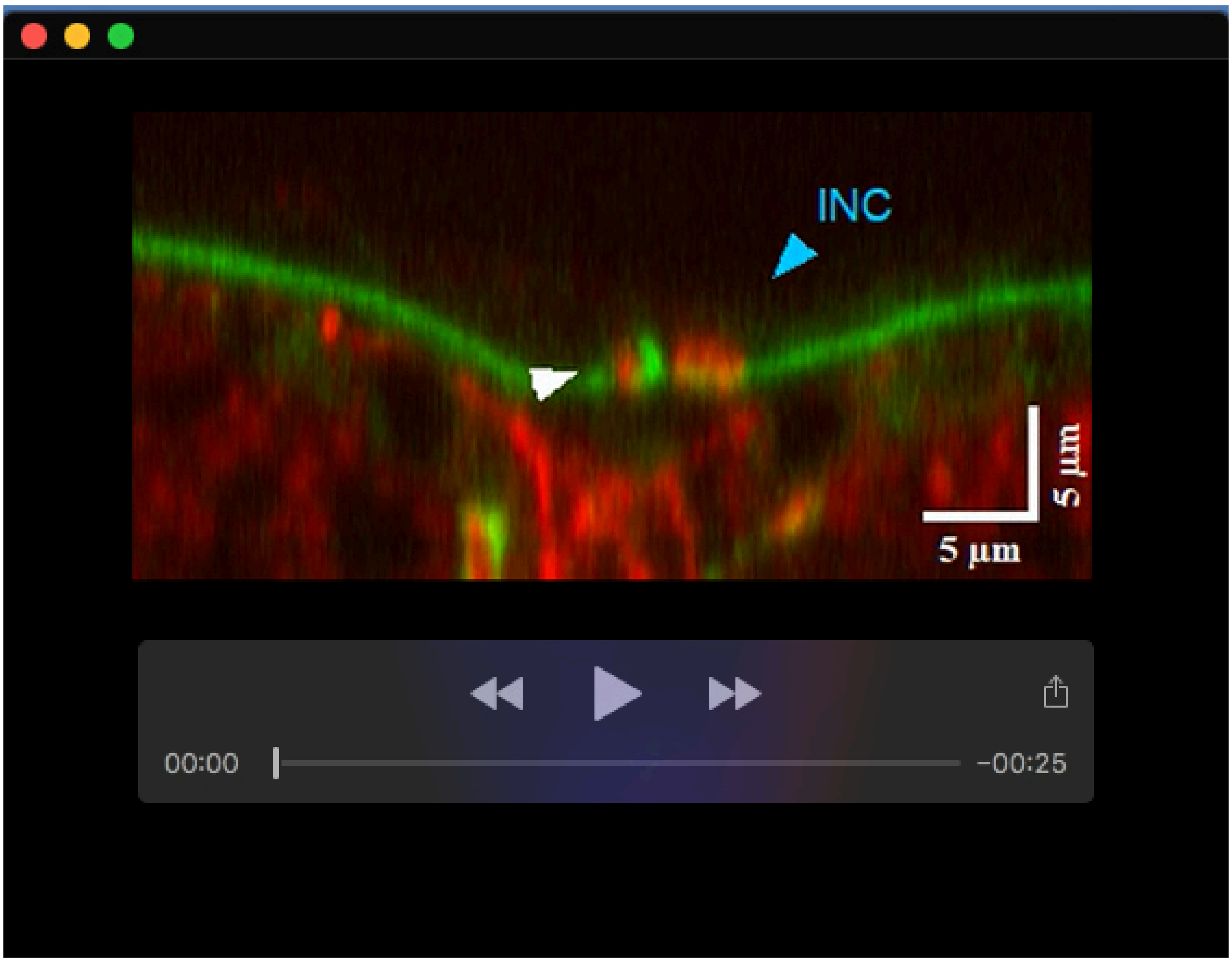

Movie 6. Posterior lateral line nerve (PLLn) translocates from lateral to medial of the epidermal BM. Related to Figure S8.
Digital transverse section of INC/BM/nerve constellation in a wild-type embryo at 40hpf. Movie goes from anterior to posterior, depicting the PLLn (red, *cxcr4b:R-Lifeact*) including surrounding schwann cells (green, *lamc1:lamc1-GFP*) underneath the BM in more anterior region and on top of the BM (lateral) in more posterior regions. Note the trans-BM position at the neuromast (NM) level. Scale bar 5 x 5 µm.

Development • Supplementary information

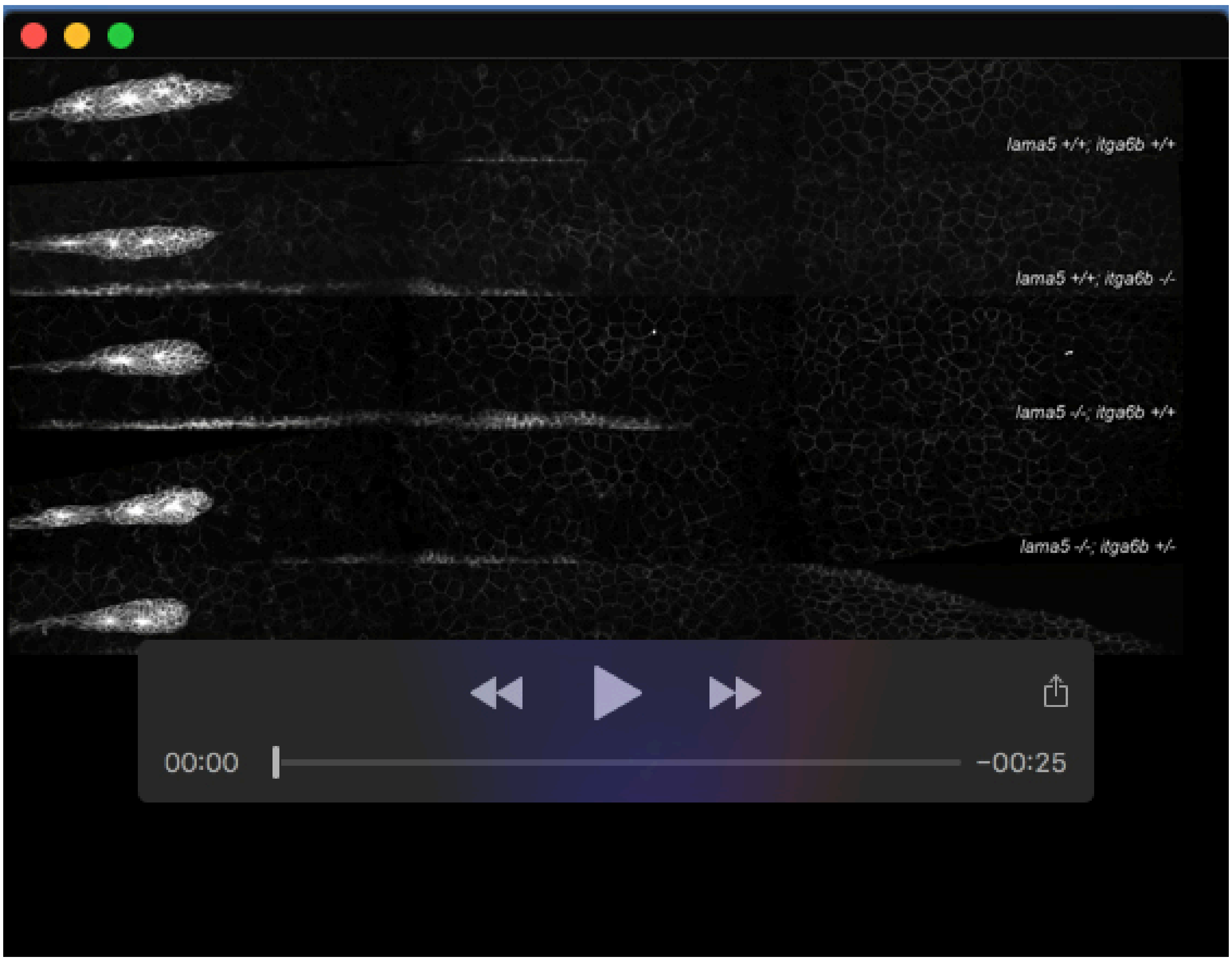

Movie 7. 14h-timelapse of pLLP migration in *lama5;itga6b* mutants, related to Figures 5,6 and S10.
14h-timelapse showing pLLP migration in *cldnb:GFP* sibling (top), *itga6b*$^{-/-}$ (second from top), *lama5*$^{-/-}$ (middle), *lama5*$^{-/-}$ *;itga6b*$^{+/-}$ (second from bottom) and *lama5-/-;itga6b-/-* (bottom) mutants. Imaging started around 26hpf.

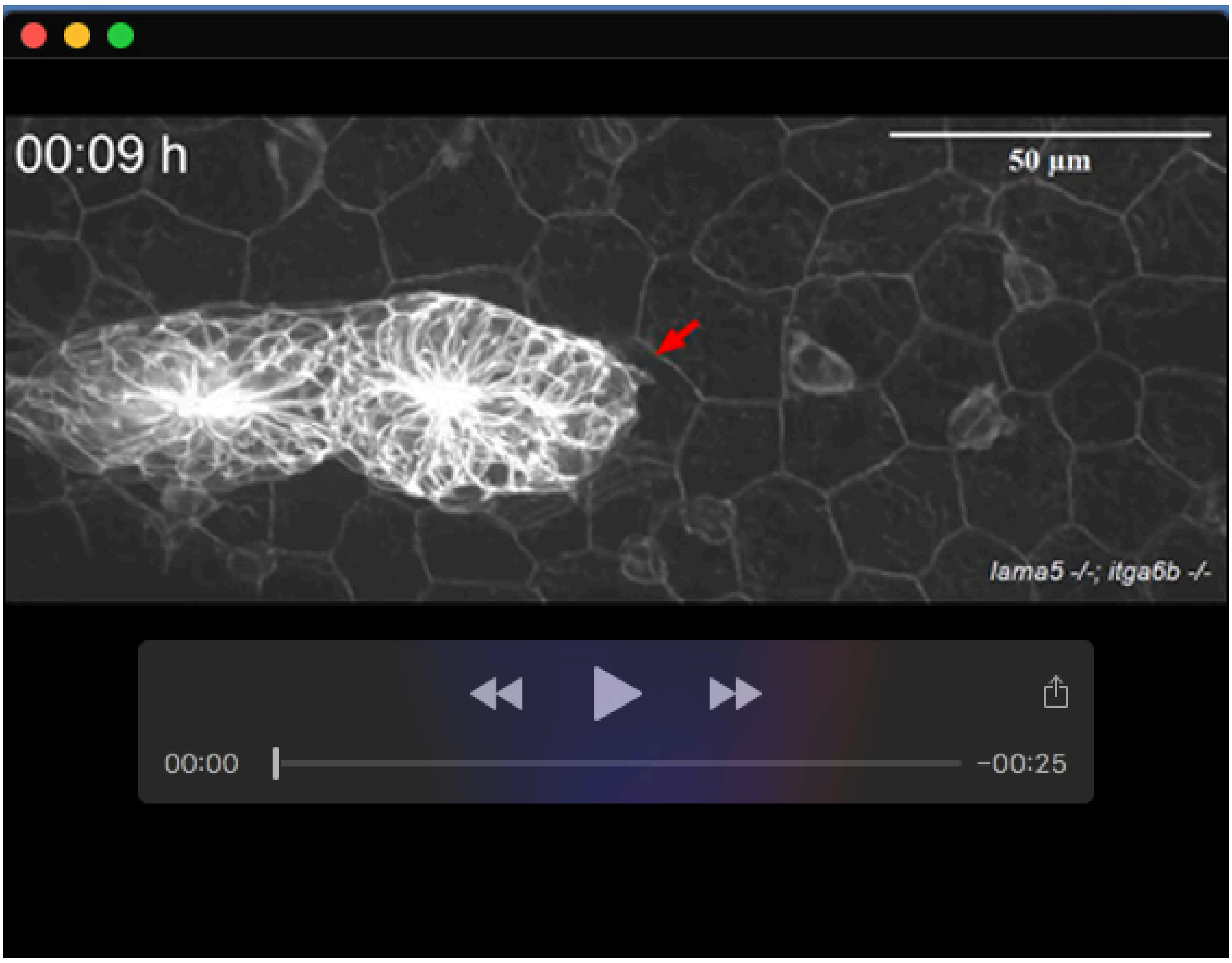

Movie 8. Leading cell protrusions in a stalled *lama5;itga6b* double mutant pLLP, related to Figures 6 and S10.

Development • Supplementary information

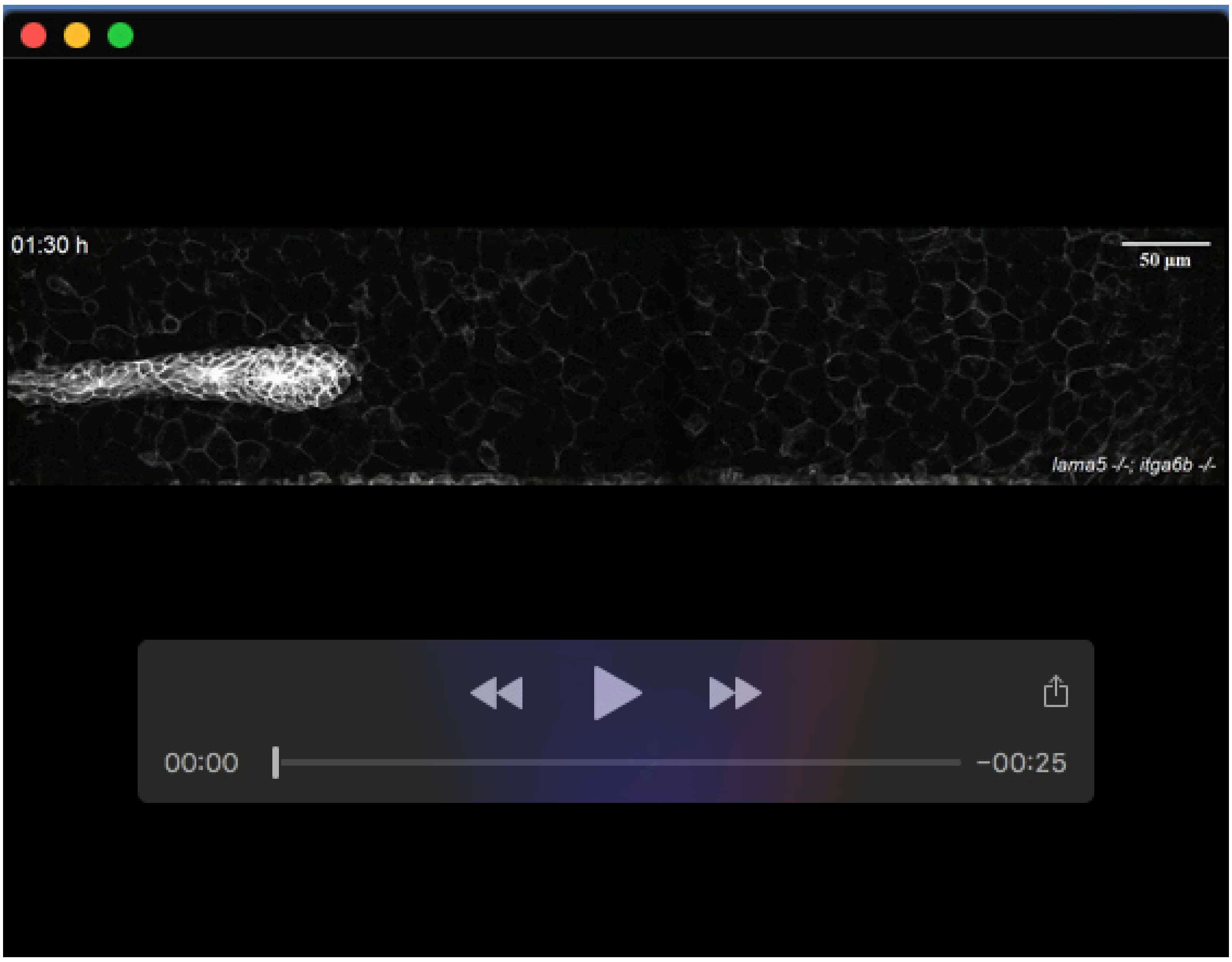

Movie 9. Leading edge splitting in a *lama5;itga6b* double mutant pLLP, related to Figures 6 and S10.

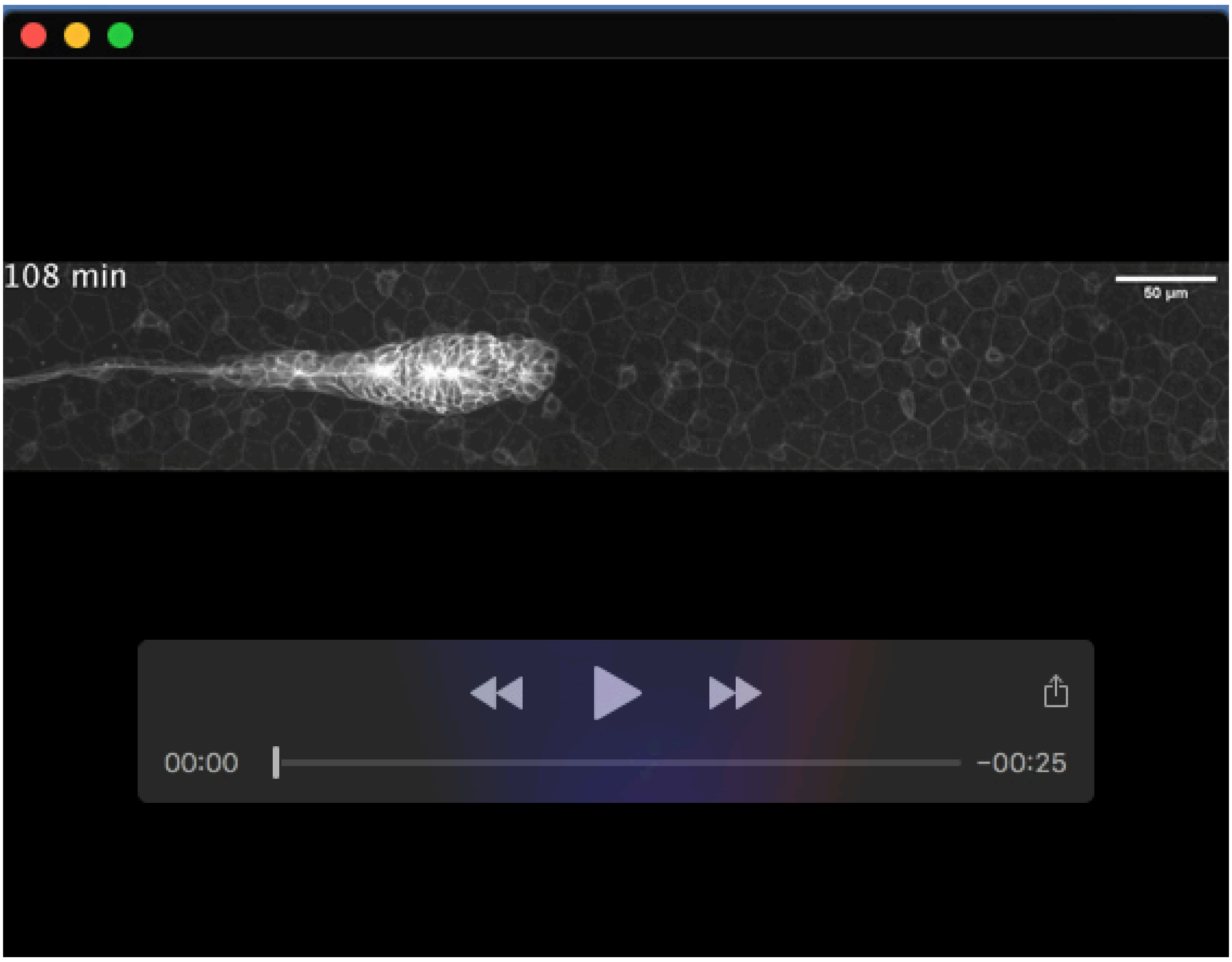

Movie 10. Rosette fusion in a *lama5;itga6b* double mutant pLLP, related to Figure 6.

Development • Supplementary information

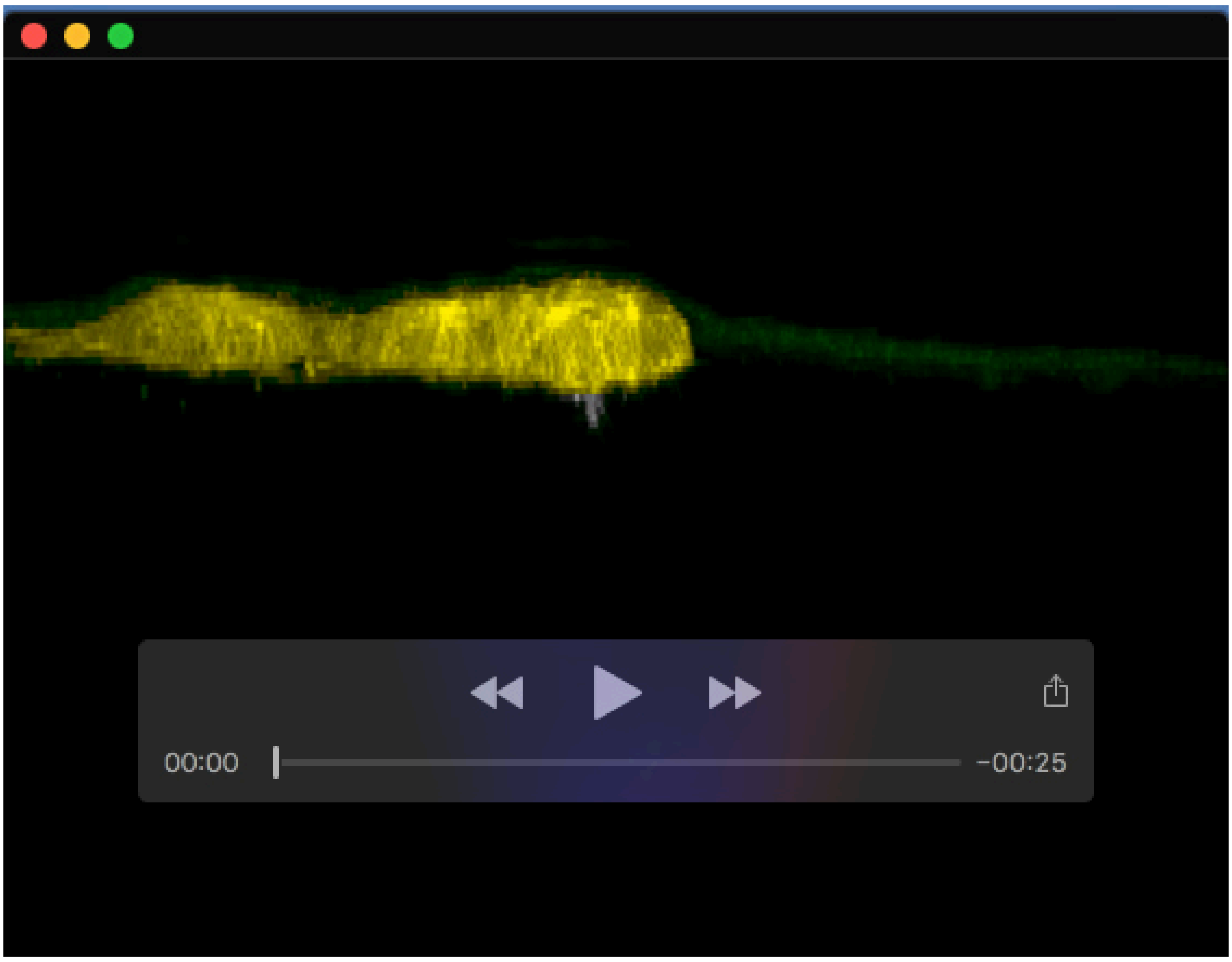

Movie 11. Invadopodia-like basal protrusions in a *lama5;itga6b* double mutant pLLP.

Timelapse of a *lama5;itga6b* double mutant pLLP, cell membranes labelled with *cldnb:GFP,* shown is 1h of migration captured at 2min intervals. 3D animation created in Imaris, pseudocolored invadopodia (grey), pLLP (yellow) and skin cells (green).

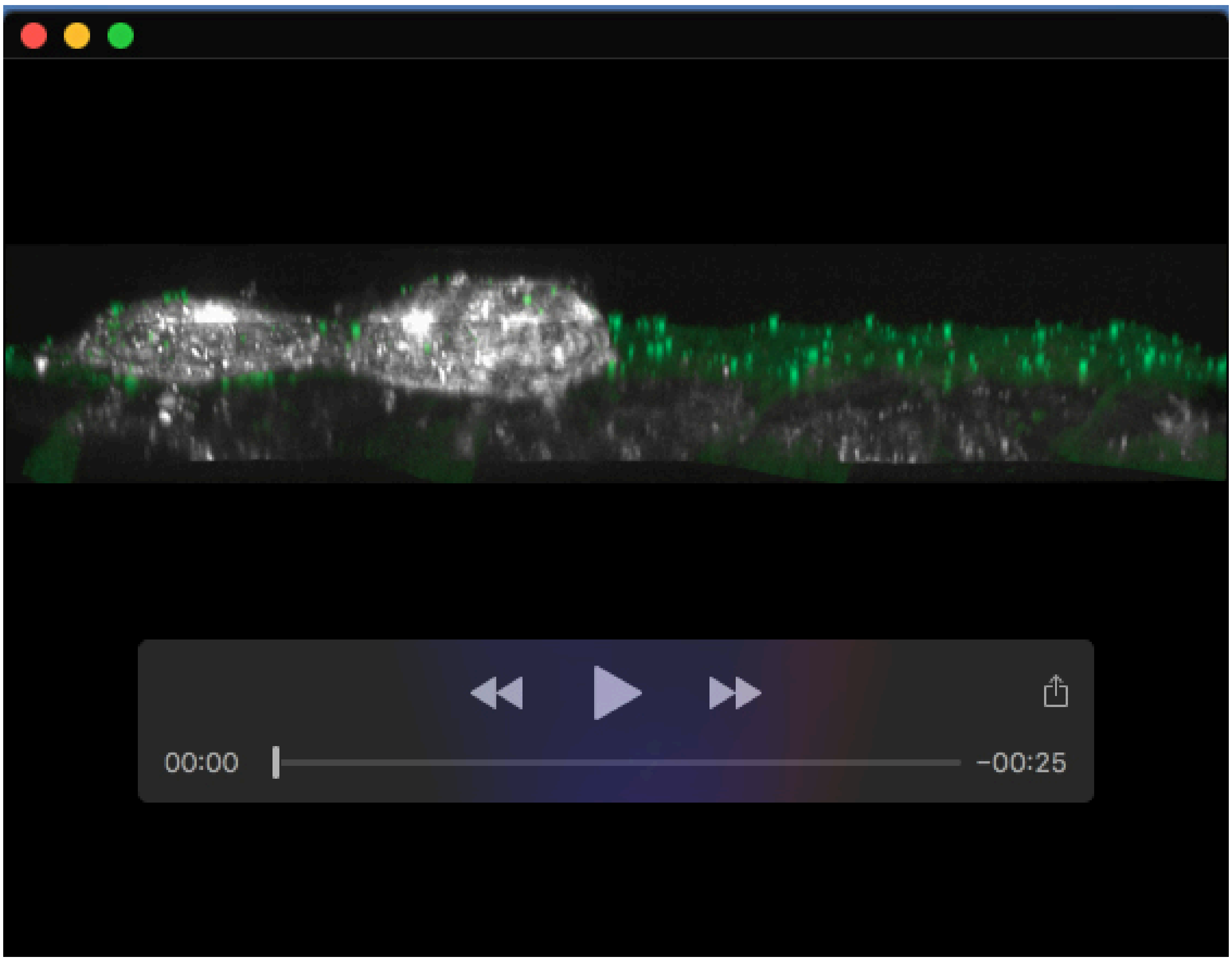

Movie 12. Invadopodia-like protrusions invade subepidermal tissue through BM holes.

Timelapse of a *lama5;itga6b* double mutant pLLP labelled with *cxcr4b:R-Lifeact* (pLLP, muscle) and the BM labelled with *lamc1:lamc1-GFP*, shown is 1h of migration captured at 2min intervals. Green punctae depict Lamc1-GFP accumulati-ons in basal skin cells.

Table S1. List of primers used for genotyping, qPCR and cloning, including their sequences and annealing temperature

Name	Sequence	Annealing (°C)
geno lama5 fwd	GGCAACACGCCCTGTAAATA	57
geno lama5 rev	CCTAATGAGTGAGCGTGCAG	
geno itga3a fwd	CCGGAAGAATGACGAATATGA	53
geno itga3a rev	CTCAACAGGTGCACGTCAGT	
geno itga3b fwd	GGTCCCAGTGGTTTTCAGACGTTTGGATAT	56
geno itga3b rev	CACAAGAGGGATTATGTGATGATG	
geno itga6b fwd	CGGGGCTGTATTAGCAGGTA	57
geno itga6b rev	GTAGTGAGGCGCGACGTTAT	
qPCR itga3a fwd	GACATCCCAGGTGGAGGATT	60
qPCR itga3a rev	CACAGGACGTGATCTGAGCAA	
qPCR itga3b fwd	CGGTCACTCCTAGCCACATT	60
qPCR itga3b rev	AACCAGGATGACTGCGGAAG	
qPCR itga6b fwd	CATCAACCGGCTTAGACAGC	60
qPCR itga6b rev	TTCCCTCTGCTGCTGAAGTTT	
qPCR rpl13 fwd	TAAGGACGGAGTGAACAACCA	60
qPCR rpl13 rev	CTTACGTCTGCGGATCTTTCTG	
ISH itga3a fwd	TGCAGGAGATTGAGACAGAGC	55
ISH itga3a rev	TCCAATAATTTCTTGTCCCACA	
ISH itga3b fwd	GCTGGTTTTGATATGGAGTGC	55
ISH itga3b rev	GAGACAGAGAGGCTGACGGAG	
ISH itga6a fwd	ACCGCTTCGCTAAACATCC	55
ISH itga6a rev	TAACATGACCTTCCCGTCC	
ISH itga6b fwd	CAGGCAAAGACGAAATGGAT	55
ISH itga6b rev	TTGTAATCACCGCTGTCCAA	
ISH itga6l fwd	TGGAGGAGAAGTGAAAGATGAG	55
ISH itga6l rev	CAACAGGTAGAAGAGCAAAGC	
ISH itgb1a fwd	CTCGACACCTCCACCTGTCT	55
ISH itgb1a rev	CTGCTACTGTCCACCAGACG	
ISH itgb1b fwd	TGAGGAGTTCCAGCCTGTTT	55
ISH itgb1b rev	TGCCCTCATATTTAGGGTTGA	
