# Supplementary material for: Laminin α5 and integrins α3 and α6 coordinately regulate collective cell migration in vivo
Source: Development. 2026 Jun 11;153(16):dev205312. doi: 10.1242/dev.205312 (PMC13286361; doi:10.1242/dev.205312)
Supplement: Supplementary file 1 [file develop-153-205312_review_history.pdf]

Laminin α5 and integrins α3 and α6 coordinately regulate collective cell migration *in vivo*

Anna Mertens, Nicola Moratscheck, Petra A. Klemmt, Chaitanya Dingare, Melanie Heyde, Marion Basoglu, Stefan Eimer and Virginie Lecaudey

DOI: 10.1242/dev.205312

Editor: Rashmi Priya

Review timeline

Original submission:	17 October 2025
Editorial decision:	9 December 2025
First revision received:	27 March 2026
Accepted:	29 April 2026

Original submission

First decision letter

MS ID#: dev.205312

MS TITLE: Patterned integrin-laminin adhesion coordinates epithelial collective cell migration in vivo.

AUTHORS: Anna Mertens, Nicola Moratscheck, Petra A. Klemmt, Chaitanya Dingare, Marion Basoglu and Virginie Lecaudey

Dear Dr Lecaudey,

I have now received all the referees' reports on the above manuscript, and have reached a decision. The referees' comments are appended below.

As you will see, the referees express considerable interest in your work, but have some significant criticisms and recommend a substantial revision of your manuscript before we can consider publication. If you are able to revise the manuscript along the lines suggested, which may involve further experiments, I will be happy to receive a revised version of the manuscript. Your revised paper will be re-reviewed by one or more of the original referees, and acceptance of your manuscript will depend on your addressing satisfactorily the reviewers' major concerns. Please also note that Development will normally permit only one round of major revision. If it would be helpful, you are welcome to contact us to discuss your revision in greater detail. Please send us a point-by-point response indicating your plans for addressing the referees’ comments, and we will look over this and provide further guidance.

Please attend to all of the reviewers' comments and ensure that you upload both a 'clean' version of your Word file, along with a highlighted version clearly showing where you have made changes in the revised manuscript. Please avoid using 'Tracked changes' in Word files as these are lost in PDF conversion. I should be grateful if you would also provide a point-by-point response detailing how you have dealt with the points raised by the reviewers in the 'Response to Reviewers' box. If you do not agree with any of their criticisms or suggestions please explain clearly why this is so.

Reviewer 1

© 2026. Published by The Company of Biologists under the terms of the Creative Commons Attribution License (https://creativecommons.org/licenses/by/4.0/).

Advance summary and potential significance to field

In their manuscript, Moratschek et al assess how integrin-mediated adhesion contributes to primordium migration. They start out by surveying which integrins are expressed in the primordium and identify itga3a, itga3b, itga6b and itgb1b. Next, the authors test whether any of these integrin subunits is required for primordium migration using existing and newly generated mutants. They found that itga3a and itga3b did not show a primordium migration defect and itga6b showed slightly slowed migration. Using double and triple mutant combinations, the authors find that itga3b; itga6b double mutants had slowed migration which was enhanced in itga3a; itga3b; itga6b triple mutants. Slowed migration was also reflected in increased protrusive activity in directions other than the direction of migration.
The requirement of integrins for efficient primordium migration prompted the authors to investigate the role of the putative integrin ligand laminin in migration. For this they used a mutant in the laminin a5 subunit. Intriguingly, lama5 mutant embryos still form a basement membrane but the basement membrane is punctured. Despite a discontinuous basement membrane in lama5 mutants, primordium migration was not affected. However, when the authors also reduced the activity of itga6b the primordium migrated slower than in single mutants, indicating that lama5 and itga6b are likely required for partly non-overlapping primordium-basement membrane adhesion and thus efficient migration. This is a beautiful genetic study of the role of integrins in primordium migration. A previous study showed that integrin-b1b and an intact basement membrane are required for (efficient) primordium migration but did not address which integrin dimers and which laminins are required for this process. Thus, the study adds important detail to this process and is well-suited for development. The figures are well-laid out, the movies are beautiful, and the documentation is comprehensive. This is one of these rare cases where the manuscript is ready to be published in its current form.

Reviewer 2

Advance summary and potential significance to field

In this study, Moratscheck et al., examine how integrin-laminin interactions regulate collective cell migration of the posterior lateral line primordium (pLLP) in the zebrafish. Authors have employed classical in situ hybridisation (ISH) to identify differential expression of itga6b in the leading region and itga3a/itga3b in the trailing region. They further use combinations of mutants of these 3 integrins to propose a hierarchical and partially redundant adhesion machinery. They show that Lama5 is a basement membrane (BM) component underneath the pLLP, and that while lama5-/- mutants display BM discontinuities, pLLP migration remains largely normal unless combined with the loss of itga6b due to genetic interaction, leading to more pronounced migration defects and eventual stalling.
This manuscript provides a dataset combining genetics, ISH, live imaging and EM and makes a potentially significant contribution to the understanding of collective cell migration in vivo. However, several key aspects of the logic, quantification, interpretation, and completeness of the data require clarification or additional explanation. Many conclusions are plausible but not sufficiently supported by the current data presentation. Outlined below are major points where the manuscript would benefit from additional experiments, deeper explanation, expanded quantification, and clearer figure support.

Comments for the author

Major points:
1. Morphology and migration claims need more quantitative support (Fig. 2A-C"). The authors conclude that 'Both pLLP morphology and migration appeared unaffected in itga3b-/- embryos' (lines 133-134). While migration is quantified, morphology is not. Because pLLP shape and aspect ratio later become central metrics in the manuscript (e.g. elongated leading region in integrin mutants; round pLLP in lama5 mutants), it will be important to quantify them in these early genotypes. Without quantitative analysis of primordium length, width, aspect ratio, or leading/trailing region proportions, claims of no morphological defects cannot be fully evaluated

© 2026. Published by The Company of Biologists under the terms of the Creative Commons Attribution License (https://creativecommons.org/licenses/by/4.0/).

and may obscure subtle yet biologically meaningful differences. Please quantify pLLP shape for the integrin mutants, ideally using the same metrics employed later in Fig. 4 as a starting point.

2. Migration data is missing from itga3a-/-;itga3b-/-;itga6a+/+ conditions (Fig.S3). It refers to the absence of phenotypes in double itga3a/itga3b mutants but the migration tracks of distance quantifications for this exact combination (with itga6a WT) are not shown. This combination is essential for supporting the claim that a3 paralogues are dispensable for migration in the absence of a6b, and for establishing the integrin 'hierarchy'. Please include the corresponding images and migration quantifications or indicate more clearly where they can be found.

3. Authors show an expansion of the leading region in itga3b, itga6b double and triple mutants (Fig. 4A) but do not quantify whether total primordium length or trailing region length is also altered. An elongated leading region could reflect different things like overextension of leading cells, global stretching due to reduced adhesion or increased tension etc. Without total length and leading region to trailing region length quantifications, it is not possible to draw conclusions from the many possibilities.

4. There is ambiguity in protrusion origin in Fig. 4E-F. Authors state that mutants display extensive filopodia and lamellipodia but the figures shown are maximum-intensity projections, making it impossible to assess whether protrusions arise from the basal, apical, or both surfaces. This distinction is critical because prior work (Olson and Nechiporuk, 2021, Developmetal Biology) demonstrated focal adhesions in the pLLP and that the apical surface is also key for migration (Nogare et al., 2020, eLife). Please provide single-plane basal views, orthogonal cross-sections, or additional analysis to clarify z-position of protrusions.

5. Please quantify lamellipodia length, persistence and/or direction in Fig. 4G. Lamellipodia length and persistence are linked to adhesion stabilisation and traction force generation. A mutant might show more protrusions but not directionally polarised which could help explain the migration phenotype so consider quantifying mean lamellipodia length or protrusion area.

6. Authors did not address the possibility of compensation between integrin paralogues. Transcriptional adaption is common in zebrafish especially when multiple paralogues are present. The hierarchy model depends strongly on ruling out compensatory upregulation. Please examine the expression of igta3a in itga3b/itga6b mutants, itga3b in itga3a/itga6b mutants and itga6b in itga3a/itga3b muntants. HCR would be ideal but even qPCR from sorted pLLP cells would strengthen this claim.

7. Laminin signal at cell membrane may be non-specific in Fig. 5B-B'. Authors state that laminins are detected at pLLP cell membranes and rosette centres. Can authors please clarify whether these signals are biological or background by providing individual z-slices and proper controls, validation of this antibody by showing known laminin localisation patterns in cell and tissue types (other than the pLL system). Authors will also need to suggest reasons for laminin at cell-cell interfaces and rosette centres.

8. Basement membrane gap quantification in Fig. 5J of gaps in the skin lack representative images. These gaps become key to the BM interpretation and the interaction with lamC1 so need visual support. The quantification is helpful but without images illustrating the types of gaps scores, the reader cannot judge morphology, size or consistency. Please include representative images of the quantified gaps in supplementals.

9. The adepidermal granules in Fig. S4A-A' require more explanation. Authors mention loss of adepidermal granules in lama5 mutants but do not explain what they are, their developmental function or why their absence matters for FM integrity. Without context, readers unfamiliar with epidermal ultrastructure cannot interpret the significance of their disruption. Please add brief explanation and also annotate the TEM panels more clearly so the reader can orient themselves to understand the image (epidermis, BM pLLP, migration direction etc.)

10. The INC gaps in the lama5-/- embryos are striking however, authors do not show whether the BM discontinuities correlate with INC chain instability. If INC detachment coincides with BM

© 2026. Published by The Company of Biologists under the terms of the Creative Commons Attribution License (https://creativecommons.org/licenses/by/4.0/).

gaps, this would strongly support Lama5's role in providing a continuous substrate for both the pLLP and INCs. Please provide LamC1-GFP and/or pan-laminin images specifically beneath INCs.

11. The text states there is no obvious differences seen in the actin organisation in the leading region of lama5 mutants in Fig. 6H-I". However visually there appears to be increased actin intensity at the base of the protrusions at the cell cortex in mutants. Please quantify actin intensity distribution and/or protrusion base width in the leading region cells.

12. The conclusion that lama5 mutants must use alternative receptors because their do not phenocopy integrin mutants is speculative (lines 298-300). Rephrase to avoid conclusions not directly supported by data.

13. Authors state that mild delay occurs in lama5 single mutants (lines 306-307) but itga6b mutants also show a comparable mild delay. Please revise text to reflect that they both share this mild phenotype.

14. Rosette fusion phenotype should acknowledge related work (lines 334-336). Here authors describe leading region splitting and rosette fusion in lama5, itga6b double mutants. Mukherjee et al., 2025, bioRxiv, reported tension-dependent rosette fusion in the pLLP so please cite this as it supports the mechanical basis of this phenotype.

15. In the discussion the authors' interpretation of front versus rear force contributions requires refinement. The authors' conclusion that a shortened leading region in lama5 mutants supports 'movement driven primarily by pushing from the rear' (lines 435-437) is not fully convincing. Authors show that Itga6b is expressed in the leading region but has the most severe single-mutant phenotype and also loss of itga6b reduces primordium speed more than loss of itga3a or itga3b. Please discuss a model where both rear pushing and front traction contribute and where leading region-specific adhesions provide directional stabilisation rather than just propulsion.

Reviewer 3

Advance summary and potential significance to field

This work by Moratscheck et al. describes the role of Integrin receptors alpha6b and alpha3 and their extracellular matrix substrate Laminin alpha5 on collective cell migration in vivo, using zebrafish posterior lateral line primordium (pLLP) as a model. The authors provide gene expression and protein localization data for the basement membrane using pan-Laminin and Laminin alpha5 and a reporter line expressing GFP-tagged Laminin gamma1, and uncovered strong deposition in the layer beneath the migrating pLLP cells. They also examined the expression of three of the best-known Integrin alpha subunits binding to basement membrane factors, and uncovered an interesting expression pattern: Integrin alpha3a and b mostly restricted in the rear/lagging end of the migrating pLLP, and Integrin alpha6b in the front/leading end of the migrating pLLP. The authors generated mutations in three different integrin genes, itga3a, itga3b and itg6a, and analyzed an additional mutation in lama5. Among the integrins, itga6b mutations were the only one that slightly delayed migration, but double and triple itga3/itga6b mutations impaired migration more strongly and produced more lateral protrusions. Focal adhesion activation, as assessed by phosphorylated FAK, appears unaffected in the double/triple integrin mutants. lama5 mutations strongly delayed migration, and caused cluster rounding in the pLLP and interneuromast chains. These effects became more pronounced in lama5, itga6b double mutants, with significantly reduced migration speed and cell rounding. This synergistic exacerbation suggests that Laminin alpha5 and Integrin alpha6 promote pLLP migration through mechanisms that are mostly independently of each other.

Overall, this study presents an interesting case study of the role of the extracellular matrix in promoting collective cell migration in vivo. The study is particularly strong in their genetic data; generating and analyzing triple integrin mutants was an impressive undertaking. The analysis method of migration success and cluster morphology is solid. However, the study could benefit from a stronger focus and analysis of the phenotypes from a cell biological perspective.

Below are my major comments to strengthen the conceptual advance of the manuscript:

© 2026. Published by The Company of Biologists under the terms of the Creative Commons Attribution License (https://creativecommons.org/licenses/by/4.0/).

- The mechanistic insight of the loss-of-function phenotypes is still unclear. Considering the distinct expression patterns of the integrins - itga3 in the rear end, itga6b in the front end of the migrating cluster - are the effects of the mutations on cell migration due to genetic redundancy or separate effects on the rear/trailing and front/leading cells of the cluster? Aside from the migrating success and distance, the authors did not report other changes in cellular behaviours in the cluster. It would be interesting to examine in detail the regional effects (e.g. front-rear polarization, protrusion activities in different cells of the cluster, protrusion retraction activities, cell numbers in different regions of the pLLP) of loss of itga3a, itga3b and itga6b alone or in combination. Overall, the study could really benefit from better analysis of the regional differences in cell behaviours and of the rear vs. front polarization within the pLLP.
- The phospho-FAK immunostaining appears stronger in the triple mutants, with potentially altered localization. Can the authors comment on whether there are quantifiable changes in its localization? What are the possible downstream effectors if focal adhesion-related factors are not affected? Have the authors tested the localization of other FA factors, such as Vinculin, Talin or Paxillin?
- Interneuromast cells change their behaviours to become more protrusive in lama5 mutants. Are there any changes in the neuromast size, organization or individual cell behaviours?
- Considering the changes in morphology of the migrating clusters in lama5 mutants, could there be a change in the mode of migration, e.g. from lamellipodia-based protrusions to a more amoeboid migration mode, when the ECM is affected? Can the authors test this by closely examining localization or expression of basolateral polarity or actomyosin markers of the migrating cells?
- What is the significance of the tissue holes seen in lama5 mutants? If the authors can speculate, why is the irregular substrate presence not impacting pLLP migration?
- The authors concluded that the effects of Laminin alpha5 and Integrin alpha6 and alpha3 are mostly independent of each other; in the Discussion, they wrote that the primary ligands of the Integrins are not Laminins, and the primary receptor for Laminin alpha5 is not Integrin alpha3 and 6. Thus, there are missing pathways by which the ECM ligands and receptors interact with each other. Can the authors provide some data as to what these missing links could be: from in situs, immunostaining or citing known literature on expression patterns of other key focal adhesion-related molecules? For instance, the authors speculated about Integrin alpha7 in the Discussion - what is the expression pattern of this alpha subunit? Is there also a possibility for an Integrin-independent mechanism in this migrating cell type by which Laminins exert their effects?
- Are there specific experiments that the authors can do to more strongly support their hypothesis of the rear-driven migration? For instance, AFM, visualization of actin flow or Integrin beta1 and Talin1 localization in relation to the migrating cluster, as performed in Yamaguchi et al. 2022. In particular, it is very interesting that the mutations in integrin alpha3a and 3b, which were enriched in the lagging end of the pLLP, had the mildest effects on migration; can the authors comment on why this could be (genetic redundancy with other Integrin alpha subunits, lack of need for Integrins in the rear end propelling of the cell clusters)?
- Minor comment: The title "Patterned Integrin-Laminin adhesion…" is a little vague, clearer rewording might be needed to better describe the work.

First revision

Author response to reviewers' comments

Reviewer 1:

In their manuscript, Moratschek et al assess how integrin-mediated adhesion contributes to primordium migration. They start out by surveying which integrins are expressed in the primordium and identify itga3a, itga3b, itga6b and itgb1b. Next, the authors test whether any of these integrin subunits is required for primordium migration using existing and newly generated mutants. They found that itga3a and itga3b did not show a primordium migration defect and itga6b showed slightly slowed migration. Using double and triple mutant combinations, the authors find that itga3b; itga6b double mutants had slowed

© 2026. Published by The Company of Biologists under the terms of the Creative Commons Attribution License (https://creativecommons.org/licenses/by/4.0/).

migration which was enhanced in itga3a; itga3b; itga6b triple mutants. Slowed migration was also reflected in increased protrusive activity in directions other than the direction of migration.

The requirement of integrins for efficient primordium migration prompted the authors to investigate the role of the putative integrin ligand laminin in migration. For this they used a mutant in the laminin a5 subunit. Intriguingly, lama5 mutant embryos still form a basement membrane but the basement membrane is punctured. Despite a discontinuous basement membrane in lama5 mutants, primordium migration was not affected. However, when the authors also reduced the activity of itga6b the primordium migrated slower than in single mutants, indicating that lama5 and itga6b are likely required for partly non-overlapping primordium-basement membrane adhesion and thus efficient migration. This is a beautiful genetic study of the role of integrins in primordium migration. A previous study showed that integrin-b1b and an intact basement membrane are required for (efficient) primordium migration but did not address which integrin dimers and which laminins are required for this process. Thus, the study adds important detail to this process and is well-suited for development. The figures are well-laid out, the movies are beautiful, and the documentation is comprehensive. This is one of these rare cases where the manuscript is ready to be published in its current form.

We sincerely thank the reviewer for their careful evaluation of our manuscript and very positive comments on the work.

Reviewer 2:

SUMMARY OF THE ADVANCE MADE IN THIS PAPER AND ITS POTENTIAL SIGNIFICANCE TO THE FIELD

In this study, Moratscheck et al., examine how integrin-laminin interactions regulate collective cell migration of the posterior lateral line primordium (pLLP) in the zebrafish. Authors have employed classical in situ hybridisation (ISH) to identify differential expression of itga6b in the leading region and itga3a/itga3b in the trailing region. They further use combinations of mutants of these 3 integrins to propose a hierarchical and partially redundant adhesion machinery. They show that Lama5 is a basement membrane (BM) component underneath the pLLP, and that while lama5-/- mutants display BM discontinuities, pLLP migration remains largely normal unless combined with the loss of itga6b due to genetic interaction, leading to more pronounced migration defects and eventual stalling.

This manuscript provides a dataset combining genetics, ISH, live imaging and EM and makes a potentially significant contribution to the understanding of collective cell migration in vivo. However, several key aspects of the logic, quantification, interpretation, and completeness of the data require clarification or additional explanation. Many conclusions are plausible but not sufficiently supported by the current data presentation. Outlined below are major points where the manuscript would benefit from additional experiments, deeper explanation, expanded quantification, and clearer figure support.

We thank the reviewer for the careful evaluation of our manuscript and the detailed suggestions. We have revised the manuscript accordingly, as outlined in the point-by- point responses below.

SUGGESTIONS TO AUTHORS

Major points:

1. Morphology and migration claims need more quantitative support (Fig. 2A-C"). The authors conclude that 'Both pLLP morphology and migration appeared unaffected in itga3b-/- embryos' (lines 133-134). While migration is quantified, morphology is not.

Because pLLP shape and aspect ratio later become central metrics in the manuscript (e.g. elongated leading region in integrin mutants; round pLLP in lama5 mutants), it will be

© 2026. Published by The Company of Biologists under the terms of the Creative Commons Attribution License (https://creativecommons.org/licenses/by/4.0/).

important to quantify them in these early genotypes. Without quantitative analysis of primordium length, width, aspect ratio, or leading/trailing region proportions, claims of no morphological defects cannot be fully evaluated and may obscure subtle yet biologically meaningful differences. Please quantify pLLP shape for the integrin mutants, ideally using the same metrics employed later in Fig. 4 as a starting point.

We thank the reviewer for pointing this out and agree that the claim about unaffected morphology benefits from additional quantitative support. As requested, we additionally quantified pLLP roundness in all single integrin mutants and found no difference, this new data is presented in **Fig. S2J-L**. Related to this point and to answer major point (3) of reviewer 2, we also added the total length of the pLLP and of the leading region (LR) to the supplementary material in **Fig. S3M-N**. The LR/total pLL length ratio was in fact already provided for single mutants as part of the characterization of the double/triple mutant phenotype in **Fig. 4D.** These data together demonstrate no difference in pLLP length, leading/trailing proportions nor roundness in single integrin mutants.

2. Migration data is missing from itga3a-/-;itga3b-/-;itga6a+/+ conditions (Fig.S3). It refers to the absence of phenotypes in double itga3a/itga3b mutants but the migration tracks of distance quantifications for this exact combination (with itga6a WT) are not shown. This combination is essential for supporting the claim that a3 paralogues are dispensable for migration in the absence of a6b, and for establishing the integrin 'hierarchy'. Please include the corresponding images and migration quantifications or indicate more clearly where they can be found.

We thank the reviewer for pointing this out. Given the 27 possible genetic combinations obtained from triple heterozygous incrosses and to avoid overwhelming the reader, we felt it best to pool wild-type and heterozygote embryos when they did not show phenotypic differences. This was verified before in experiments done with double heterozygous incrosses but we acknowledge this was not clearly communicated in the manuscript.

To address this point we:

(i) Added a representative image of an *itga3a-/-;itga3b-/-;itga6b+/+* embryo in **Fig. S3F-F'**

(ii) Added data to quantitatively support the decision of pooling double homozygous; single heterozygous and double homozygous; single wild-type embryos: to increase the statistical power, we expanded the dataset by combining migration data for *itga3a;itga3b* (N=3), *itga3a;itga6b* (N=3) as well as *itga3b;itga6b* (N=3) double heterozygous incrosses with the triple heterozygous incrosses (N=3) shown in the initial manuscript. Using this data, we now provide an additional graph demonstrating no significant difference between double homozygous; single heterozygous and double homozygous; single wild- type combinations, including the specifically addressed *itga3a-/-;itga3b-/-;itga6b+/+* vs. *itga3a-/-;itga3b-/-;itga6b+/-* mutants **(Fig S3L).** We revised the manuscript to better reflect our justification for pooling **(lines 165-168)**

3. Authors show an expansion of the leading region in itga3b, itga6b double and triple mutants (Fig. 4A) but do not quantify whether total primordium length or trailing region length is also altered. An elongated leading region could reflect different things like overextension of leading cells, global stretching due to reduced adhesion or increased tension etc. Without total length and leading region to trailing region length quantifications, it is not possible to draw conclusions from the many possibilities.

We understand the issue of interpretation of an elongated leading region and thank the reviewer for pointing this out. To address this concern, we have now included graphs displaying the measurements of leading region and total pLLP length separately in **Fig. S3M-N.** These data show that the leading region is significantly longer in the integrin *3b;6b* double and triple mutants, whereas total pLLP length is only slightly increased in the triple mutants. Thus, the change in leading/total length ratio presented in **Fig. 4A-D** reflects a specific elongation of the leading region rather than a proportional increase in overall primordium length. We have revised the corresponding section to specifically state

© 2026. Published by The Company of Biologists under the terms of the Creative Commons Attribution License (https://creativecommons.org/licenses/by/4.0/).

"extended proneuromast-free leading region (LR), both in absolute value and relative to the total length of the primordium (**Fig. 4A-D, S3M**)" (**lines 185-186**).

4. There is ambiguity in protrusion origin in Fig. 4E-F. Authors state that mutants display extensive filopodia and lamellipodia but the figures shown are maximum-intensity projections, making it impossible to assess whether protrusions arise from the basal, apical, or both surfaces. This distinction is critical because prior work (Olson and Nechiporuk, 2021, Developmetal Biology) demonstrated focal adhesions in the pLLP and that the apical surface is also key for migration (Nogare et al., 2020, eLife). Please provide single-plane basal views, orthogonal cross-sections, or additional analysis to clarify z- position of protrusions.

We thank the reviewer for raising this important point. This comment highlighted that the terms "filopodia" and "lamellipodia" were used inconsistently in the original manuscript. The phenotype we describe and quantify primarily concerns the **filopodial protrusions** of the pLLP. While filopodia can emerge from lamellipodia-like structures at the leading or lateral edges, lamellipodia themselves were not the focus of our analysis. We revised the manuscript and now only use the general term "protrusions" when talking about an overall protrusive appearance and "filopodia" specifically concerning our analyses (**lines 186-202** integrin mutant filopodia quantification; **line 288** *lama5* INCs; **lines 315-319** *lama5* mutant).

To clarify the spatial origin of these protrusions, we now provide representative digital sections in WT and triple integrin mutants in **Fig. S4H**. These images show that the filopodia do not arise strictly from the apical or basal surfaces, but rather from the lateral interface of the pLLP between the skin and basement membrane. Interestingly, we also noticed that many filopodia are directed apically, with their tips frequently contacting the overlying basal epidermal cells **(Fig. S4I).**

5. Please quantify lamellipodia length, persistence and/or direction in **Fig. 4G**. Lamellipodia length and persistence are linked to adhesion stabilisation and traction force generation. A mutant might show more protrusions but not directionally polarised which could help explain the migration phenotype so consider quantifying mean lamellipodia length or protrusion area.

As noted above, our analysis focuses on **filopodial protrusions** rather than lamellipodia. Reliable quantification of lamellipodia was not straightforward in this context, as pLLP cells frequently lack a classical lamellipodial morphology. Furthermore, lamellipodia are seldom confined to a single z-plane and are often partially obscured by neighbouring cells, making consistent quantification challenging.

Nonetheless, we agree that quantitative analysis of the protrusion properties is important. We therefore expanded our analysis to a comprehensive quantification of filopodia in pLLPs of single, double, and triple integrin mutants. Specifically, we measured **filopodia abundance, orientation, and length** around the entire pLLP perimeter (**Fig. 4 and Fig. S4, lines 188-202**).

Consistent with our initial analysis, triple mutants displayed a significant increase in filopodia number. Notably, this precise quantification revealed also an increase in filopodia abundance in *itga3b* single mutants and in *itga3b;6b* and *itga3a;3b* double mutants (**Fig. 4G**). In contrast, the **average filopodia length was reduced** in all these mutants (**Fig. 4H**). Directionality was also mildly impaired, as indicated by an increased angular deviation from the direction of migration (**Fig. 4F, S4D-E**). Finally, this analysis allowed us to show that leading filopodia (see **Fig. S4A** for distinction, light green) were generally longer than lateral and rear filopodia (dark green) in controls, and that this gradient was almost absent in *itga3a;3b;6b* triple mutants (**slope in S4C**), further supporting loss of directionality in these mutants.

6. Authors did not address the possibility of compensation between integrin paralogues. Transcriptional adaption is common in zebrafish especially when multiple paralogues are present. The hierarchy model depends strongly on ruling out compensatory upregulation.

© 2026. Published by The Company of Biologists under the terms of the Creative Commons Attribution License (https://creativecommons.org/licenses/by/4.0/).

Please examine the expression of igta3a in itga3b/itga6b mutants, itga3b in itga3a/itga6b mutants and itga6b in itga3a/itga3b mutants. HCR would be ideal but even qPCR from sorted pLLP cells would strengthen this claim.

We thank the reviewer for raising this point and agree that potential genetic compensation is an important consideration. In the original manuscript we provided qPCR data from MZ *itga3b* embryos, which did not reveal increased transcript levels of *itga3a* or of *itga6b*, arguing against compensatory upregulation/transcription adaptation in this context (unchanged, **lines 135-142** in revised manuscript).

We agree that a systematic analysis across all mutant combinations would be informative. However, this is technically challenging, as *itga3a* and *itga6b* mutants do not survive to adulthood, and—except for *itga3b*—single and double mutant embryos cannot be reliably distinguished phenotypically from their siblings for bulk or cell-sorting-based approaches. As an alternative, we performed in situ hybridization (ISH), but obtained robust results only for *itga3b* expression, which did not show altered expression in *itga3a;itga6b* embryos. This new data is presented in **Fig. S2M-P, lines 182-183**. Combined with our qPCR data on MZ *itga3b* mutants, this shows that neither *itga3a* or *itga6b* are transcriptionally upregulated in absence of *itga3b* (**Fig. S2I**), nor *itga3b* in absence of *itga3a*, *itga6b*, or both (**Fig. S2M-P**). Importantly, we also provide new data showing that Itgb1b localization reflects *itga3*/*itga6* expression patterns in the trailing/leading region, respectively (**Fig. 4K-P**), further ruling out the possibility of full compensation through upregulation of the paralogues.

While we acknowledge that mutation-induced transcriptional adaptation cannot be formally excluded in our system, we would like to emphasize the key implications of our genetic analysis: since *itga3a*, *itga3b* and *itga6b* are co-expressed in the migrating pLLP in wild-type, the progressive phenotypic enhancement from single *itga6b* to *itga3b;6b* double to *itga3a;3b;6b* triple mutants is fundamental evidence that these integrins **functionally compensate**, to different degrees, for one another. This conclusion is independent of the underlying molecular mechanism - be it adaptions on transcript or protein levels. Finally, our expression analysis argues against transcriptional adaptation in our integrin mutants, although we can not exclude transcriptional adaptation of other genes.

7. Laminin signal at cell membrane may be non-specific in Fig. 5B-B'. Authors state that laminins are detected at pLLP cell membranes and rosette centres. Can authors please clarify whether these signals are biological or background by providing individual z-slices and proper controls, validation of this antibody by showing known laminin localisation patterns in cell and tissue types (other than the pLL system). Authors will also need to suggest reasons for laminin at cell-cell interfaces and rosette centres.

We thank the reviewer for pointing this out. Upon careful re-analysis of the image data, we agree that the pan-laminin signal observed at pLLP cell membranes is unlikely to represent extracellular laminin localization and most likely reflects non-specific staining. We have therefore removed the statement from the manuscript and revised the text to focus exclusively on areas of high pan-laminin signal associated with the basement membrane **(lines 240-243)**.

To address the reviewer's concerns regarding specificity and localization, we now provide individual z-slices highlighting established laminin-rich structures, including the epidermal basement membrane and the somite boundaries (**Fig. S7A**).

8. Basement membrane gap quantification in Fig. 5J of gaps in the skin lack representative images. These gaps become key to the BM interpretation and the interaction with lamC1 so need visual support. The quantification is helpful but without images illustrating the types of gaps scores, the reader cannot judge morphology, size or consistency. Please include representative images of the quantified gaps in supplementals.

We have included representative images of BM holes in regions outside the pLLP migration path in **Fig. S7E** including 2 orthogonal views (a and b) to accompany the measurements presented in **Fig. 5J** and facilitates evaluation of the consistency of the phenotype.

© 2026. Published by The Company of Biologists under the terms of the Creative Commons Attribution License (https://creativecommons.org/licenses/by/4.0/).

We have also decided to consistently rename these “holes” instead of “gaps” to clearly differentiate from the INCs gaps, which we also describe in these mutants. In addition to these BM holes, we now also describe large “voids” separating the basal cells and the basement membrane. These voids are found exclusively in the pLLP wake, suggesting that they are a consequence of the pLL migration and are further described below in our answer to comment (10).

9. The adepidermal granules in Fig. S4A-A' require more explanation. Authors mention loss of adepidermal granules in lama5 mutants but do not explain what they are, their developmental function or why their absence matters for FM integrity. Without context, readers unfamiliar with epidermal ultrastructure cannot interpret the significance of their disruption. Please add brief explanation and also annotate the TEM panels more clearly so the reader can orient themselves to understand the image (epidermis, BM pLLP, migration direction etc.)

Adepidermal granules (aeg) are transient extracellular structures associated with basement membrane formation during early development in aquatic vertebrates, including zebrafish (Weiss and Ferris, 1954; Nadol et al., 1969; Watanabe and Tachibana, 1973). We have revised the manuscript to include a brief explanation of aeg with additional citations **(lines 272-274)** while avoiding an extended introduction and discussion, as their functional role is not well established and lies outside the main scope of this study. In the context of our data, we limit our interpretation to the descriptive observation that these structures are reduced in *lama5* mutants.

As requested, we have also added clearer annotations to the TEM panel in **Fig. S7B**, including the epidermis, basement membrane and pLLP as well as migration direction, to facilitate interpretation of the images.

10. The INC gaps in the lama5-/- embryos are striking however, authors do not show whether the BM discontinuities correlate with INC chain instability. If INC detachment coincides with BM gaps, this would strongly support Lama5's role in providing a continuous substrate for both the pLLP and INCs. Please provide LamC1-GFP and/or pan-laminin images specifically beneath INCs.

We thank the reviewer for bringing to our attention this important question. To directly address whether INC chain instability correlates with local BM holes, we examined INCs in the Lamc1-GFP reporter background. This new data is presented in **Fig. 6 and S7**. First, to answer the specific question, we found no direct correlation between INC gaps and BM holes, which are widespread across the epidermal BM in *lama5* mutants (**lines 286-288**).

Importantly, this analysis revealed additional phenotypes that provide further insight into Lama5 function. First, we observed large void spaces between the epidermis and the epidermal BM in *lama5* mutants (asterisks in **Fig. 6H’ and orthogonal view (OV) i**). The position of these voids does not fully match the position of INC gaps either, although there is significant overlap (**Fig. 6H’**). Importantly, these voids are not present in front of the migrating pLLP or outside its migration path. This strongly suggests that they arise from defective re-adhesion following tissue separation by the migrating primordium. These results further suggest that Lama5 is required to provide a continuous substrate for the INCs. Second, we find that translocation of the pLL nerve is impaired, with the nerve frequently remaining above the basement membrane or within these void spaces. Notably, at the level of deposited NMs the nerve of *lama5* mutants often appeared in a transition state, similar to the region where control nerve translocates through the BM. This suggests that nerve translocation may be dependent on mechanical pressure exerted by the overlying tissue. We added these new observations in the result section (**lines 288-307**)

Together, these findings support a model in which Lama5 is required to maintain BM integrity, thereby providing a continuous and functional substrate for both the pLLP and INCs. We believe that the LL-BM adhesion and the skin-BM adhesion are closely linked and discuss this further in the manuscripts’ discussion (**lines 423-428**).

© 2026. Published by The Company of Biologists under the terms of the Creative Commons Attribution License (https://creativecommons.org/licenses/by/4.0/).

11. The text states there is no obvious differences seen in the actin organisation in the leading region of lama5 mutants in Fig. 6H-I". However visually there appears to be increased actin intensity at the base of the protrusions at the cell cortex in mutants. Please quantify actin intensity distribution and/or protrusion base width in the leading region cells.

We thank the reviewer for this careful observation. We agree that the original presentation of these data - based on maximum intensity projections and imprecise protrusion terminology - may have limited the interpretability of these images. We interpret the reviewers' comment to refer to the *lamellipodial* protrusion base width, which we do not feel comfortable quantifying for reasons mentioned before. Further, quantifying actin distribution within lamellipodia or filopodia is challenging due to broad expression of our actin reporter in surrounding tissues.

To address this point, we first replaced the former **Fig. 6H-I"** with better representations of the protrusion behaviour of *lama5* mutants, including single z-slices of filopodia (**Fig. 6I-**J). Alongside, we provide two new analyses regarding (i) filopodial protrusions in *lama5* mutant pLLPs (**Fig. 6K-L - lines 310-313**) and (ii) the actin distribution (**Fig. 7I-N - lines 360-368**). Our new analyses show:

(i) **increased number of filopodial protrusions in *lama5* mutants**. Using the same metrics as for integrin mutants (**Fig. 4E-H**), we observed a slight increase in filopodia number in *lama5* mutants. Notably, this phenotype is reminiscent of that observed in single *itga3b* mutants, suggesting a potential functional interaction between Lama5 and Itga3b in regulating protrusive behavior in the pLLP.

(ii) **supracellular actin distribution is not affected in *lama5* mutants.** We quantified actin distribution along the basal side of the pLLP (yellow line in **Fig. 7I'**) from front to rear. In contrast to myosin II which is enriched at the rear of the pLLP (Yamaguchi et al., 2022 + Figure 3 shown in answer to reviewer 3), we found actin levels peaking between 25 and 50µm from the pLLP leading tip, corresponding to the middle region of the pLLP according to Yamaguchi et al. with levels decreasing toward both the leading and the trailing edges. This distinct supracellular actin distribution was conserved in *lama5* mutants, suggesting no defect in actin distribution both on a supracellular level and regarding the leading region. Notably, this analysis demonstrated a strong shift of actin levels towards the leading region in *lama5;itga6b* mutants, in line with their reduced leading region. Related to major point (15), we believe this data strongly supports a model in which leading region extension is required to propagate the pushing forces from the rear into forward motion.

Together, these analyses indicate that while *lama5* mutants exhibit increased protrusiveness, this is not accompanied by a detectable alteration in the overall actin distribution pattern.

12. The conclusion that lama5 mutants must use alternative receptors because their do not phenocopy integrin mutants is speculative (lines 298-300). Rephrase to avoid conclusions not directly supported by data.

We thank the reviewer for this important point and agree that the original statement was speculative and not sufficiently supported by the data. We have therefore removed the sentence in question and revised the corresponding section to avoid overinterpretation. The text has been rephrased accordingly, to also take into account the newly added data **(Lines 430-450).**

13. Authors state that mild delay occurs in lama5 single mutants (lines 306-307) but itga6b mutants also show a comparable mild delay. Please revise text to reflect that they both share this mild phenotype.

We thank the reviewer for noting this point. We have revised the sentence, to accurately

© 2026. Published by The Company of Biologists under the terms of the Creative Commons Attribution License (https://creativecommons.org/licenses/by/4.0/).

reflect that both *lama5* and *itga6b* mutants display a similarly mild migration delay (**line 328**).

14. Rosette fusion phenotype should acknowledge related work (lines 334-336). Here authors describe leading region splitting and rosette fusion in lama5, itga6b double mutants. Mukherjee et al., 2025, bioRxiv, reported tension-dependent rosette fusion in the pLLP so please cite this as it supports the mechanical basis of this phenotype.

We thank the reviewer for this suggestion. In addition to the reference to this work in the original discussion (now **lines 480-481**), we agree that Mukherjee et al. (2025) should already be cited at the point of phenotype description. The text has been revised accordingly to better contextualize the rosette fusion phenotype (**lines 358-359**).

15. In the discussion the authors' interpretation of front versus rear force contributions requires refinement. The authors' conclusion that a shortened leading region in lama5 mutants supports 'movement driven primarily by pushing from the rear' (lines 435-437) is not fully convincing. Authors show that Itga6b is expressed in the leading region but has the most severe single-mutant phenotype and also loss of itga6b reduces primordium speed more than loss of itga3a or itga3b. Please discuss a model where both rear pushing and front traction contribute and where leading region-specific adhesions provide directional stabilisation rather than just propulsion.

We thank the reviewer for this important point. We agree that a purely rear-driven model is likely oversimplified and have revised the Discussion to reflect a more integrated view of force generation and transmission within the pLLP.

In the revised manuscript, we now propose a model in which rear-to-middle-generated pushing forces and Integrin-mediated front adhesion both contribute to migration. While our data supports the recently proposed "rear-pushing model" (Yamaguchi et al., 2022) according to which major force generation occurs in the rear-to-middle region, our results also highlight a critical role for extension of the leading region in the direction of migration. In particular, the phenotype of *lama5* mutant, strongly enhanced by the loss of Itga6b activity, indicates that leading adhesions operate via two parallel pathways, which compensate efficiently for each other.

We therefore propose that extension and adhesion of the leading region channels and translates rear-generated forces into productive forward movement, acting as a biomechanical wedge, rather than a generator of significant propulsive forces. Consistently, when leading region extension or adhesion is compromised (e.g. in *lama5;itga6b* mutants), migration stalls despite apparently preserved rear-driven pushing. On the other hand, extensive stretching of the leading region in double/triple integrin mutants slowed but did not prevent successful migration.

This revised model, now detailed in the Discussion (**section 3.4**), integrates both rear pushing and front adhesion, and better reflects the division of mechanical roles across the migrating collective.

Reviewer 3:

This work by Moratscheck et al. describes the role of Integrin receptors alpha6b and alpha3 and their extracellular matrix substrate Laminin alpha5 on collective cell migration in vivo, using zebrafish posterior lateral line primordium (pLLP) as a model. The authors provide gene expression and protein localization data for the basement membrane using pan-Laminin and Laminin alpha5 and a reporter line expressing GFP-tagged Laminin gamma1, and uncovered strong deposition in the layer beneath the migrating pLLP cells. They also examined the expression of three of the best-known Integrin alpha subunits binding to basement membrane factors, and uncovered an interesting expression pattern: Integrin alpha3a and b mostly restricted in the rear/lagging end of the migrating pLLP, and Integrin alpha6b in the front/leading end of the migrating pLLP. The authors generated mutations in three different integrin genes, itga3a, itga3b and itg6a, and analyzed an additional

© 2026. Published by The Company of Biologists under the terms of the Creative Commons Attribution License (https://creativecommons.org/licenses/by/4.0/).

mutation in lama5. Among the integrins, itga6b mutations were the only one that slightly delayed migration, but double and triple itga3/itga6b mutations impaired migration more strongly and produced more lateral protrusions. Focal adhesion activation, as assessed by phosphorylated FAK, appears unaffected in the double/triple integrin mutants. lama5 mutations strongly delayed migration, and caused cluster rounding in the pLLP and interneuromast chains. These effects became more pronounced in lama5, itga6b double mutants, with significantly reduced migration speed and cell rounding. This synergistic exacerbation suggests that Laminin alpha5 and Integrin alpha6 promote pLLP migration through mechanisms that are mostly independently of each other.

Overall, this study presents an interesting case study of the role of the extracellular matrix in promoting collective cell migration in vivo. The study is particularly strong in their genetic data; generating and analyzing triple integrin mutants was an impressive undertaking. The analysis method of migration success and cluster morphology is solid. However, the study could benefit from a stronger focus and analysis of the phenotypes from a cell biological perspective.

We thank the reviewer for the careful assessment of our manuscript, and for highlighting both its strengths and areas for improvement. We have addressed these points as far as possible within the scope of this study, as outlined in the point-by-point responses below.

Below are my major comments to strengthen the conceptual advance of the manuscript:
- The mechanistic insight of the loss-of-function phenotypes is still unclear. Considering the distinct expression patterns of the integrins - itga3 in the rear end, itga6b in the front end of the migrating cluster - are the effects of the mutations on cell migration due to genetic redundancy or separate effects on the rear/trailing and front/leading cells of the cluster? Aside from the migrating success and distance, the authors did not report other changes in cellular behaviours in the cluster. It would be interesting to examine in detail the regional effects (e.g. front-rear polarization, protrusion activities in different cells of the cluster, protrusion retraction activities, cell numbers in different regions of the pLLP) of loss of itga3a, itga3b and itga6b alone or in combination. Overall, the study could really benefit from better analysis of the regional differences in cell behaviours and of the rear vs. front polarization within the pLLP.

We thank the reviewer for this insightful comment, which raises important thoughts regarding the spatial and functional organization of integrin activity within the pLLP.

First, we would like to clarify that the expression patterns of *itga3a*, *itga3b*, and *itga6b*are not strictly confined to rear versus front domains. In fact, their expression partially overlaps in the middle region and *itga6b* becomes more restricted from the rear only at a late stage (**Fig. 1I-I''** 48hpf). We have revised the corresponding sections of the manuscript to avoid confusion and better reflect this nuance (**lines 113-115**).

Nonetheless, we agree that these expression patterns raise the possibility of region-specific contributions to the observed phenotypes and make the result of our genetic analysis, which indicates reciprocal functional compensation between *itga3b* and *itga6b*, intriguing. One hypothesis is that the region in which these proteins exert their critical function is not at either extreme, but rather where their expression overlaps.

To further address this point, we have extended our analysis in two key ways:

(i) Polarized Integrin activity via Itgb1b localization:

We assessed the localization of the common ß-subunit Itgb1b in the different α-integrin mutant backgrounds. This new data is presented in Figures **4I-P** and **S5**. Loss of *itga6b* caused a front-to-rear gradient of Itgb1b delocalization, whereas loss of *itga3b* produced the opposite trend. In *3b;6b* double mutants, Itgb1b was broadly delocalized throughout the pLLP. In contrast, loss of *itga3a* had no notable effect. These data suggest that Itga3b and Itga6b dimerize with Itgb1b and polarize its activity. Furthermore, the fact that we observe strong delocalization of Itgb1b in *itga3b;6b* double mutants which exhibit a morphological

© 2026. Published by The Company of Biologists under the terms of the Creative Commons Attribution License (https://creativecommons.org/licenses/by/4.0/).

phenotype (elongated LR) that is quite different from *itgb1b* mutants (rounder than controls, Yamaguchi et al., 2022), highlights the complex adhesion machinery driving pLLP migration that is yet to be fully elucidated. This is now discussed in **lines 430-450**.

(ii) Regional analysis of protrusive behavior:

We now provide a detailed quantification of filopodial dynamics, including length, orientation and abundance in single, double and triple mutants, across the pLLP (new Fig. **4E-H** and **S4A-G**. Consistent with our initial observations, triple integrin mutants exhibited an increase number of filopodia number, which are shorter and less well oriented. Unexpectedly, this phenotype appears to be primarily driven by loss *itga3b* and becomes increased by loss of *itga6b*. In addition, we demonstrate that filopodia at the front of the pLLP are generally longer than those in lateral or trailing regions and that this regional distinction is diminished in itga*3b;6b* and itga*3a;3b;6b* mutants. These new results are described in **lines 182-197**)

Overall, these new data provide important insight into the polarized activity of integrin receptors in the pLLP.

- The phospho-FAK immunostaining appears stronger in the triple mutants, with potentially altered localization. Can the authors comment on whether there are quantifiable changes in its localization? What are the possible downstream effectors if focal adhesion-related factors are not affected? Have the authors tested the localization of other FA factors, such as Vinculin, Talin or Paxillin?

We thank the reviewer for pointing this out. We believe that the apparent increase in pFAK signal in the triple mutant image results from the selection of a suboptimal z-slice showing a locally higher intensity of the *cldnb:GFP* membrane marker, which in turn correlates with higher pFAK staining. To address this, we repeated the experiment to confirm this result and replaced the corresponding panel with more representative images (**now presented in Fig. S6A-C**).

NOTE: We have removed unpublished data that had been provided for the referees in confidence.

Figure 1: pFAK intensity is unchanged in integrin mutants. Average intensity was measured in z-stacks, statistic: non-parametric Wilcoxon rank-sum test on the raw values. Gold diamond shapes represent the mean.

To quantitatively assess potential differences, we measured average pFAK intensity in control and mutants. This analysis did not reveal significant differences between mutants and controls (data shown below). We additionally examined the spatial distribution of pFAK and found higher pFAK levels in the leading region than at the rear of the pLLP, in line with published data demonstrating a similar gradient of phosphorylated paxillin in the pLLP (Yamaguchi et al. 2022). This gradient is preserved in double *itga3b;6b* mutants. The low number of triple *3a;3b;6b* obtained precluded their inclusion in this analysis. We additionally assessed vinculin by immunostaining (**Fig. 6E**). Vinculin localizes to pLLP cell membranes in a more spread, less punctate pattern than pFAK, concentrated at the outer pLLP borders at leading and lateral interfaces with the skin. *itga3b;6b* double and *itga3a;3b;6b* triple mutants displayed reduced vinculin signal at the somite level, suggesting reduced integrin-mediated adhesion at this interface. The overall localization pattern of vinculin within the pLLP appeared otherwise unchanged in integrin mutants. (These new data are presented in **Fig. S6** and described in **lines 200-211**).

- Interneuromast cells change their behaviours to become more protrusive in lama5 mutants. Are there any changes in the neuromast size, organization or individual cell behaviours?

We examined neuromast (NM) number, size and shape in *lama5* mutants. These new data are included in **Fig. S7J-M**. *lama5* mutants deposit on average half a neuromast fewer than sibling controls. However, NM size is unchanged, and we therefore interpret the reduced

© 2026. Published by The Company of Biologists under the terms of the Creative Commons Attribution License (https://creativecommons.org/licenses/by/4.0/).

NM number as a secondary consequence of shortened body axis and correspondingly shortened LL system. We did not detect obvious differences in neuromast shape (roundness) or overall organization, arguing against altered individual cell behaviour.

While NM deposition and organization are unaffected in *lama5* mutants, we observed differences in later aspects of LL development. In particular, ventral displacement of NMs is affected: at 3 dpf, neuromasts in *lama5* mutants are positioned significantly closer to the horizontal myoseptum compared to sibling controls (Figure 2 below). As this observation exceeds the scope of the present study, we have not included it in the manuscript.

NOTE: We have removed unpublished data that had been provided for the referees in confidence.

Figure 2: Ventral displacement of neuromasts is affected in lama5 mutants. (A-B) Alkaline-phosphatase staining at 3dpf in control siblings and *lama5* mutant embryos labels deposited neuromasts. Primary (L1, L2, L3) and secondary neuromasts (LII.1-LII.3) are indicated. (C) Quantified distance of neuromasts to horizontal myoseptum (hm). Scale bars: 200µm.

- Considering the changes in morphology of the migrating clusters in lama5 mutants, could there be a change in the mode of migration, e.g. from lamellipodia-based protrusions to a more amoeboid migration mode, when the ECM is affected? Can the authors test this by closely examining localization or expression of basolateral polarity or actomyosin markers of the migrating cells?

We thank the reviewer for this interesting and important question. First, following re-analysis of our data (and in response to related comments by Reviewer 2), we found that *lama5* mutants exhibited a modest but significant increase in filopodial protrusions, specifically in the lateral domain (**Fig. 6I-L, described in lines 310-313**). This phenotype is reminiscent of that observed upon loss of *itga3b* (**Fig. 4E-G**), suggesting a possible receptor-ligand relationship. While this increase in filopodia could be interpreted as a compensatory response to impaired adhesion and force generation, we do not observe features consistent with a switch in migration mode.

Using actomyosin distribution to assess the migration mode, it was previously shown that myosin II is enriched at the rear of the pLLP, in line with the highest forces detected at the rear (Yamaguchi et al., 2022). We observed the same gradient using a myl12-GFP reporter (Figure 3 shown here). This data is however not included in the manuscript since our mutant fish have not been crossed to this background. To further address this point, we analysed supracellular actin organization along the basal side of the pLLP (**Fig. 7I- N, described in lines 360-368**). In control embryos, actin distribution follows a bell-shape profile, with a peak within a 25 to 50µm window from the leading tip, corresponding to - and in line with - the middle region of the pLLP according to Yamaguchi et al. This distinct supracellular actin distribution was conserved in *lama5* mutants, suggesting no substantial change in migration mode.

NOTE: We have removed unpublished data that had been provided for the referees in confidence.

Figure 3: Myl12-GFP gradient in wild-type embryos recapitulates findings from Yamaguchi et al. 2022. Pearson correlation coefficient based on mean per 2µm-bin (red points +- SD). Loess model with individual datapoints in background.

In contrast, in *lama5;itga6b* double mutants, we observe a shift of actin enrichment toward the front of the pLLP, consistent with their reduced leading region. Given the established rear-localized myosin II distribution and force generation described recently by Yamaguchi et al. We interpret this result as a consequence of persistent rear-derived pushing forces, compressing the actin-rich region towards the front, which fails to extend in the direction of migration.

Finally, by analysing basal actin distribution, we identified striking, actin-rich

© 2026. Published by The Company of Biologists under the terms of the Creative Commons Attribution License (https://creativecommons.org/licenses/by/4.0/).

invadopodia- like protrusions in *lama5;itga6b* mutants that extend deep into the subepidermal space. These structures likely reflect altered cell-matrix interactions and are discussed further in our answer to the next comment.

Together, our data indicate that, although *lama5* loss leads to slightly altered protrusive behavior, there is no evidence for a global transition from lamellipodia-based to amoeboid migration. Instead, the phenotypes are more consistent with defects in adhesion and force transmission within an otherwise preserved migration framework. We have revised the manuscript accordingly to clarify this point and now discuss a model in which leading region extension is required to propagate the pushing forces from the rear into forward motion (Section 3.4 of the discussion).

- What is the significance of the tissue holes seen in lama5 mutants? If the authors can speculate, why is the irregular substrate presence not impacting pLLP migration?

We thank the reviewer for this interesting question. The “tissue holes” observed in *lama5* mutants likely reflect local defects in BM integrity that are independent of the pLLP migration since they are also present underneath the epidermis. Since the loss of Lama5 also leads to a defect in Lamc1 secretion, the holes are likely the result of a defective BM assembly. Based on our genetic analyses, we believe that these irregularities do not substantially impact pLLP migration in *lama5* mutant due to the effective compensation through Itga6b-mediated adhesion. Indeed, combined loss of *lama5* and *itga6b* results in a markedly enhanced phenotype with prominent invadopodia-like structures reaching through BM holes into the subepidermal space (new results presented in **Fig. 7O, video S11 and S12**. Notably, such invasive behaviours are not observed in *lama5* single mutants, suggesting that Itga6b contributes to maintaining appropriate cell-matrix interactions and constrains aberrant protrusive activity at sites of BM disruption.

These observations suggest that, in *lama5* mutants, residual or alternative adhesion mechanisms involving *Itga6b* are sufficient to maintain effective coupling between the pLLP and its substrate, thereby preserving migration despite local BM defects. When this compensatory mechanism is removed, cells appear to exploit BM discontinuities, resulting in invasive protrusions hindering productive forward migration.

We have incorporated this interpretation into the revised manuscript (**lines 456-461**), where we discuss how integrin-mediated adhesion not only supports migration but also spatially restricts protrusive activity to ensure coordinated collective movement

- The authors concluded that the effects of Laminin alpha5 and Integrin alpha6 and alpha3 are mostly independent of each other; in the Discussion, they wrote that the primary ligands of the Integrins are not Laminins, and the primary receptor for Laminin alpha5 is not Integrin alpha3 and 6. Thus, there are missing pathways by which the ECM ligands and receptors interact with each other. Can the authors provide some data as to what these missing links could be: from in situ, immunostaining or citing known literature on expression patterns of other key focal adhesion-related molecules? For instance, the authors speculated about Integrin alpha7 in the Discussion - what is the expression pattern of this alpha subunit? Is there also a possibility for an Integrin- independent mechanism in this migrating cell type by which Laminins exert their effects?

We thank the reviewer for this important point. Indeed, we conclude from our study that the effects of Lama5 and Itga3/Itga6b are partially, but not fully, independent from each other. On the one hand, the loss of Itga3a/b activity does not enhance the *lama5* mutant phenotype suggesting a potential receptor-ligand relationship. This is supported by our new data showing an increased protrusiveness in both *lama5* and *itga3b* mutants. On the other hand, the strong genetic interaction between Itga6b and Lama5 indicate a complex, multilayered adhesion machinery with additional components. To address this, we have carefully re-evaluated possible players in the pLLP adhesion machinery:

- **Integrin α7**: As the last laminin-binding α-integrin which we did not address in our

© 2026. Published by The Company of Biologists under the terms of the Creative Commons Attribution License (https://creativecommons.org/licenses/by/4.0/).

study, *itga7* represents an important candidate. However, based on available *in situ* hybridization (ISH) and single-cell transcriptomics data, we found no evidence for *itga7* expression in the lateral line (Farnsworth et al., 2020; Sur et al., 2023). We note however that we cannot exclude induction of *itga7* expression in the pLLP as a compensation mechanism (i.e. via transcriptional adaptation) to the loss of integrins a3/a6.

- **Additional ß-integrin subunits:** other integrin ß-subunits, namely *itgb4* and *itgb6,* are expressed in the migrating pLLP, albeit at low levels and with strong expression in the overlying epidermis (Yamaguchi et al., 2022). Itga6 is the only known partner of Itgb4, suggesting a potential role for this heterodimer in the pLLP. However, preliminary data from our lab indicates that *itgb4* has effects beyond the function of *itga6b* in the pLLP. The α-partner of *itgb6*, *itgav*, is not clearly expressed in the LL based on single-cell transcriptomics data and ISH (Farnsworth et al., 2020; Sur et al., 2023; Patra et al., 2025). We plan to investigate these other integrin players in the future.
- **Non-integrin laminin receptors:** we also note that several non-integrin adhesion receptors capable of binding laminin are expressed in the pLLP. This include *bcam*, *dag1* (dystroglycan) and heparan sulfate proteoglycans (Thisse and Thisse, 2004; Thisse et al., 2008; Venero Galanternik et al., 2015). These molecules represent plausible candidates for mediating integrin-independent interactions with the ECM and may contribute to the residual adhesion and migration observed in our mutants.

We have revised the discussion accordingly to (i) remove *itga7* as a potential player in the pLLP adhesion machinery as this is not supported by any published expression data and
(i) add other beta-subunits and non-integrin adhesion receptors to better reflect the complexity of the adhesion machinery (**lines 439-450**).

Overall, while our current study focuses on *itga3*, *itga6b*, and *lama5*, our findings strongly suggest that pLLP migration relies on a redundant and multifactorial adhesion network, the detailed dissection of which will require further investigation.

- Are there specific experiments that the authors can do to more strongly support their hypothesis of the rear-driven migration? For instance, AFM, visualization of actin flow or Integrin beta1 and Talin1 localization in relation to the migrating cluster, as performed in Yamaguchi et al. 2022. In particular, it is very interesting that the mutations in integrin alpha3a and 3b, which were enriched in the lagging end of the pLLP, had the mildest effects on migration; can the authors comment on why this could be (genetic redundancy with other Integrin alpha subunits, lack of need for Integrins in the rear end propelling of the cell clusters)?

We thank the reviewer for this interesting suggestion and for highlighting the relevance of directly probing the mechanical basis of pLLP migration. We agree that approaches such as AFM measurements, visualization of actin flow, or detailed analysis of additional focal adhesion components (e.g., Talin1) would provide valuable insight into force generation and transmission within the migrating cluster.

While these experiments are beyond the scope of the current study, we have extended our analysis to better address the underlying mechanism using complementary approaches:

First, we analysed the localization of the common ß-integrin subunit *Itgb1b* in the different α-integrin mutant backgrounds (**new Fig. 4I-N, Fig. S5**). We find that loss of *itga6b* or *itga3b* leads to regionally biased delocalization of *Itgb1b*, while combined loss results in a broad delocalization of *Itgb1b* across the pLLP. These data support the idea that integrin- mediated adhesion is spatially organized and contributes to coordinated force transmission across the tissue.

Second, we performed quantitative analyses of supracellular actin distribution (**new Fig. 7I-N**). Consistent with previous work (Yamaguchi et al., 2022), we observe a distinct actin

© 2026. Published by The Company of Biologists under the terms of the Creative Commons Attribution License (https://creativecommons.org/licenses/by/4.0/).

distribution along the pLLP axis. Importantly, this global pattern is largely preserved in the mutant conditions. This suggests that the general migration mode relying on force generation behind the leading region is maintained in the described mutants, and that migration defects primarily result from how these forces are transmitted into productive forward movement.

Our results suggest that integrin function in the pLLP is not strictly partitioned into front- versus rear-specific roles, but rather operates as a distributed, partly overlapping and partially redundant system that ensures effective coupling between tissue-intrinsic forces and the extracellular matrix. In this framework, the mild phenotype of *itga3a* and *itga3b* mutants, despite their enrichment in the trailing region, might be explained by their function being compensated by other adhesion systems, including Itga6b. Indeed, our genetic data demonstrate reciprocal compensation between *Itga3a/3b* and *Itga6b*, and only combined loss leads to pronounced defects.

We have revised the Discussion to better reflect this interpretation and to more explicitly integrate our findings with the rear-driven migration model proposed by Yamaguchi et al. (2022), while also outlining limitations and future directions to directly probe force generation and transmission in this system (**Discussion section 3.4**).

Minor comment:

The title "Patterned Integrin-Laminin adhesion..." is a little vague, clearer rewording might be needed to better describe the work.

We thank the reviewer for this suggestion and changed the title to: "Laminin α5 and integrin α3/α6 coordinately regulate collective cell migration *in vivo*"

References

Farnsworth, D. R., Saunders, L. M. and Miller, A. C. (2020). A single-cell transcriptome atlas for zebrafish development. *Developmental Biology* **459**, 100-108.

Mukherjee, A., Hilzendeger, M., Rinvelt, A., Fatma, S., Schupp, M., Dalle Nogare, D. and Chitnis, A. B. (2025). Signaling and Mechanics influence the number and size of epithelial rosettes in the migrating zebrafish Posterior Lateral Line primordium. *bioRxiv*.

Nadol, J. B., Gibbins, J. R. and Porter, K. R. (1969). A reinterpretation of the structure and development of the basement lamella: An ordered array of collagen in fish skin. *Developmental Biology* **20**, 304-331.

Patra, C., Rayrikar, A., Wagh, G., Kleefeldt, F., Roshanbinfar, K., Cop, F., Nikolic, I., Schmidt, M. H. H., Acker-Palmer, A., Ergün, S., et al. (2025). Nephronectin Is Required for Vascularization in Zebrafish and Sufficient to Promote Mammalian Vessel-Like Structures in Hydrogels for Tissue Engineering. *JAHA* **14**, e037943.

Sur, A., Wang, Y., Capar, P., Margolin, G., Prochaska, M. K. and Farrell, J. A. (2023). Single-cell analysis of shared signatures and transcriptional diversity during zebrafish development. *Developmental Cell* **58**, 3028-3047.e12.

Thisse, B. and Thisse, C. (2004). Fast Release Clones: A High Throughput Expression Analysis. ZFIN Direct Data Submission. (http://zfin.org).

Thisse, B., Wright, G. J. and Thisse, C. (2008). Embryonic and Larval Expression Patterns from a Large Scale Screening for Novel Low Affinity Extracellular Protein Interactions.

Venero Galanternik, M., Kramer, K. L. and Piotrowski, T. (2015). Heparan Sulfate Proteoglycans Regulate Fgf Signaling and Cell Polarity during Collective Cell Migration. *Cell Reports* **10**, 414-428.

© 2026. Published by The Company of Biologists under the terms of the Creative Commons Attribution License (https://creativecommons.org/licenses/by/4.0/).

Watanabe, K. and Tachibana, T. (1973). Transmission and scanning electron microscopic study of adepidermal granules of teleosts and amphibia. *Z.Zellforsch* **142**, 163-170.

Weiss, P. and Ferris, W. (1954). ELECTRON-MICROSCOPIC STUDY OF THE TEXTURE OF THE BASEMENT MEMBRANE OF LARVAL AMPHIBIAN SKIN. *Proc. Natl. Acad. Sci. U.S.A.* **40**, 528-540.

Yamaguchi, N., Zhang, Z., Schneider, T., Wang, B., Panozzo, D. and Knaut, H. (2022). Rear traction forces drive adherent tissue migration in vivo. *Nat Cell Biol* **24**, 194- 204.

Second decision letter

MS ID#: dev.205312R1

MS TITLE: Laminin α5 and integrin α3/α6 coordinately regulate collective cell migration in vivo

AUTHORS: Anna Mertens, Nicola Moratscheck, Petra A. Klemmt, Chaitanya Dingare, Melanie Heyde, Marion Basoglu, Stefan Eimer and Virginie Lecaudey

Dear Dr Lecaudey,

I am happy to let you know that your manuscript has been accepted for publication in Development, pending our standard publication integrity checks.

Please also see a few minor comments from Reviewer 3, which we suggest you incorporate into the final manuscript.

Reviewer 1

Advance summary and potential significance to field

The revisions in response to the other two reviewers added to the clarity and comprehensive nature of this study. It is ready to be published in my opinion.

Reviewer 2

Advance summary and potential significance to field

The authors have addressed my main concerns. I consider it suitable for publication without further modification.

Reviewer 3

Advance summary and potential significance to field

I thank the authors for addressing almost all my and Reviewer 2's comments. Overall, the authors made strong efforts to experimentally address the concerns that strengthen the resubmitted version of the manuscript. The data on the directionality and numbers of the protrusions, as well as additional data on the morphology of the migrating clusters add valuable understanding of the processes affected by the loss of Integrins. The inclusion of Study Limitations also provides good understanding of potential future directions and still missing gaps in knowledge.

© 2026. Published by The Company of Biologists under the terms of the Creative Commons Attribution License (https://creativecommons.org/licenses/by/4.0/).

Minor Comments:
- Move Figure 2 (single mutant pLLP data) as new Figure S3 (current Fig S3 becomes S4, and so on).
- Section 2.2 is very long. Perhaps the authors could break it down into two sections, with a new Section 2.3 focusing on the filopodia protrusions and loss of directionality in the double and triple integrin mutants.
- I am not certain whether the term "Integrin b1b delocalization" is commonly used and is the accurate term to describe the phenotype in Figure 4. I would suggest describing it as "Integrin b1b membrane localization appears reduced."
- Can the authors include anything quantitative to measure protrusion dynamics / extension and retraction rates from Figure S5F? This also might give insight into the stability of the protrusions in the different genetic backgrounds.
- Considering the authors propose the rear traction-driven mode of migration drives pLLP migration, the authors should include statements regarding the potential role of the filopodia protrusions that were observed at higher rates in the triple integrin mutants in the Discussion. For instance, they might play a role in sensing growth factors or tissue stiffness in the environment to move forward. This section on filopodia can be fit into Section 3.4.

Second revision

Author response to reviewers' comments

Reviewer 1: The revisions in response to the other two reviewers added to the clarity and comprehensive nature of this study. It is ready to be published in my opinion.

Reviewer 2: The authors have addressed my main concerns. I consider it suitable for publication without further modification.

Reviewer 3: I thank the authors for addressing almost all my and Reviewer 2's comments. Overall, the authors made strong efforts to experimentally address the concerns that strengthen the resubmitted version of the manuscript. The data on the directionality and numbers of the protrusions, as well as additional data on the morphology of the migrating clusters add valuable understanding of the processes affected by the loss of Integrins. The inclusion of Study Limitations also provides good understanding of potential future directions and still missing gaps in knowledge.

We thank all Reviewers for their positive feedback.

Minor Comments:
- Move Figure 2 (single mutant pLLP data) as new Figure S3 (current Fig S3 becomes S4, and so on).

We thank Reviewer 3 for this suggestion. Figure 2 has been moved the to the supplementary material as new Figure S2 (to maintain the correct order of appearance of the Fig. S2 relative to Fig. S3 in the text), and all subsequent figures have been renumbered accordingly. All modified figure numbered are highlighted in red

- Section 2.2 is very long. Perhaps the authors could break it down into two sections, with a new Section 2.3 focusing on the filopodia protrusions and loss of directionality in the double and triple integrin mutants.

We agree with Reviewer 3 and have divided the section accordingly. Section „2.2. itga3a, itga3b and itga6b are required during pLL development“ is now followed by Section „2.3. Combined loss of *itga3b & itga6b* increases filopodia protrusiveness and loss of directionality“. (lines 184-185)

- I am not certain whether the term "Integrin b1b delocalization" is commonly used and is the accurate term to describe the phenotype in Figure 4. I would suggest describing it as "Integrin b1b membrane localization appears reduced."

© 2026. Published by The Company of Biologists under the terms of the Creative Commons Attribution License (https://creativecommons.org/licenses/by/4.0/).

While we believe the term „delocalization“ is not inaccurate, we appreciate the Reviewer’s concern regarding ist clarity. We deliberately avoided phrasing such as „reduced membrane localization“, as our imaging resolution and quantification approach do not allow us to reliably assess the absolute amount of Itgb1b at the cell membranes. To adress this point, we now use the more neutral term „mislocalized“. We have also clarified our definition of „mislocalization“ in the text, specifying that it refers to signal that is less membrane-associated compared to the control condition (line 227-230, 232, 234, 237, 238, 464, 484, 531, 660, 671, 971 also see changes in lines 449 and 453-454).

- Can the authors include anything quantitative to measure protrusion dynamics / extension and retraction rates from Figure S5F? This also might give insight into the stability of the protrusions in the different genetic backgrounds.

We agree that quantitative information on protrusion dynamics would add value to the study. However, several technical limitations currently preclude robust quantification:
(i) The actin reporter *Tg(cxcr4b:Ruby-Lifeact)* exhibits substantial variability in signal intensity and also labels the myoseptum, making it difficult to reliably distinguish filopodia from background signal.
(ii) the required temporal resolution to capture filopodia dynamics impose a perfectly flat orientation of the embryo/pLLP to limit z-stack range to a minimum.
(iii) the low proportion of double (1:16) and triple (1:64) mutants further limits the feasibility of acquiring datasets of sufficient size for meaningful quantification of filopodia dynamics in the different genetic backgrounds.
Despite these constraints, we believe that our quantitative analysis of still images clearly demonstrates that loss of integrins leads to increased, less directional filopodial protrusions.

- Considering the authors propose the rear traction-driven mode of migration drives pLLP migration, the authors should include statements regarding the potential role of the filopodia protrusions that were observed at higher rates in the triple integrin mutants in the Discussion. For instance, they might play a role in sensing growth factors or tissue stiffness in the environment to move forward. This section on filopodia can be fit into Section 3.4.

We thank the Reviewer for this suggestion. We have now expanded the Discussion (Section 3.4) to address the potential functional role of filopodia in this in the pLLP rear. Specifically, we discuss the possibility that (i) rear filopodia in WT are involved sensing of guidance cues or mechanical properties in the environment, which could contribute to directional migration, and (ii) the increased, less directional filopodia in triple mutants might either reflect changes in these environmental cues and contribute to the slowed migration or be a secondary consequences of the migration defects resulting in increased probing (lines 491 to 496).

© 2026. Published by The Company of Biologists under the terms of the Creative Commons Attribution License (https://creativecommons.org/licenses/by/4.0/).
